# Self-Other Mergence in the Frontal Cortex during Cooperation and Competition

**DOI:** 10.1016/j.neuron.2016.06.022

**Published:** 2016-07-20

**Authors:** Marco K. Wittmann, Nils Kolling, Nadira S. Faber, Jacqueline Scholl, Natalie Nelissen, Matthew F.S. Rushworth

**Affiliations:** 1 Department of Experimental Psychology, University of Oxford, South Parks Road, OX1 3UD Oxford, UK; 2 Department of Psychiatry, University of Oxford, Warneford Hospital, OX3 7JX Oxford, UK; 3 Centre for Functional MRI of the Brain, University of Oxford, John Radcliffe Hospital, OX3 9DU Oxford, UK

**Keywords:** fMRI, social cognition, theory of mind, cooperation, competition, dorsomedial prefrontal cortex, decision making

## Abstract

To survive, humans must estimate their own ability and the abilities of others. We found that, although people estimated their abilities on the basis of their own performance in a rational manner, their estimates of themselves were partly merged with the performance of others. Reciprocally, their ability estimates for others also reflected their own, as well as the others’, performance. Self-other mergence operated in a context-dependent manner: interacting with high or low performers, respectively, enhanced and diminished own ability estimates in cooperative contexts, but the opposite occurred in competitive contexts. Self-other mergence not only influenced subjective evaluations, it also affected how people subsequently objectively adjusted their performance. Perigenual anterior cingulate cortex tracked one’s own performance. Dorsomedial frontal area 9 tracked others’ performances, but also integrated contextual and self-related information. Self-other mergence increased with the strength of self and other representations in area 9, suggesting it carries interdependent representations of self and other.

## Highlights

• People confuse their own performance with the performance of others
• Cooperation leads to positive and competition to negative self-other confusion
• Confusion is reflected in area 9 indicating interdependent self-other processing
• Learning from own performance history is represented in perigenual ACC

## Teaser

Wittmann et al. find that we confuse own performance with the performance of others, depending on whether we cooperate or compete with them. This confusion is reflected in area 9, indicating it interdependently processes information about ourselves and others.

## Introduction

Social environments require humans and other primates to monitor others (Chang et al., 2013a, Chang et al., 2013b, Ruff and Fehr, 2014) and to know not only their own abilities, but also the abilities of others. This knowledge guides establishment of social dominance hierarchies (Zink et al., 2008) and can be linked to features of brain structure and function (Noonan et al., 2014, Sallet et al., 2011). It guides animals’ choices in a powerful way. For example, the decision to engage in fundamental modes of social interaction such as cooperation or competition with a conspecific is guided by knowledge about their abilities and social status relative to one’s own (Wang et al., 2011). Estimating abilities of both self and others on the basis of past performance may be particularly important for humans, as they are able to coordinate and execute multi-step tasks such as building a shelter together with others (Misyak et al., 2014, Tomasello et al., 2005) or making complex decisions in groups (Kerr and Tindale, 2004). Moreover, they have to use this knowledge flexibly because social alliances can change very quickly: a competitor within one’s own company might quickly become a cooperator when competing with a different company.

It has been a long-standing idea in psychology that humans derive expectations about whether they will succeed in a given task from their past task performance (Bandura, 1977). At the same time, people do not learn about themselves in isolation but relative to their social environment. Comparisons with other people can be used as an effective means for self-evaluation (Festinger, 1954, Mussweiler, 2003), and, conversely, people base judgments of other people on knowledge of their own traits (Allport, 1924, Krueger, 2010). However, the social influence on judgments of self and others can vary. We might be influenced more strongly by others simply because we like them (Heider, 1958); we might, by default, evaluate members of our own group more positively than members of a different group (Brewer, 1979), and we might perceive others as more similar to us when we cooperate with them than when we compete with them (Toma et al., 2010). In sum, learning about self and others is often based on all three aspects: objective experience, self/other comparisons, and the social context.

In the field of neuroscience, we are only beginning to explore the computational and neural mechanisms that underlie how people learn about the abilities of other people (Boorman et al., 2013), but even less is known about how we learn about our own abilities. Reward-related brain signals scale with the payoff for oneself relative to the payoff of other people (Fliessbach et al., 2007). Recently, it has been shown that one’s own choice preferences can be biased toward the observed choice preferences of other people (Garvert et al., 2015, Nicolle et al., 2012) and that this depends on the identity of the other person consistent with psychological theories (Izuma and Adolphs, 2013). However, unlike choice preferences, where there is no clear right and wrong, there is often objective information available about our own abilities. For instance, the time one needs to run 100 m ought to be well predicted by the previous occasions on which one ran 100 m. In this sense, ability judgments can be based on objective performance feedback attributable unambiguously to ourselves.

Here, we test, first, whether we estimate our abilities from monitoring our performance over time just as we estimate the values of our actions from monitoring their outcomes (Daw et al., 2011). We show that the history of an individual’s performance is indeed used to estimate their ability. Second, we test whether ability estimated for oneself is also dependent on the performance of other people. We find that, surprisingly, the performances of other people also influence individuals’ assessments of their own abilities. When we are cooperating with someone who performs well, our estimates of our own ability are inflated, and when we are cooperating with someone who performs poorly our ability estimates are depressed. Reciprocally, the ability of another individual is estimated not only from the other’s performance, but also from one’s own performance. We demonstrate that such self-other mergence not only impacts on people’s subjective evaluation of themselves and others, but even affects how they subsequently adjust their performance. We refer to ability estimation for self and others based on their respective performance history as “appropriate,” while we refer to the misattribution of past self-related performance when estimating others’ abilities (or other-related performance to estimate one’s own ability) as self-other mergence (SOM; see Figure 2A for an illustration).

Human subjects performed an experiment in an MRI scanner. Distinct regions in medial frontal cortex (Neubert et al., 2015) tracked the estimated abilities of self (perigenual anterior cingulate cortex; pgACC) and other (area 9) (Kelley et al., 2002, Mitchell et al., 2006). SOM increased with the strength of self and other representations in area 9; its activity predicted both how much self-judgments were related to the other player, and how much other judgments were related to one’s own performance. This suggests that area 9 does not simply represent other people’s perspectives independently of our own (Amodio and Frith, 2006), but instead it represents self and others in an interdependent fashion.

## Results

### Experiment Structure

**Figure 1:**
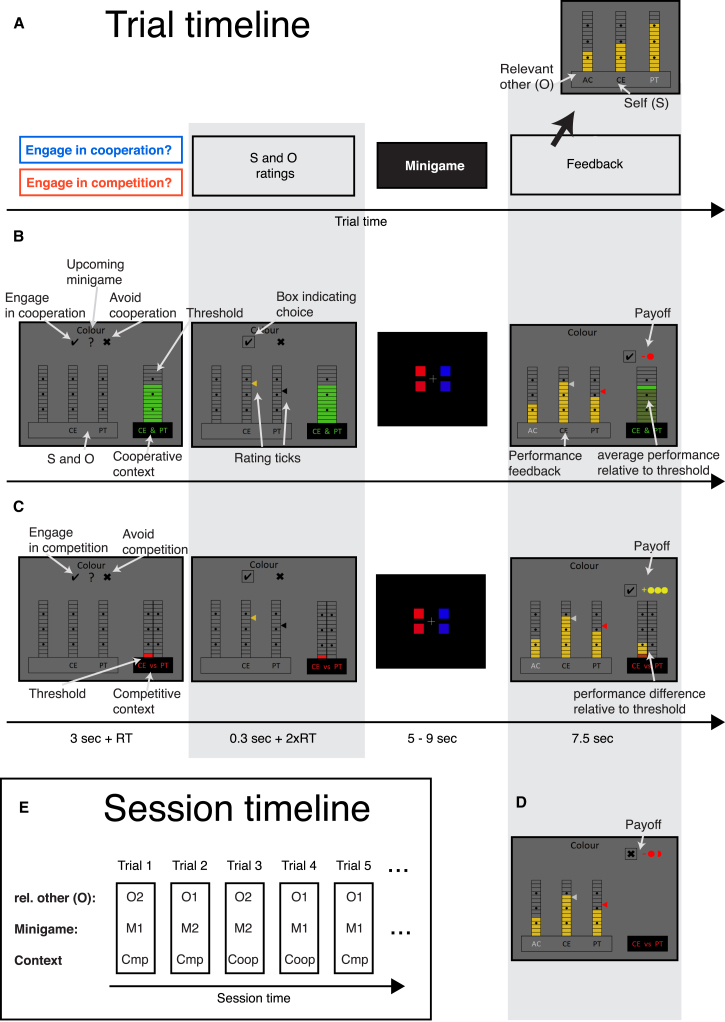
Task Design (A) Schematic of trial events. On every trial, all players played a minigame, after which parametric performance feedback was provided (upper right; higher bars indicate better performance) that enabled performance to be learned. Letters indicate subject initials for Self (S; middle position) and two other players. A relevant other (O) was pre-specified on each trial but changed between trials. Ratings and minigames were embedded in engage/avoid decisions that established either a cooperative or competitive context. (B) Trial timeline for an example cooperative trial. Trials start with the presentation of S, O, the upcoming minigame, social context, and the threshold. A decision is made about whether to cooperate or not (in this case the subject decided to cooperate). After the decision, subjects provided, in randomized order, *S-ability* and *O-ability* ratings. Then all players play a minigame (independent of choice or ratings). In the feedback phase, the payoff (−1 in this instance) is determined by the disparity between the performance average of S and O (9) and the threshold they were asked to exceed (10). Payoffs were parametric and could be positive, zero, or negative. (C) Trial timeline for an example competitive trial. Annotations in the figure panel indicate elements differing from the cooperative example in (B). Again, an engage choice is made but now the decision is whether or not to compete. In this case, the subject chose to compete. Payoff (+3) is determined by the difference between the performance difference of S and O (4) and the threshold (1). (D) If subjects decided not to cooperate or to compete (avoid choices), they nonetheless went on to make ratings and perform minigames. In the feedback phase, the context was still displayed (competitive in this example), but no threshold was shown, and the payoff was independent of performance scores. On such trials, subjects either won or lost 1.5 points with equal probability (i.e., the expected value was zero). Hence, choices should only depend on the expected value of cooperating or competing (depending on which context was indicated). (E) Schematic of session timeline. Each trial is characterized by one of two other players having the role of relevant other (O1/O2) and one of two possible minigames (M1/M2) and has either a cooperative (Coop) or compete (Cmp) social context. All three trial features were experimenter determined and pseudorandomly interleaved.

On each trial, subjects performed a reaction-time minigame. They were told that two other players independently performed the minigame at the same time (Figures S1–S3). We explain the nature of the minigames in detail in Figure S3. The minigames’ precise nature is less critical than the fact that they provided a vehicle to investigate how subjects developed an estimate of their ability that was based on their performance and how this changed depending on interactions with two other players whose performances they also saw. On each trial in the experiment, subjects performed a short trial of a minigame and parametric feedback about their own performance and the performance of the other players was provided at the end of each trial (Figure 1A). Subjects could use the performance feedback to form ability estimates for self and the two others over the course of the experiment.

We used pre-determined performance feedback schedules to carefully match observed performance for self and others and to keep them stable across subjects. This ensured that performance learning for self and others were comparable and that individual differences in task behavior were interpretable. Subjects were told that the performance feedback reflected their objective performance mapped on a 15-point performance scale and that the previously established mapping was the same for all players. Therefore, subjects received explicit and independent performance feedback for all players. Using not only one but two minigames (“time task” and “color task”) in pseudorandom trial order made it possible to have, on the one hand, slowly drifting performance shifts within a minigame (as abilities are thought to be relatively stable features [Boorman et al., 2013]) but, on the other hand, reduced sequential correlations across trials (by switching between minigames that were performed at different levels; Figure 1E) and ensured a full parametric range of performance feedback, thereby making it possible to perform event-related fMRI analysis. Finally, having two minigames allowed us to establish the generality of our findings.

Each trial was performed either in a cooperative or a competitive context. For example, on some trials subjects were given the opportunity to cooperate with one of two other players in the next run of the minigame (Figure 1B). This meant that their own performance score and the other player’s performance score would be summed together, and if it exceeded a threshold indicated on the screen (varying trialwise), then points were awarded (which were translated into monetary reward at the end of the experiment). Subjects could first decide whether to engage in cooperation or whether to avoid it (engage/avoid decision). If they avoided cooperation, then the points awarded were randomly distributed around zero. The other player was pre-specified on each trial and is referred to as “relevant other” (O) as opposed to the “self” (S). Specifying only one of the other players as O on any given trial, and changing this from trial to trial, also helped reduce sequential dependencies between performance estimates on successive trials. Whether or not a subject collaborates on any trial should depend on the subject’s estimate of their own ability, their estimate of the other player’s ability, and the threshold that has to be reached.

On other trials, subjects were given the opportunity to compete the relevant other, O, in the next run of the minigame (Figure 1C). This meant that the difference between their own performance score and the other player’s performance score would be taken, and if it exceeded a threshold indicated on the screen, then points were awarded. Subjects could first decide whether to engage in competition or whether to avoid it (engage/avoid decision). If they avoided competition then the points awarded were randomly distributed around zero. Again, which of the two other players was O varied from trial to trial. And again, whether or not a subject competes should depend on the subject’s estimate of their own ability, their estimate of the other player’s ability, and the threshold score that has to be reached.

In summary, the engage/avoid decision created either a cooperative or a competitive relationship between S and O; O became either an ally or an opponent for S on that trial (Figures 1B and 1C). The context varied pseudorandomly across trials making sure that slowly drifting ability estimates were comparable between competitive and cooperative trials. Importantly, the inclusion of the engage/avoid decision and the threshold enabled us to distinguish S and O related brain activity from brain activity related to reward processing.

So that we could measure subjects’ absolute ability estimates, subjects were asked on each trial to rate the performance they expected for the current trial for themselves (*S-ability*) and the relevant other (*O-ability*; Figures 1A–1C). *S-ability* and *O-ability* were assessed via two independent ratings (in random order) prior to performing the minigame. Having the ability ratings embedded in either a cooperative or a competitive context allowed us to test whether past performance of S and O had differential effects on the *O-ability* and *S-ability*, respectively. We used ratings in which subjects rated the players relative to a rating marker, which was updated over trials using a staircasing procedure (see “Ability ratings” in Experimental Procedures) to maximize both informativeness and speed of the ratings (and therefore trial number and statistical power). The inclusion of two different other players who took turns to be the relevant other in a trial ensured that S’s and O’s performance feedback and associated ability estimates were statistically decorrelated allowing identification of neural correlates in an unconfounded manner (Figure S5A).

### Appropriate Ability Estimation and Self-Other Mergence

**Figure 2:**
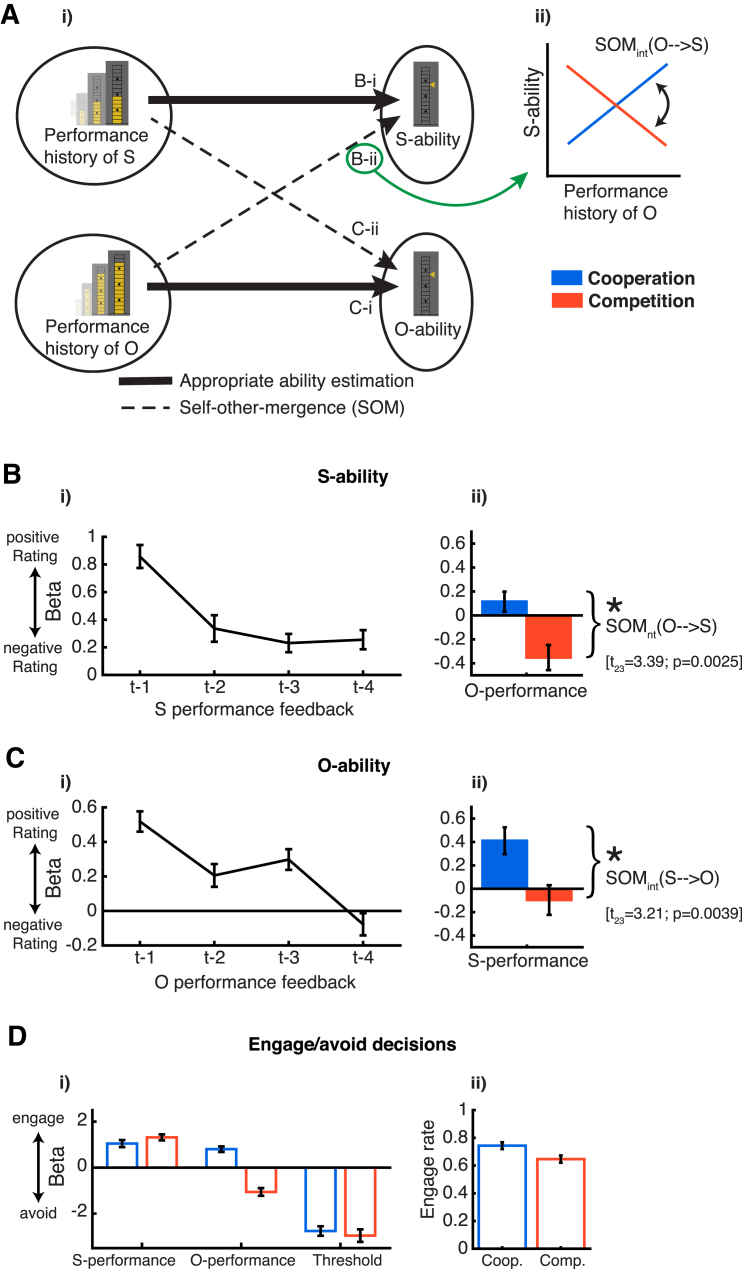
Behavioral Results Blue and red indicate cooperation and competition trials in all panels. (A) Schematic of appropriate ability estimation and self-other mergence (SOM) (i). Letters on arrows refer to subsequent panels showing analysis of appropriate ability estimates (B-i and C-i) and SOM effects (B-ii and C-ii). *S-ability* and *O-ability* refers to trialwise ratings of oneself (S) and relevant other (O). (ii) Illustration of a context-dependent SOM effect where O’s past performance influences *S-ability* estimates. *S-ability* estimates are inflated or depressed when O’s performance history has been, respectively, good or poor in cooperative contexts. However, the inverse is the case in competition: good performers decrease *S-ability* estimates. Positive and negative beta weights in B-ii (and analogously for *O-ability* in C-ii) reflect the increasing and decreasing slopes in this illustration. (B and C) Agent appropriate estimation of ability in (B-i) and (C-i): *S-ability* and *O-ability* on trial t were based on recent S and O performance feedback, respectively. Panels show beta weights of a logistic GLM, applied to each subject and averaged over subjects (same for subsequent panels). Ratings were more strongly based on the recent performance feedback received by the appropriate player (i.e., *S-ability* ratings reflect S’s past performance and *O-ability* ratings reflect O’s past performance). SOM of performance in (B-ii) and (C-ii): we found an influence of *O-performance* on *S-ability*, controlling for the effect of *S-performance*. This effect reversed with social context such that the estimation of one’s own ability was either inflated (in cooperation) or depressed (in competition) when paired with a high performer. Similarly, *S-performance* influenced estimates of *O-ability*, controlling for the influence of *O-performance* (significant SOM_int_; p values for both analyses calculated in an interaction analysis from Figure S4A; same y axes as in (i). Note that different GLMs were used for (i) and (ii) (see main text and Experimental Procedures). (D) Engage/avoid decisions. (i) *S-performance*, *O-performance*, and threshold influenced decisions to cooperate or compete in a rational manner. In particular, O is weighted in reverse fashion depending on whether O is an ally in a cooperative context or an opponent in a competitive context. (ii) Rate of engage choices for cooperative (“Coop.”) and competitive (“Comp.”) trials (error bars are mean ± SEM).

We analyzed rating and decision data using logistic general linear models (GLMs) applied first to each subject separately and then averaged resulting regression weights (beta weights) across subjects (Figure 2). After testing whether both ability judgments in the rating data and engage/avoid choices were based on the previous performances of the appropriate players, we went on to investigate self-other mergence (Figure 2A).

First, we tested which information subjects used to estimate self and others’ abilities. Analysis of the rating data showed that *S-ability* and *O-ability* were based on the performance history of S and O, respectively. This indicates that subjects indeed used performance feedback to generate predictions about their subsequent minigame performance (Figures 2B-i and 2C-i). Recent performance feedback was predictive of subjects S and O ratings. However, performance that had occurred more remotely in time had much less of an impact on the subjects’ ability estimates. This finding that recent and remote events have greater and lesser impacts, respectively, is similar to the finding that action value estimates are based more on recent reward in reinforcement-type learning (Sutton and Barto, 1998). Therefore, to summarize the performance histories that subjects had observed, we fitted a standard reinforcement learning (RL) model individually for each subject (Supplemental Experimental Procedures 1). *S-performance* and *O-performance* from the RL models were estimates of expected performance and represented recency-weighted averages of the performance feedback (not the true performance) of S and O.

Next, we examined whether *S-performance* and *O-performance* from the RL models predicted decisions to engage in cooperation/competition. We found that a better *S-performance* increased the likelihood of cooperating and competing, while a better *O-performance* led to increased cooperation and decreased competition (Figure 2D). The third factor, the threshold that subjects had to exceed on each trial, not surprisingly also influenced behavior; a higher threshold led to less engagement in both cooperative and competitive contexts. As incentivized by the experimental design, subjects’ engage rates were around 70% (Figure 2D-ii), and we found a slight preference for cooperation compared to competition (t_23_ = 3.82; p < 0.01). This preference might be related to the experiment’s payoff structure. However, it is also consistent with suggestions that a normative cooperative bias exists in social interactions promoting cooperation over selfish behavior (Boyd and Richerson, 2009). This shows that choices when to cooperate and when to compete, like the subjects’ ability ratings, were strongly based on the previous performances of S and O. It also indicates that our competition/cooperate manipulation had the expected impact on subjects’ behavior: subjects preferred to cooperate with high performers and to compete with low performers, as they should indeed have done in order to maximize their reward in the experiment.

Finally, we investigated whether subjects’ own ability estimates were also related to O’s past performance. We focused our analysis of SOM on trials where subjects chose to engage in cooperation or competition. Controlling for *S-performance*, we assessed the influence of *O-performance* on *S-ability*. To do this, we tested whether, in a GLM which contained factors indexing past performance of S, past performance of O were predictive of subjects *S-ability* ratings. Specifically, we tested whether *S-ability* judgments were indeed biased toward the performance levels of O in cooperation, but away from them in competition. We found that, indeed, during cooperation *S-ability* increased and decreased in tandem with *O-performance*: independent of subjects’ own performance, subjects evaluated themselves more positively when the other player performed well and more negatively when the other performed badly. This was reversed in competition: subjects evaluated themselves more negatively when competing with a high compared to a low performer, as demonstrated by an interaction effect (SOM_int_(O→S), t_23_ = 3.39; p = 0.0025; see Figure S4A for full details and summary in Figure 2B-ii). We found *O-ability* was influenced by *S-performance* in a complementary manner (SOM_int_(S→O), t_23_ = 3.21; p = 0.0039; see Figure S4A for full details and summary in Figure 2C-ii). The two SOM effects persisted both when using estimates of S’s and O’s performance history that were not based on RL models and also in analyses that included the trials where subjects decided to avoid cooperation or competition (even though on such trials the relation to O might be less important, Figures S4B and S4C). Therefore, ability estimates for self and other were interdependent; SOM occurred both when judging oneself and the other individual.

### Neural Correlates of Appropriate Ability Estimation and Self-Other Mergence

In close correspondence to the behavioral analysis, we tested, first, whether there were brain regions encoding ability estimates for self and relevant other. Having identified such regions, we went on to test whether self and other signals were related to the appropriate attribution of past performance to the relevant player (*S-performance* to S and *O-performance* to O) or whether they indicated a misattribution between self and other. For all MRI analyses, we used a single GLM (see Supplemental Experimental Procedures 3.3 for details).

**Figure 3:**
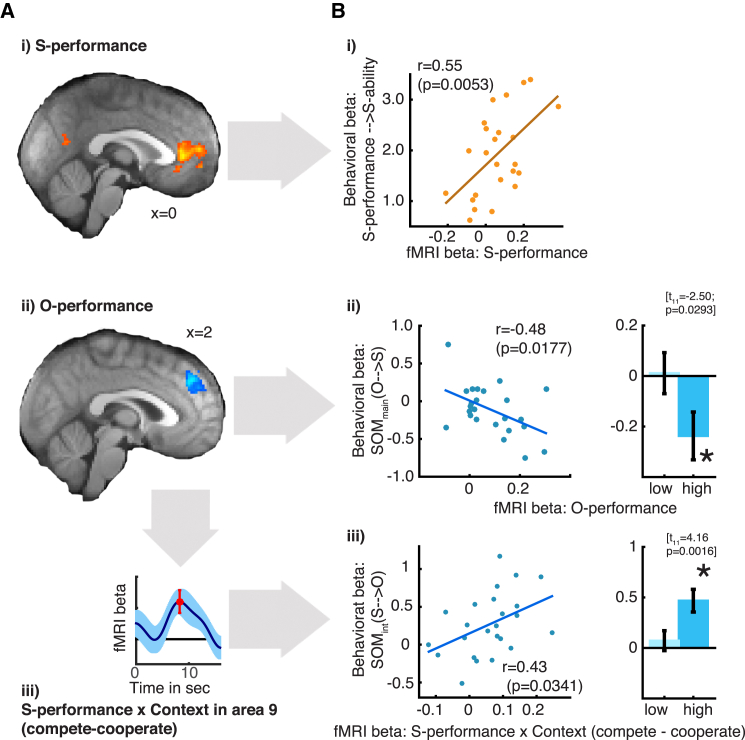
fMRI Results Whole-brain effects family-wise error cluster corrected, z > 2.5, p < 0.05. (A) Activation foci *for S-performance* in pgACC (i, yellow) and *O-performance* in area 9 (ii, blue). Area 9 showed more diverse social signals; a contextual *S-performance* signal was observed, which was stronger in competition than in cooperation (iii). The beta time course shows the context-dependent difference in *S-performance* (red line indicates signal group peak where beta weights were taken for correlations with behavior). (B) Relationship between neural and behavioral effects. (i) In pgACC, across-subjects variability in *S-performance* signal reflected the influence of *S-performance* on *S-ability*. (ii, left) By contrast, variation in area 9 *O-performance* signal was not related to the degree subjects based *O-ability* on *O-performance*; instead, it predicted the average influence of *O-performance* on *S-ability* (SOM_main_(O→S)). Note the behavioral variable plotted on the ordinate corresponds to the average effect (across cooperation and competition) of *O-performance* on *S-ability* (see Figure 2B-ii). (ii, right) In other words, only subjects with high *O-performance* signal showed a negative effect of *O-performance* on *S-ability* (median split; same y axis as on the left). (iii, left) The strength of the contextual *S-performance* effect in area 9 (A-iii) predicted the degree to which *O-ability* was influenced by *S-performance* in a context-dependent manner (SOM_int_(S→O) from Figure 2C-ii on y axis). (iii, right) Again, only subjects with higher *S-performance* interaction signals showed the corresponding behavioral effect of *S-performance* on *O-ability* (median split; same y axis as on the left) (error bars are mean ± SEM).

In order to identify regions tracking performance history, we focused on the decision phase and used a GLM very similar to the one used in the behavioral choice analysis (Figure S5; Table 1). This allowed us to distinguish between brain signals related to the self, the relevant other, and the variable threshold subjects had to exceed to win points on each trial (Figure S5A). Activity in perigenual anterior cingulate cortex (pgACC) reflected subjects’ own recent performance history (Figure 3A-i); it increased and decreased depending on how well subjects had themselves performed recently (and therefore how likely they are to perform well on the next trial). This *S-performance* signal in pgACC did not simply reflect individual choice values; it was present even after partialing out reward expected on a given trial (Figures S5B and S5C). Such an analysis was possible because the experimental design meant that on any trial reward expectation was only partly a function of *S-performance* (in addition it also depended on the threshold and *O-performance*, in different ways on cooperative and competitive trials).

In contrast, area 9 tracked *O-performance* history (Figure 3A-ii; Figures S5B and S5C), reflecting how well the relevant other is expected to perform in the minigames. Area 9 is part of the “theory of mind” network (Saxe, 2006) and is active during other-directed behavior (Sul et al., 2015). However, the effect of *O-performance* that we found in area 9 was a basic other performance tracking signal and as such is unlikely to reflect only emotional or motivational responses to the other player that vary as a function of context. As such, the *O-performance* signal could be distinguished from activity more generally related to O. For instance, area 9 and other nodes of the “theory of mind” network were also more active at the times when subjects rated the relevant other (Figure S5B-ii). Overall, the pattern of performance related signals was different in area 9 compared to pgACC (Figures S5C-ii and S5C-iii).

In all subsequent analyses, we focused on the same two regions of interest (ROIs) using the same GLM as used on the whole-brain level (although based on the same trials as in the behavioral analysis, Figures 2B-ii and 2C-ii) using time course analysis. To relate brain signals to behavior, we employed a two-step inference process considering only variables that were related specifically to past performance. First, we assessed whether an ROI carried a significant signal for a given regressor. Second, only if this was the case, we tested whether individual variation in the brain signal at the group peak was related to individual variation in behavior by correlating it with the respective behavioral beta weight from the self or other rating. We began by examining our main variables of interest: *S-performance* and *O-performance*.

We found that *S-performance* signal in pgACC predicted how much *S-performance* determined *S-ability* across subjects (Figure 3B-i; r = 0.55; p = 0.0053). Hence, pgACC signals reflected the use of information for estimating one’s own abilities, independent of others. We found no other performance related representations of self and other in pgACC.

In contrast to pgACC, the *O-performance* signal in area 9 did not predict how much ability estimates of O were guided by *O-performance* (r = 0.07; p = 0.74). Instead, it predicted the degree to which subjects’ *S-ability* decreased as a function of engaging with a strong other player (Figure 3B-ii; r = −0.48; p = 0.0177). This means that subjects with a stronger neural representation of the relevant other’s past performance in area 9 rated their own ability more negatively the better the relevant other performs. At a behavioral level, this corresponded to the tendency for *O-performance* to have a negative main effect on *S-ability* (the average effect of the two bars in Figures 2B-ii and S4A). The generally negative effect of *O-performance* on *S-ability* becomes apparent when considering only subjects with the strongest *O-performance* signals in area 9 (t_11_ = −2.5; p = 0.0293; Figure 3B-ii, right; median split of subjects). Therefore, area 9 activity reflected SOM in the estimation of the abilities of oneself.

To investigate whether area 9 was generally related to SOM, we examined whether such an effect was also present in the other direction: that past *S-performance* was used to estimate *O-ability*. In addition to *O-performance* signals, area 9 also carried an *S-performance* signal that was stronger in competition than in cooperation (interaction effect; t_23_ = 2.93; p = 0.008; Figure 3A-iii). This was consistent with our finding that area 9 was generally more active when there was a competitive relationship between S and O than when there was a cooperative relationship (main effect of context; Figure S5B-iii). We went on to test whether these self-related performance representations were also linked to SOM. Indeed, we found that *S-performance* influenced *O-ability* via area 9: the neural *S-performance* effect in area 9 (Figure 3A-iii) was predictive of the influence exerted by *S-performance* on *O-ability* shown in Figure 2C-ii (Figure 3B-iii; r = 0.43; p = 0.0341). In other words, the neural signature of *S-performance* in area 9 was different depending on context, and this difference predicted the context-dependent effect of *S-performance* on *O-ability* (SOM_int_(S→O)).

In sum, we found that, at a first approximation, self and other signals were found in distinct brain regions (pgACC and area 9), but further investigation revealed area 9 contained more diverse social signals relating also to oneself but within the social context. We found two SOM effects in area 9 that indicate that it is those subjects with stronger self and other related activity that are prone to SOM (Figures 3B-ii and 3B-iii, right panels, statistics embedded in figure). The findings were specific to area 9 (Figure S5C). Therefore, the stronger the representation of the other in area 9, the more the judgment of one’s own ability depended also on the other’s performance; analogously, the strength of area 9 self-related representations predicted how much they influenced the estimation of other’s abilities.

### Behavioral Adjustments Driven by Self-Other Mergence in Area 9

Finally, we investigated whether self-other mergence had an impact beyond the subjective estimates of abilities reported by subjects and tested whether its influence even propagated to behavior, and, if so, whether such an effect could also be related to area 9.

**Figure 4:**
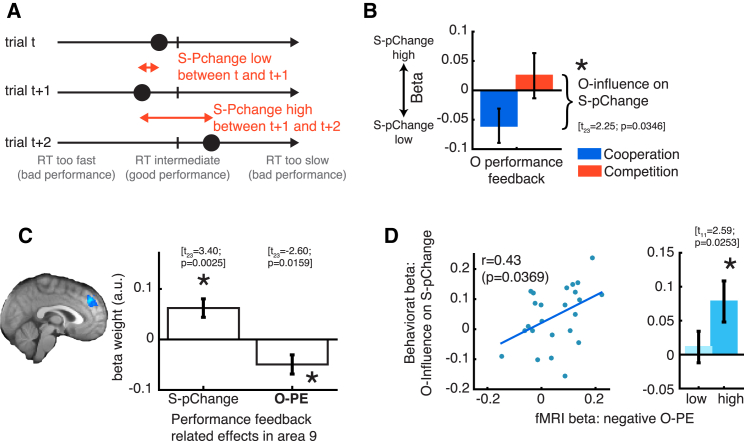
Self-Other Mergence in Minigame Performance Adjustments (A) Illustration of *S-pChange* reflecting the absolute (i.e., unsigned) difference in true minigame performance from one trial to the next of the same minigame. *S-pChange* constitutes an index of how much subjects changed their true minigame performance as a function of performance feedback. (B) Subjects’ performance adjustments were guided by their own performance feedback. *S-pChange* increased with more negative performance feedback (see main text). However, O performance feedback exerted an additional influence on *S-pChange*. It influenced *S-pChange* in a manner that paralleled its influence on *S-ability* (Figure 2; p value from Figure S6A). (C) BOLD activity in area 9 in the feedback phase of trials was related to the magnitude of *S-pChange* that would ensue from the current to the next trial (main effect). It also scaled negatively with a prediction error for O (O-PE). ROI taken from Figure 3A-ii. (D) Subjects with stronger (more negative) O-PE in area 9 were more influenced by O performance feedback in their subsequent minigame performance (left). As in the earlier results, SOM occurred only in subjects with stronger area 9 signal (median split; same y axis as on the left) (error bars are mean ± SEM).

We used the trial wise change in subjects’ performance, regardless of direction (*S-pChange*; Figure 4A) as an index of feedback driven behavior strategy adjustment from one trial involving a given minigame to the next trial with the same minigame. Note that *S-pChange* refers to the change in true performance, whereas *S-performance* and *O-performance* summarize the performance feedback observed. Subjects changed their performance in the minigames (for better or worse) more after negative performance feedback for S (t_23_ = 2.34, p = 0.0281). However, they also changed their performance more after negative feedback for O in cooperative trials, while in competition they changed their performance more after more positive feedback for O (*O-influence* on *S-pChange*; t_23_ = 2.25, p = 0.0346; see Figure S6 for full details and summary in Figure 4B). This means that the performance feedback, displayed for the relevant other after a minigame, had an impact on how subjects performed the minigame themselves when they encountered it the next time. Therefore, subjects used the performance feedback pertaining to the other to adjust their own behavior.

We tested whether this behavioral SOM effect was again mediated by area 9 using an ROI analysis (coordinates taken from previous analysis). This final part of the analyses was specifically intended as a replication of previous SOM effects. We used the same GLM as before, focusing on the feedback phase this time (Figure S5D). Again, we first aimed to identify activity related to the relevant other’s performance in area 9 and only subsequently to relate such activity to how much subjects were biased by the other player. First, we tested whether area 9 could play a role in preparing behavioral adjustments from trial to trial. We found that, indeed, in the feedback phase of the trial, activity in area 9 was correlated with the extent that subjects changed their performance from the current trial to the next trial (main effect *S-pChange*; Figure 4C). Second, at the same time, we found a negative signal scaling with the size of the (signed) prediction error for the relevant other (O-PE; Figure 4C). O-PE indexed how much the observed performance feedback for O deviated from subjects’ expectation of O’s performance level (Suzuki et al., 2012). Third, variation in O-PE signal predicted variation in *O-influence* on *S-pChange* (Figure 4D; r = 0.43, p = 0.0369). This replicates our previous findings linking area 9 to self-other mergence: the strength of other-related performance signals in area 9 predicted how much subjects’ adjusted their own performance as a function of the other player’s performance feedback.

## Discussion

People learn about themselves from objective experience (Bandura, 1977), but their judgments are also deeply influenced by the social environment (Allport, 1924, Festinger, 1954). In our experiment, we show that both mechanisms occur when learning about the abilities of self and others. Subjects form estimates of how well they themselves and others do based on explicit performance feedback. However, these estimates are not separate but influence each other reciprocally. Subjects systematically overestimated their abilities when cooperating with a good partner compared to a bad one, and the reverse was true in competition. Also, others were estimated as more similar in ability to oneself in cooperation but dissimilar to oneself in competition. Such interdependence could be described as “anchoring” (Tversky and Kahneman, 1974) self and other judgments to each other in cooperation but as the reverse in competition. Two adjacent areas in medial prefrontal cortex, pgACC and area 9, track self and other performance, respectively. Area 9 moreover integrated multiple signals relating also to the self and the context, and it predicted the degree to which self and other abilities were estimated in an interdependent fashion.

PgACC signals tracked subjects’ own performance. This area was previously found when subjects judged whether a given trait related to themselves (Denny et al., 2012, Kelley et al., 2002) and when mentalizing about oneself and similar other people (Mitchell et al., 2006) even when controlling for the effects of likeability (Mobbs et al., 2009). In our study, pgACC activity increased and decreased according to how well subjects thought they would perform in the experiment (*S-performance* signal; Figure 3). For subjects with a stronger *S-performance* signal in pgACC, performance history more strongly governed estimates of their own ability. Activity in other brain areas has been associated with action values, but our findings indicate that activity in pgACC reflects the assignment of ability, or a general value, to the self rather than the value of a particular choice (Figures S5B and S5C). Such representations in pgACC might be used to predict whether one is capable of succeeding in a given endeavor (Bandura, 1977) and as such might be subjectively perceived as rewarding in themselves. This idea is consistent with recent findings that regions in anterior cingulate cortex are related to the physical costs monkeys are willing to endure in order to obtain reward (Amemori and Graybiel, 2012). Moreover, such value assignments to the self (rather than value assignment to specific choices) may be altered in psychological syndromes such as depression (Murray et al., 2011). Note also that using predetermined and well-controlled performance feedback was critical for our investigation of feedback-guided ability learning as it decoupled feedback-guided learning about oneself from introspective (meta-cognitive) estimation of one’s own abilities (Bahrami et al., 2010, De Martino et al., 2013).

In contrast to pgACC, area 9 signals were more complex. There were clear signals relating to representation of the other. For example area 9 was active at the time point that subjects made ratings of the relevant other and more specifically it tracked other-related performance estimates and prediction errors (Figures 3 and 4). Activity in area 9 has been found frequently in previous studies when trait judgments were made about other people (Denny et al., 2012) and mentalizing about dissimilar others (Mitchell et al., 2006). In our study there were, however, also signals relating to self and the social relationship with the other. Individual differences in area 9 activity indicated how much self/other information was used in a relational way, predicting SOM both toward the self and toward the other.

**Figure 5:**
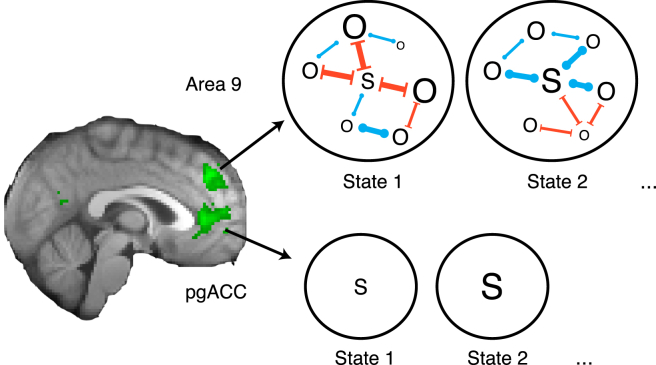
Self-Other Mergence and Interdependent Self and Other Representations in Area 9 In our study, pgACC holds a representation of oneself that is related to observing one’s own performance (Figure 3). It may reflect the value of one’s own actions for earning future reward or overcoming challenges. This ability estimate may be lower or higher depending on the state the individual is in (indicated by smaller and bigger “S”). In contrast, signals in dorsomedial area 9 suggest a different function. Area 9 holds a representation of the self, too; however, it is embedded in the cooperative or competitive context (Figure 3A-iii). In addition, it integrates information about the ability of others (Figure 3A-ii) and the social relationship one has with them (Figure S5B-iii). A neural circuit that integrates such information might be useful to facilitate coordination between oneself and others and allow, for instance, the pursuit of shared goals. It might also be used to flexibly compute one’s status within one’s social network as circumstances change (blue and red connections indicate changing alliances and rivalries, varying in strength). This would enable humans and animals to assign values to self and others based on the dynamics of their social relationship—something that very vividly guides choice in the complex dominance hierarchies of monkeys. If this is the case, then self-other mergence might accompany these neural processes in area 9 (Figure 3) occurring as a byproduct of relational self and other representations held by this brain region.

This sheds light on the function area 9 may have in social cognition. Self-other mergence might reflect difficulties that arise from tracking and assigning both self- and other-related information to the appropriate agent. In this sense, the effects we report are reminiscent of failures of credit assignment to the appropriate choice during reward-guided learning (Chau et al., 2015, Thorndike, 1911, Thorndike, 1933, Walton et al., 2010). However, self-other mergence may also be a side-product of relational computations in area 9 (Figure 5). In our experiment, the cooperative and competitive contexts create a social relationship between self and other that subjects need to be aware of. This meant that expectations of success in a trial were not a result of one’s own anticipated performance alone, but they were also dependent on the predicted performance of another player as well. Taking into account the actions of oneself in concert with another person’s actions is not a trivial matter (Tomasello et al., 2005), and it has been argued that coordinating actions between self and other often relies on implicit agreements (Misyak et al., 2014). It might be that self-other mergence indexes the relational representations that are used in many social situations where outcomes are the consequence of joint actions (Seo et al., 2014), possibly even if oneself is only a passive observer. From this perspective, when cooperating with a weak partner, the reduced chances of achieving a shared goal as a team might be carried over to impact negatively on one’s judgment of oneself. Note, however, that the strength of the direction of influence of self-other mergence (self to other or other to self) may depend on the social situation and other constraints. Furthermore, area 9 may integrate self, other, and relational information to compute one’s own position in a social network when an individual’s status is a reflexion of its alliances (Schülke et al., 2010). In monkeys, area 9 is related to dominance (Neubert et al., 2015, Noonan et al., 2014, Sallet et al., 2011), and thus adoption of a place in a social hierarchy might reflect the operation of a relational mechanism that could be performed in area 9.

## Experimental Procedures

### Subjects

Twenty-six subjects participated in the experiment. Two were excluded from data analysis due to excessive motion (final sample: 24 subjects; nine female; aged 19–31). All provided informed consent. The study was approved by the ethics committee of the University of Oxford (MSD-IDREC-C1-2013-133). Subjects received £35 as a show-up fee and a bonus based on task performance (range: £4.50–£11.80).

### Experimental Design and Schedule

While lying in the MRI scanner, on each trial, subjects performed a minigame. They were led to believe that two other subjects played the same minigame simultaneously (Figure 1; Figures S1–S3). Each trial took place either in a cooperative or in a competitive social context. Subjects then made an engage/avoid decision. On some trials, the choice was between cooperating or refraining from cooperating, while in other trials the choice was between competing or refraining from competing. If subjects took the “avoid” choice, then that meant that they were simply awarded a small number of points (1.5 points) randomly distributed around zero (and they were informed that this was the case). However, if they took the “engage” decision in the cooperative context, then they opted to ally themselves with one of the other players (which one varied from trial to trial and was indicated by the experimenter on each trial and is subsequently referred to as the relevant other, O) to see whether together they could perform well enough for their average points to exceed a threshold level (which varied from trial to trial). If they did, they gained reward points on that trial, but if they fell short of the threshold they lost points. By contrast, if they took the “engage” decision in the competitive context, then the other player became an opponent. The difference between the subject’s and opponent’s performances then had to exceed a threshold (again the threshold was variable), and the payoff was proportional to this difference (i.e., a win, a loss or neither of the two). In summary, the social context was critical when decisions to engage were made. Reward outcomes for engage/avoid choices were determined by minigame performances of S, O, and a threshold that varied unpredictably from trial to trial.

(Equation 1a) $$Engage\;Payoff_{Competition}=\left(feedback_{S}-feedback_{O}\right)-threshold$$

(Equation 1b) $$Engage\;Payoff_{Cooperation}=\left(feedback_{S}+feedback_{O}\right)/2-threshold$$

“Feedback” in Equations (Equation 1a), (Equation 1b) refers to performance feedback. The timing of events within each trial is illustrated in Figure 1.

While the likely performance feedback for S and O could be estimated from performance feedback on previous trials, the threshold varied unpredictably from trial to trial and was used to dissociate reward expectation from performance expectation and to make sure that subjects did not make their choices before the beginning of the current trial. Subjects found the meaning of the thresholds intuitive when the task was being explained to them, and their task behavior confirmed that they had understood the task.

Subjects then also provided an estimate of their ability on each trial by rating the expected performance for themselves (S) and the relevant other (O) for the upcoming trial of the minigame (Figure S2B). The order of S and O ratings was randomized across trials. As explained above, although both of the two other players performed the minigame simultaneously, subjects were only paired (to compete or cooperate) with one of the other players (the relevant other, O). Therefore, only O, and not the third player, was relevant for a trial’s engage/avoid decision. However, the identity of O switched between trials. On each trial, after the minigame, subjects received performance feedback about themselves as well as about the performances of the other two players (Figure S2A). As minigames, we designed two short reaction-time-based tasks. See Figure S3 for details on the minigames. The goal of the subjects in the experiment was to collect as many rewards (points) as possible, as these were translated into monetary reward at the end of the experiment.

For all three players, including the subjects themselves, performance feedback on every trial was predefined (Figure S1A). In other words, the feedback about performance was independent from subjects’ actual performance in the minigames (see, however, “false-start trials” for a case of veridical performance feedback in the “Feedback” section of the Experimental Procedures and Figure S2C). This was necessary to control and match performance feedback between subjects as well as between subjects and the two other players. Subjects were told that the minigames had been tested on a larger sample of subjects and that performance feedback in the minigame reflected individual performance relative to that sample.

In the phases before and after the minigames, three scales ranging from 1 to 15 points were shown with the initials of the three players below. Performance feedback was displayed on these scales in the feedback phase. While the initials of the confederates were the same for all experimental sessions, the subjects’ own initials were adjusted to be appropriate for each individual subject. The initials created a social frame for the experiment without using explicitly social cues such as faces.

The experimental schedule contained 136 minigame trials. The design was a 2(social context) × 2(partner) × 2(minigame) fully crossed design (17 trials per cell). This meant that a trial could be either cooperative or competitive (social context: cooperation or competition), the O could be either “player” 1 or 2 (O: Other1 or Other2), and, in each trial, subjects played one of two minigames (minigame: time task or color task). The trial type order was pseudorandom and the same for all subjects. Three subjects performed a marginally shortened version of the schedule. They did the first 116 trials of the schedule; however, the design was still fully crossed (14 trials per cell).

Several features of the experiment were counterbalanced independently across subjects to avoid confounds. The mapping between minigame (time task or color task), and associated performance feedback schedule was counterbalanced. Moreover, as the left/right sides of the buttons used to indicate the engage/avoid choice were fixed for each subject, they were counterbalanced across subjects. We also counterbalanced the screen location where initials of each confederate were displayed (left or right of subjects’ initials, which were always in the middle), and association between confederates (whom the subjects had met before going into the scanner) and their performance feedback schedules.

Thus, in summary, all trials comprised an engage/avoid decision, two binary ratings (for S and O), a minigame phase (described in detail in Figure S3), and a feedback phase. Timing details for all phases (except minigame phases) are shown in Figure S2.

### Experimental Procedures

The experimental procedure was precisely scripted and involved two experimenters, two confederates, and the radiographer to make subjects believe they would be playing an interactive game together with two other subjects. The same two confederates pretended to be the two other players for every subject. Details about the task instructions are presented in Figure S1B. The task in the MRI scanner took approximately 55 min. After functional and structural MRI sessions, subjects filled in two short questionnaires and were then fully debriefed about the experiment. No subject indicated any suspicion about false performance feedback or the identity of the confederates before debriefing. Supporting results from a debriefing questionnaire are shown in Figure S1C.

Note that we have several indications (and no counter-indications) that subjects’ ability estimates were guided by the performance feedback and therefore that subjects found the feedback credible: the effects of past performance feedback on (1) *S-ability* and *O-ability* ratings, (2) on decisions to engage in cooperation/competition (both Figure 2), (3) on true minigame performance (Figure 4), and (4) subjects’ self-reports of the feedback credibility in a debriefing interview, documented by a debriefing questionnaire (Figure S1).

### Ability Ratings

As already mentioned subjects provided S and O ability ratings. For each rating, initially, a tick indicated a value on the performance scale (rating marker) and subjects indicated whether expected performance (for S or O as appropriate) would surpass or fall below the rating marker (Figure S2B). A positive rating (i.e., performance is expected to be above the rating marker) was made with one button, and a negative rating (i.e., performance is expected to be below the rating marker) was made with the other button. As performance feedback was always expressed in integers, the rating makers were always set between two integers (X.5 values). The rating marker was updated from trial to trial based on the rating choices for the respective player using a staircasing procedure to increase sensitivity of the ratings. A positive rating resulted in an increase of the rating marker’s value by one point in the next trial of the same minigame for the given player; a negative rating resulted in a decrease by one point. The value of the rating marker on the first trial after the starter trials (see below) was based on the player’s mean performance feedback in the starter trials.

Subjects received a small payoff for the accuracy of the ratings. To reduce incentives to perform badly on the minigames, negative ratings never yielded payoffs. For positive ratings, subjects won or lost 0.25 points depending on whether the subsequent performance feedback received surpassed or fell below the rating marker. Note that the magnitude of the rating payoff was insignificant compared to the payoff for the engage/avoid decision.

### Feedback

Feedback was chunked together in three components that were presented in randomized order. The first component was the performance feedback for S and O, which was presented simultaneously with the information about the accuracy of the subjects’ ratings (Figure S2B). The second component was the payoff of the engage/avoid decision. For this, a cue indicating the trial’s choice appeared on the right side of the screen (Figure S2A) together with circles representing coins that were won (yellow circles with a plus sign) or lost (red circles with a minus sign). At the same time, only for engage choices, the performance feedback average (cooperation trials) or performance feedback difference (competition trials) was displayed on the scales on the right side of the screen. The third component was the performance feedback of the other player that was not the O (irrelevant other). The initials of this player were displayed in a different color, and the performance was irrelevant for any payoff. The three feedback components appeared in random order to control for sequence effects. The first component occurred after 1 s, the second component 1.25 s later, the third component another 1.25 s later, and the feedback phase ended after a further 4 s. Then, after 0.5- to 2.5-s inter-trial interval (ITI) with a blank screen, the next trial started.

Two types of trials deviated from the described structure. First, the first four trials of the experiment were “starter trials” (two with the time task, two with the in color task). Those trials were for subjects to form initial ability estimates about the players. For this reason, in starter trials, there was no option to cooperate or compete, and no ability ratings were made. Second, for trials where subjects performed very badly in a minigame (“false starts”), the feedback phase was adjusted. The performance thresholds for false-start trials are discussed in Figure S3. In false-start trials, subjects received no performance feedback for themselves, but only for the other players (Figure S2C; Figure S6B shows the number of false-start trials per subject). The sole payoff for false-start trials was a loss of two points independent of subjects’ ratings and engage/avoid choice. Subjects were instructed about this. It was explained that extremely bad performances would be detected by the computer running the experiment and discarded as false starts to sort out performance slips that were, for instance, due to inattentiveness and did not reflect a player’s “true” performance. This procedure was used to make the pre-determined feedback in other trials more believable as the feedback in false-start trials was actually determined by true minigame performance. Note that subjects were also told during the instructions that there would be a special type of false-start trial if one of the other players performed very badly. However, this never happened in the experiment. Starter trials and the feedback phase of false-start trials were excluded from fMRI analysis.

### Reinforcement Learning Model

See Supplemental Experimental Procedures 1 for details on the RL model.

#### Behavioral Analysis

See Supplemental Experimental Procedures 2 for details on behavioral data analysis.

#### Imaging Analyses

See Supplemental Experimental Procedures 3 and Figure S5 for details on MRI data acquisition and analysis.

## Author Contributions

M.K.W. and M.S.F.R. conceived the experiment. M.K.W., N.K., N.S.F., and M.S.F.R. designed the experiment. M.K.W. and N.N. collected the data. M.K.W. analyzed the data and wrote the manuscript. N.K., N.S.F., J.S., and M.S.F.R. provided expertise and feedback on data analysis and write-up; M.S.F.R. supervised the research.

## Figures and Tables

**Table 1:** Peak Coordinates of Significant Clusters in Whole-Brain fMRI Contrasts

Contrast	Region	Peak Coordinates x/y/z (in mm MNI Space)	Z Value
*S-performance*	Perigenual anterior cingulate cortex (pgACC)	0 40 6	3.98
Precuneus	−6 −64 18	3.38
*O-performance*	Brodmann area 9	2 44 36	3.43

Family-wise error cluster corrected, z > 2.5, p < 0.05.

## Supplemental Information

Document S1. Supplemental Experimental Procedures, Figures S1–S6, and Table S1

Document S2. Article plus Supplemental Information
