# Supplementary material for: Self-Other Mergence in the Frontal Cortex during Cooperation and Competition
Source: Neuron. 2016 Jul 20;91(2):482–93. doi: 10.1016/j.neuron.2016.06.022 (PMC4961240; doi:10.1016/j.neuron.2016.06.022)
Supplement: Document S1. Supplemental Experimental Procedures, Figures S1–S6, and Table S1 [file mmc1.pdf]

Neuron, Volume *91*

Supplemental Information

Self-Other Mergence in the Frontal Cortex

during Cooperation and Competition

Marco K. Wittmann, Nils Kolling, Nadira S. Faber, Jacqueline Scholl, Natalie Nelissen, and Matthew F.S. Rushworth

Supplemental data items:

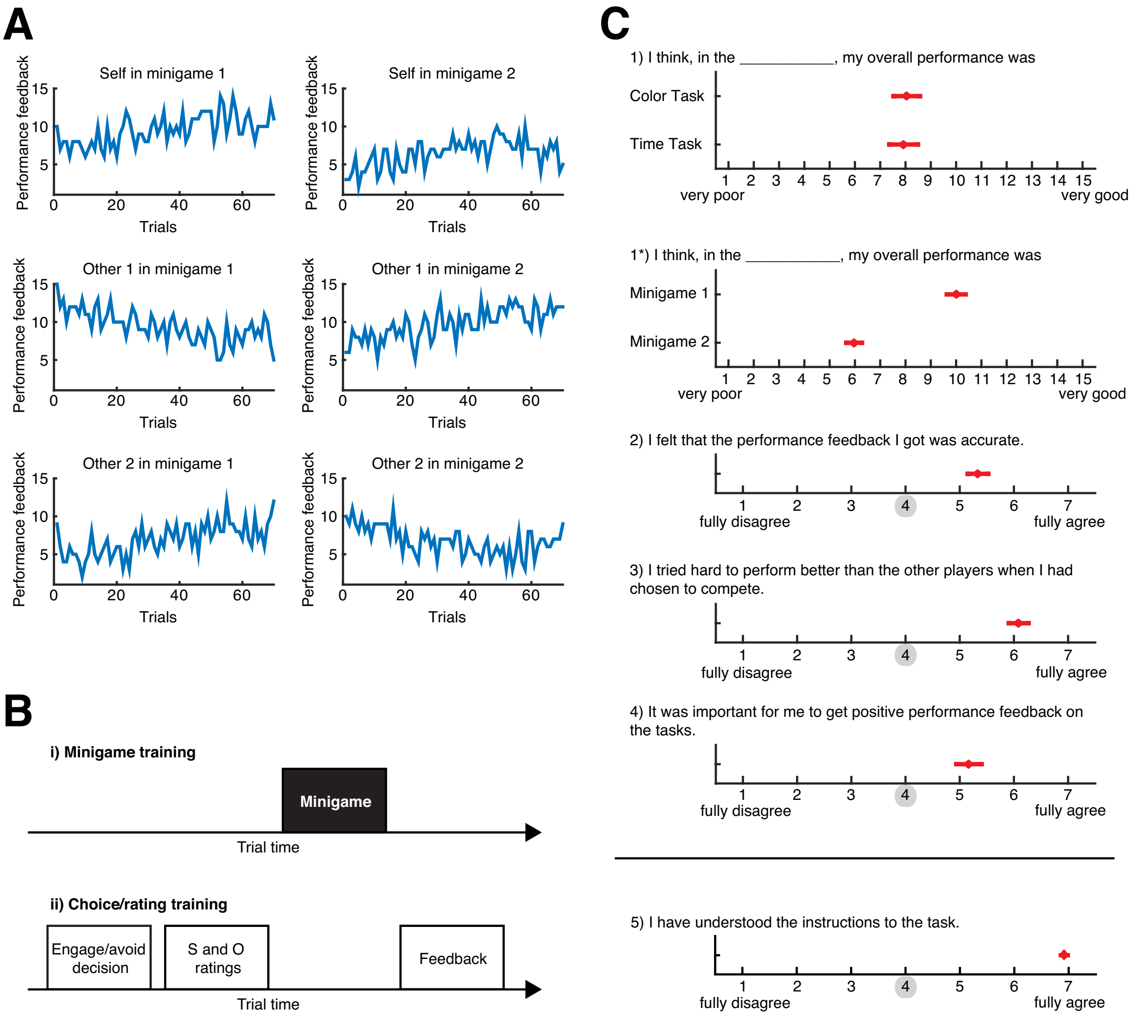

Figure S1 (relating to Figure 1)

Performance feedback schedules, instruction and debriefing questionnaire. (A) Performance feedback was predetermined to guarantee that self and relevant other (S and O, respectively) performance feedback allowed a meaningful data analysis. The performance feedback for S and O was similar in mean and variance. However, very bad minigame performance did lead to veridical performance feedback (see false start trials in Experimental Procedures). Subjects performed one of two minigames on each trial and S performance feedback was more positive in one minigame compared to the other minigame (counterbalanced across subjects), while variance in S performance feedback was similarly small in both minigames. This was done to sample a wide range of S performance feedback while still keeping S performance feedback relatively stable within each minigame. The same logic applied to the performance feedback schedules of the two other players. In short, mean, variance and slow drifts of performance feedback were balanced for self and others to ensure that differential behavioral and neural effects were not driven by schedule differences. The relevant other (O) in a trial was Other 1 in half of the trials and Other 2 in the other half. Trials with minigame 1 and minigame 2 were pseudorandomly interleaved. **(B)** The experimental procedure and instructions followed a precise schedule. Two confederates arrived in the laboratory at a similar time as the subjects. Subjects had been told in advance that the experiment investigates learning and decision making in social situations and that two other naïve subjects would participate in the same experiment. After a short introduction, subjects were separated from the confederates and instructed about the experimental task. They were told that they would be playing an interactive game together with the confederates, who would play the game from computers outside the scanner room. A faked lottery was

used to determine who of the three alleged subjects went in the scanner. For approximately 45 minutes, subjects were instructed on the minigames, the ratings and the engage/avoid decisions and performed example trials. The instructions were designed such that actual performance learning would only take place in the subsequent experiment to maximize learning effects in the scanner. To still guarantee familiarity with the minigames, a written explanation of the two minigames was complemented with a short practice session, in which subjects performed 5 trials of each minigame (B-i). During those trials, the experimenter was present and made sure that subjects understood the minigames. No explicit performance feedback was given on those trials to avoid performance learning. To guarantee that subjects understood the logic of the engage/avoid decision and the ratings, subjects performed 16 example trials that did not include minigames, but instead a placeholder screen (B-ii). This allowed subjects to adjust to the trial events and experience the reward outcomes of ratings and decisions. Importantly, the performance feedback on those trials followed no across-trial contingencies and consisted mostly of the highest or lowest performance feedback for the players. This was done to make these example trials very different from the trials experienced in the main experiment. Although subjects could not learn anything during those trials and therefore could make no well-grounded decisions, they were asked to invent and verbalize reasons for their ratings and decisions so that the experimenter could make sure that they understood their logic (e.g. "This is a cooperate trial. I press the "engage" button, because although the threshold is high, I think we will perform very well. I rate myself and the other one positively, because I think we will both perform well..."). In sum, subjects practiced all aspects of the experimental task, but in such a manner that they could not yet learn about their performance. Subjects were told that their goal in the experiment was to collect as many points as possible and that points would be translated into monetary reward at the end of the experiment. It was emphasized that points could be earned by making good decisions and by providing accurate ratings of performance. Despite the substantial time needed for a thorough instruction, most subjects found the task intuitive and both the behavioral data acquired in the experiment (see main text) and a post-experiment questionnaire confirmed that they understood the task. See question 5 in panel C: 22 subjects gave a 7 out of 7 rating for the question "I have understood the instructions to the task" (the remaining 2 subjects gave 6 out of 7). **(C)** After the experiment, subjects filled in a debriefing questionnaire with several questions that they rated on scales which were anchored for highest and lowest performance scores (see figure). These data showed that across the whole sample, subjects did not rate their overall performance in the two minigames differently (item 1; paired t-test: t_{23}=0.13; p=0.9;). This was expected as we counterbalanced across subjects whether time task or color task was used as minigame 1 which had higher performance feedback on average compared to minigame 2 (see upper left panel in (A); for 11 of 24 subjects the time task was used as minigame 1). Note also that there was no confound in the ordering of minigames because trials of minigame 1 and minigame 2 were interleaved across the whole experiment. When we retrospectively recoded item 1 with respect to minigame 1 and minigame 2, we found that subjects rated themselves better in the minigame where they received better performance feedback (item 1*; t_{23}=7.72; $p=7.7*10^{-8}$). Note that the performance scores for item 1 and 1* are very similar to the actual average performance feedback (see panel (A)) indicating further that subjects learned from the performance feedback. To confirm the believability of the performance feedback, we asked subject whether they felt that the performance feedback was accurate. If subjects experienced a mismatch between their subjective experience and the performance feedback they received, this would have been indicated by “disagree” ratings. We found slight agreement, however, indicating that subjects were unable to tell the inaccuracy of the performance feedback from their subjective experience. Additional suspicion checks asked whether they tried to perform better than O in competition (indicating that they believed that they could gain positive performance feedback by trying hard; item 3) and if positive performance feedback was valuable for them (item 4). In all cases (items 2,3,4) subjects tended to agree rather than disagree (average scores higher than scale point 4). This suggests that subjects indeed experienced the performance feedback as a consequence of their actions in the minigames. Note, importantly, that in addition to the debriefing questionnaire, a debriefing interview took place for every subject with additional suspicion checks. As we handed out this questionnaire before debriefing, to avoid biased responses, we could only indirectly ask if subjects had suspicions about the deception used. Therefore, we conducted debriefing interviews to fully disclose the purpose and method of the experiment to subjects, but also to confirm that all subjects, without exception, indicated that they believed the false performance feedback and had no doubts about the identity of the confederates. (error bars are mean +/-SEM across subjects).

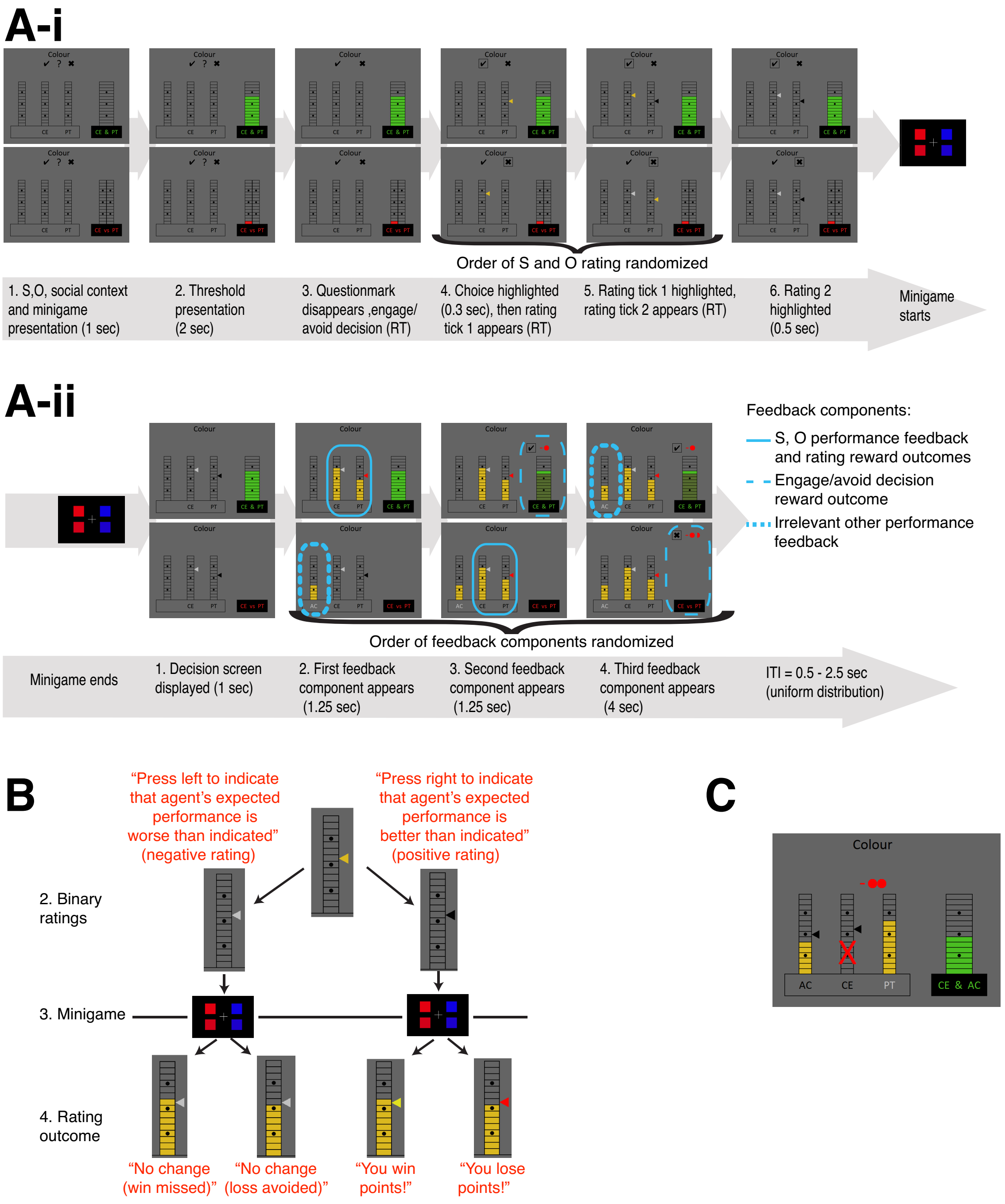

Figure S2 (relating to Figure 1)

Trial structure. (A) For an example trial, we show the first part of the trial in which the social context (competition or cooperation) was revealed, participants made the engage/avoid decision, and provided self (S) and relevant other (O) ratings (i). The feedback phase is shown of the same example trial (in ii). For illustration purposes, the same performance feedback is shown in a cooperative context (upper rows) and a competitive context (lower rows). Note that timing of the events are shown in brackets and RT (reaction time) means that the respective step only ends when the subject makes a response. **(A-i)** At the beginning of a trial S (initials of the subject), O (initials of one of the two other players relevant for the current trial), social context (cooperation or competition) and type of upcoming minigame ("Colour" or "Time") is presented. The initials of the irrelevant other player are not shown and the social context is color-coded indicating also the threshold of the current trial. In these examples, the choices made (see highlighted tick and cross in step 4) are to engage in cooperation (indicating the expectation that S and O will, on average, perform better than the threshold of 10) and to avoid competition (indicating that the subject does not expect to perform at least one point better than the O).

After the engage/avoid choices, binary ratings of S and O take place (step 4 to 6) in randomized order (in these examples, O rating first in cooperation and S rating first in competition). The ratings shown here indicate that the subject expects himself or herself to perform worse than 10.5, while the O is expected to perform better than 8.5 (see panel (B) for color coding of rating ticks). Note that these ratings are consistent with the engage/avoid choices shown in the two examples. **(A-ii)** In the feedback phase, the previous screen from before the minigame reappears. However, the right side of the screen showing the cooperation/competition threshold is occluded if a subject had chosen to avoid cooperation/competition in the previous decision phase of the trial. This means that in this example trial the threshold is only shown again in the cooperate trial (because the subject actually decided to cooperate) and not in the compete trial (because in this example trial the subject refrained from competing). In other words, the repeated presentation of the threshold is not a feature of the social context, but entirely a consequence of the engage/avoid choice made. Subsequently, three feedback components appear in randomized order to control for sequence effects (see legend on the right hand side). In cooperation, the choice payoff is -1, because the average performance is 9 while the threshold is 10. In competition, the choice payoff is -1.5 which is due to chance (payoff from avoid choices is +1.5 or -1.5 with a 50/50 probability) and independent of performance feedback. Note that the subject would have earned a payoff of 3 had the engage choice been taken (performance feedback difference of +4 minus threshold of +1). See panel (B) for an explanation of the rating payoff. Note that, overall, the magnitude of the rating payoff is marginal compared to the engage/avoid choice payoff. **(B)** Rationale of binary ratings. Before the minigame, subjects indicated for S and O either a positive or a negative rating reflecting the expectation that the player would surpass or fall below a given rating marker. The color change of the rating marker was indicative of the choice made. The rating marker turned black for a positive rating and grey for a negative rating. A positive rating led to a win or loss of 0.25 points depending on subsequent performance feedback. A negative rating led to no change in the points count independent of performance feedback. Therefore, making a correct negative rating was associated with a benefit of avoiding losing points while making a correct positive rating was associated with a benefit of winning 0.25 points. Note that in panel (A-ii), the performance and rating feedback indicate an incorrect negative rating (missed win) for S and an incorrect positive rating (loss) for O. Red text in quotes is taken from the subjects' instructions where a similar illustration was used. **(C)** The screenshot shows the feedback screen of a false start trial. In false start trials, the true performance of the subject in the minigame was below a predetermined threshold for acceptable performances. False start trials were a case of veridical performance feedback to ensure that performance feedback in general was believable. The feedback phase in false start trials was not analyzed and no prediction error for S was calculated on those trials (reward prediction error and prediction error for others were calculated as normal). Subjects were instructed about false start trials.

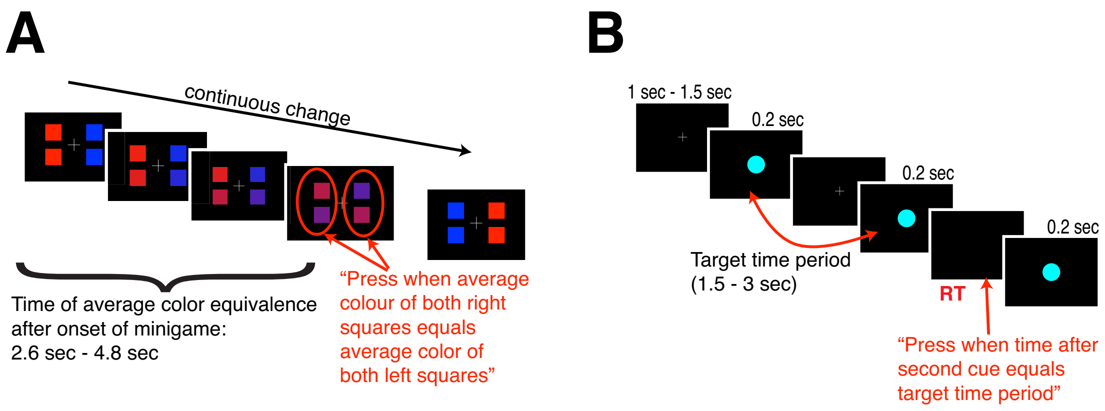

Figure S3 (relating to Figure 1)

Minigame description. We used two minigames in the experiment labeled "time task" and "colour task". The minigames were necessary prerequisites to administer performance feedback on every trial. They were short reaction-time based tasks and we designed them to be relatively non-transparent in the sense that the subjective experience of performing the minigames was not very informative for estimating one's ability compared to the explicit performance feedback that was given to the subjects. Also, we varied the timing parameters of the minigames to make it harder to compare true performance across different trials of a minigame. Lastly, we designed both minigames so that they appeared to be measuring different skills to make it plausible for subjects that performance levels in one task were not predictive of performance level in the other task. Each minigame was performed with one response button press with the right index finger. **(A)** The color task is loosely based on perceptual decision making tasks (Michael et al., 2014). Two pairs of squares, one pair red on the left, one pair blue on the right of a fixation cross, initially appear on screen. Then, the squares gradually change color over several seconds until the pairs have reversed their color. The color change occurs at an uneven rate. Subjects were asked to press a button when both pairs of squares had the same average color. The average colors were defined in RGB space as:

$$Color_{left} = [c_{red} \quad 0 \quad 1 - c_{red}]$$

$$Color_{right} = [c_{blue} \quad 0 \quad 1 - c_{blue}]$$

The colors were controlled by two parameters c_{red} and c_{blue}. For the left, initially red ([1 0 0] in RGB space) side, the parameter c_{red} decreased linearly from 1 to 0 over a run of the minigame. Vice versa, for the right, initially blue side ([0 0 1] in RGB space), the parameter c_{blue} increased linearly from 0 to 1 over a run of the minigame. The optimal time to press was when c_{red} and c_{blue} had the same value (color equivalence point), which happened only once as both parameters changed in opposing directions. The time of color equivalence was set to occur at a randomly picked time point drawn from a uniform distribution between 2.5 seconds and 4.7 seconds after onset of the color change. The c-values at that time were set to have a random value ranging from 0.25 to 0.75, also drawn from a uniform distribution. This meant the color equivalence point could lie rather towards the pure red or the pure blue side (a value of 0.5 indicates an even mix). Note that c_{red} and c_{blue} characterize the average color of the two left and two right squares, respectively. Hence, c_{red} (and c_{blue} analogously) was subdivided in c_{red1} and c_{red2} by multiplying c_{red} with a scaling parameter "colscale", which was randomly picked for each run of the minigame and ranged between 0.5 and 1.5 ($c_{red1} = c_{red}$ x colscale; $c_{red2} = c_{red}$ x (2-colscale)). In other words, c_{red} subdivided so that it was always the average color of c_{red1} and c_{red2}. Performance on this task was calculated as:

$$Performance_{ColorTask} = (c_{blue} - c_{red}) \times 100$$

Optimal performance was represented by a performance value of 0 indicating a color match and the performance values represented deviation from color equivalence in percent. Negative performance values indicate a response that was too quick (the point of equal c values had not yet been reached

when the subject responded) and positive performance values indicated a response that was too slow (c values are past the point of equality). If absolute performance values exceeded 60, trials were classified as false starts. This threshold was based on pilot experiments. Note again, that except for false start trials, the performance feedback was unrelated to the performance measure explained here. **(B)** In the time task subjects had to replicate a given time gap between two short occurrences of a blue dot on the screen (target time). The target time was randomly picked for each trial from a uniform distribution and ranged between 1.5 and 3 seconds. Subjects were instructed to press when the time elapsed after the second dot appearance was equal to the target time. The response time was the time from the second dot appearance to the button press. Therefore, performance on the task was calculated as:

$$Performance_{TimeTask} = (response\ time / target\ time) \times 100 - 100$$

As in the color task, optimal performance on the time task was represented by a performance value of 0 and the performance values represented deviation from the optimal response time in percent. Positive performance values indicated a response that was too slow and negative performance values indicated a response that was too quick. If absolute performance values exceeded 70, trials were classified as false starts. This threshold was based on pilot experiments.
Red text in (A) and (B) is taken from the subjects' instructions where a similar illustration was used. (RT, reaction time).

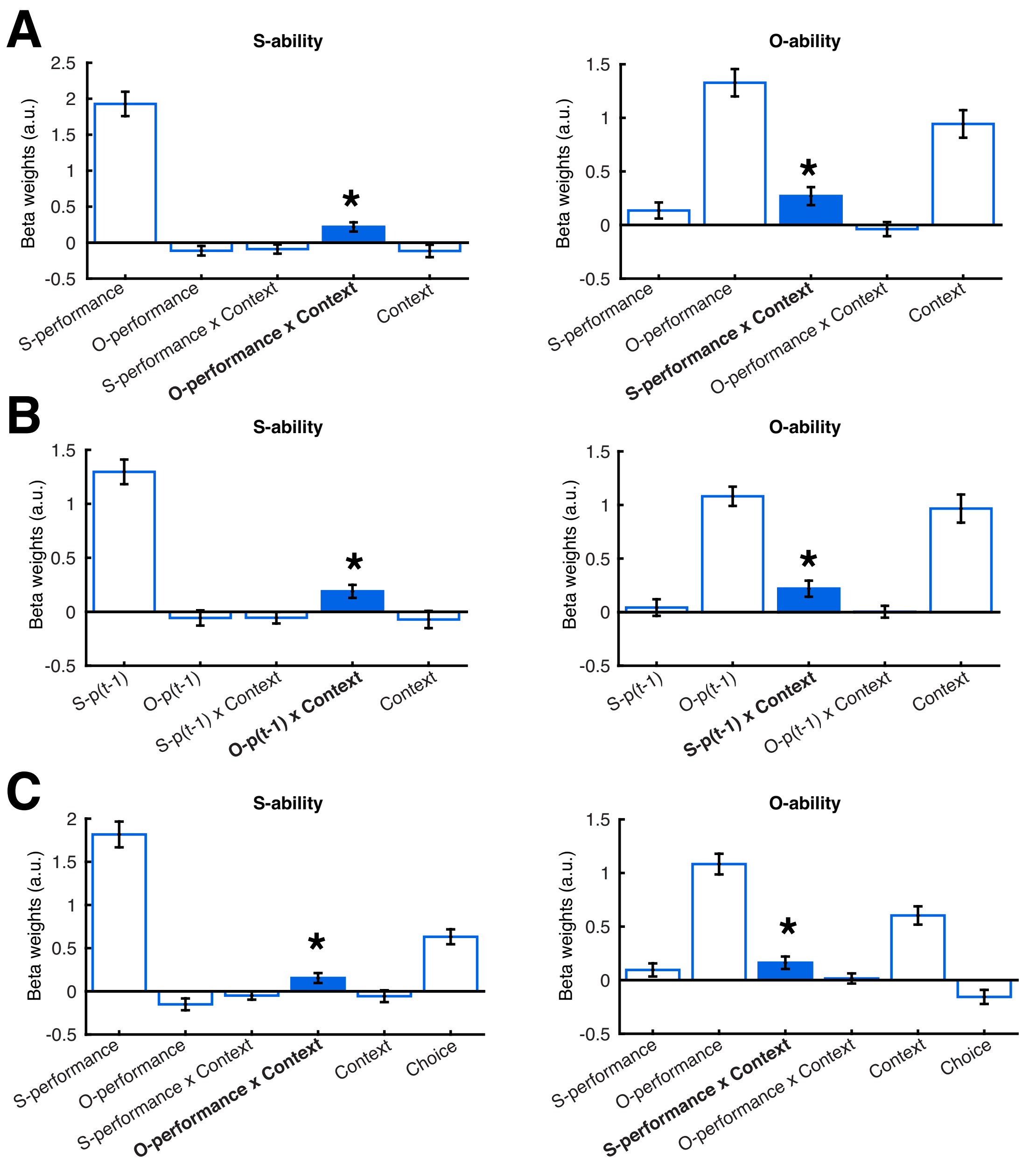

Figure S4 (relating to Figure 2)

Behavioral GLMs on the self ratings (*S-ability*, left panels) and relevant other ratings (*O-ability*, right panels) for engage trials (panels A and B) and all trials (C). For clarity, filled blue bars highlight self-other-mergence interaction (SOM_{int}) effects. We calculated the GLMs shown in Figure 2B-ii,C-ii (rating GLM2a, Supplemental Experimental Procedures 2) separately over compete and cooperate trials. For these GLMs here (rating GLM2b, Supplemental Experimental Procedures 2), however, the difference in effects of the observed performance history between cooperate and compete trials was coded as an interaction effect (*S-performance x Context* and *O-performance x Context*, respectively) such that a positive interaction reflects stronger influence of *S-performance/O-performance* for cooperate compared to compete trials. **(A)** We found significant SOM_{int} for *S-ability* (t-test against zero; *O-performance x Context* effect: t_{23}=3.39; p=0.0025) and O-ability (t-test against zero; *S-performance x Context*: t_{23}=3.21; p=0.0039). The results of these two significance tests are shown in the main text in Figure 2B-ii and Figure 2C-ii, respectively. In other words, we used the GLMs shown in panel (A) for significance testing of the *S-performance* and *O-performance* effects binned by social context that are shown in Figure 2B-ii and Figure 2C-ii. Similarly, the beta weights of the relevant effects in panel (A) were correlated with brain signals across subjects in Figure 3B-i,ii,iii. In detail, this was the behavioral effect of *S-performance* on *S-ability* for Figure 3B-i, the effect of *O-performance* on *S-ability* for Figure 3B-ii (also referred to as SOM_{main}(O-->S), because it is the main

effect of the relevant other's performance independent of social context) and the effect of *S-performance x Context* on *O-ability* for Figure 3B-iii. There is also a main effect of Context on *O-ability*, suggesting that the other is evaluated better in cooperation than in competition, mirroring research demonstrating a general evaluation bias in favour of cooperating in-group members compared to competing out-group members (Brewer, 1979). **(B)** Control GLMs for results presented in panel (A) (which used rating GLM2b) that do not use parameters derived from a reinforcement learning (RL; Supplemental Experimental Procedures 1) model. These GLMs used the same regressors as in panel (A), except that the summary terms for performance history, *S-performance* and *O-performance*, were replaced with the most recent piece of performance feedback that was received for that player (S-p(t-1) and O-p(t-1), respectively). As in panel (A), this control GLM is calculated using engage trials only. Interaction effects with social context were calculated as in panel (A) resulting in significantly positive SOM_{int} effects for *S-ability* (*O-p(t-1) x Context*; t_{23}=3.13; p=0.0047) and *O-ability* (*S-p(t-1) x Context*; t_{23}=2.92; p=0.0078). This demonstrates that self-other-mergence effects for both *S-ability* and *O-ability* did not rely on specific parameters used by our RL model for summarizing performance history. **(C)** Control GLMs for results in panel (A) (which used rating GLM2b) showing significant SOM_{int} when the analysis is not restricted to engage trials (as in panels (A) and (B)) but also includes trials on which subjects avoided cooperation/competition. We repeated rating GLM2b but extended the analysis over both engage and avoid choice trials. Including avoid choices is problematic because O performance is irrelevant to the participants' outcomes on these trials and so any SOM effects are expected to be weaker. To account for choice, we added a binary choice regressor (normalized to a mean of zero and standard deviation of 1, positive values for engage, negative values for avoid trials). The SOM on *S-ability* (t_{23}=2.67; p=0.0138) and *O-ability* (t_{23}=2.79; p=0.0103) still remained significant. Note that, among the points raised in the main text, this inability to fully distinguish own competence from the competence of others might also help understanding why people who cooperate in a group often are not able to make good use of the individual knowledge they could contribute (Stasser and Titus, 1985) and, in general, why performance in groups is so hard to predict from individual performance (Faber et al., 2015). (error bars are mean +/-SEM; *, $p < 0.05$)

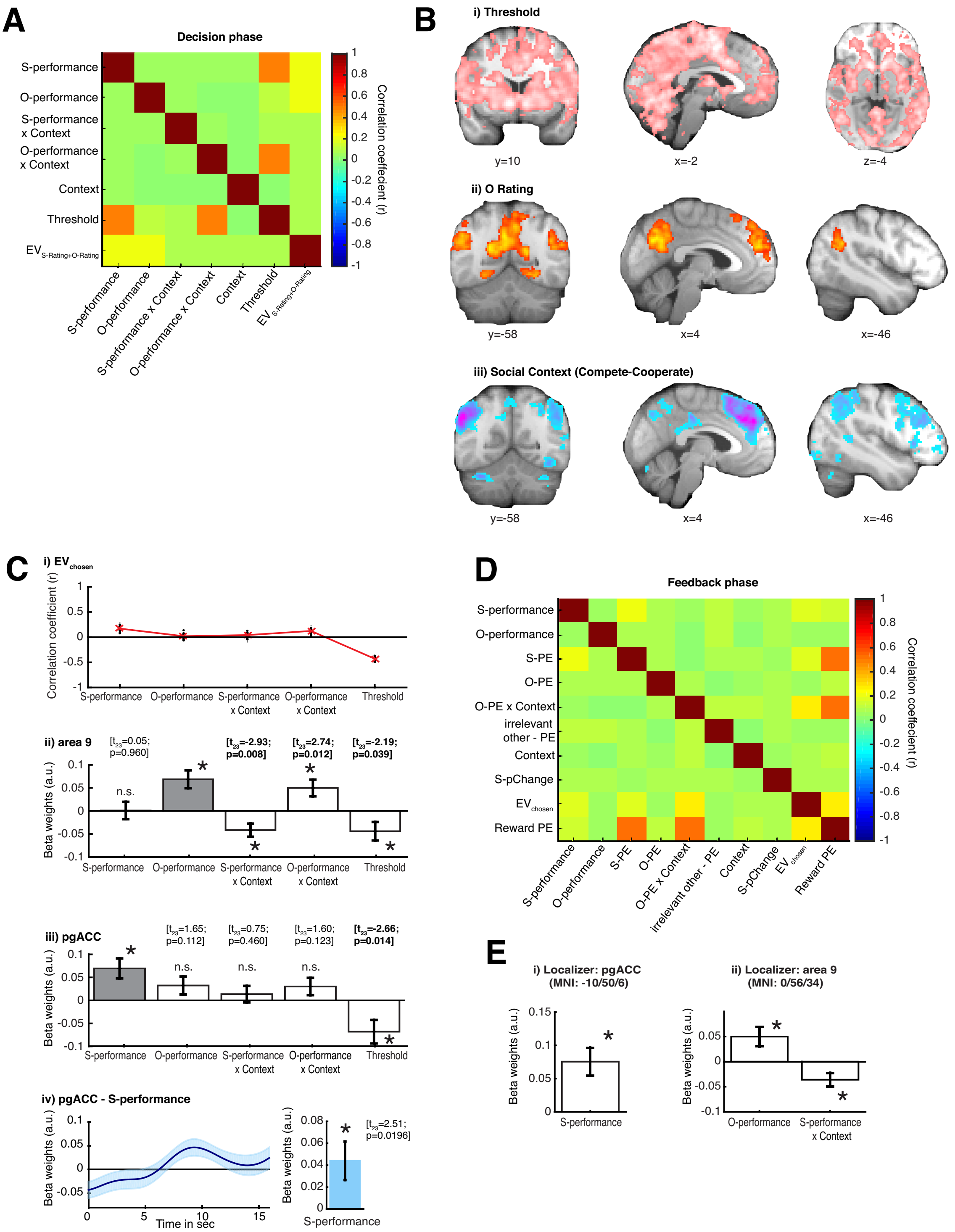

Figure S5 (relating to Figure 3)

Supplemental fMRI results. (A) Correlation matrix of regressors of interest at the time of the decision over all trials. **(B-i)** Cluster-corrected negative effects of threshold at the time of the decision. The threshold had widespread negative effects, which in medial prefrontal cortex centered on the ventromedial prefrontal cortex (vmPFC) and not pgACC or area 9; the threshold-related activity can be considered as activity related to the decrement in reward expectation on the current trial caused by the presence of the threshold (in other words the cost of the current specific choice) rather than to *S-performance* or *O-performance*. **(B-ii,iii)** At the time when the rating of the relevant other was made, we found cluster-corrected positive effect (main effect of O rating, as opposed to a parametric contrast) in several brain regions typically associated with "theory of mind"-type aspects of social cognition (Saxe, 2006). These regions include dorsomedial prefrontal cortex, bilateral temporoparietal junction

and precuneus (ii). The effect of the social context (compete-cooperate) at the time of the decision had widespread cluster-corrected positive effects (iii). The regions include again dorsomedial prefrontal cortex and bilateral temporoparietal junction. Although the activation was widespread, the peak of activation in dorsomedial prefrontal cortex was, just as in the case of the "theory of mind" activations presented in ii, in area 9 as identified by *O-performance*. (**C-i),** Single subjects (small black dots) and median correlations (red line) between regressors of interest (from panel (A)) and EV_{chosen} (as defined in equation 10, Supplemental Experimental Procedures 1). We designed the experiment so that the correlations between EV_{chosen} and the expected performance of the players (as well as the corresponding interactions by social context) were as small as possible. However expected reward is still linked conceptually to *S-performance*, *O-performance x Context* and in particular the threshold, as the reward outcome of the engage/avoid decision was a linear combination of performance feedback S, performance feedback O (positive relation in cooperation, negative relation in competition) and the threshold. **(C-ii,iii)** Effects of regressors of interest for area 9 and pgACC. Grey bars indicate contrasts used to identify the ROI. The significance stars on these effects denote the statistical significance from analyses conducted at the whole-brain level; these effects were not again tested for significance in the ROI (to avoid "double dipping"). Note that the interaction term with social context is coded in a way that positive effects mean stronger signals for cooperate compared to compete trials (interaction uses "1" for cooperation, "-1" for competition). In Figure 3 of the main manuscript, the interaction term *S-performance x Context* is coded the other way around in the fMRI analysis shown, so that positive signals indicate stronger responses in compete compared to cooperate trials. This was done for visualization purposes only. We used a repeated measures analysis of variance to formally examine whether performance related brain signals were indeed different in pgACC compared to area 9 (Nieuwenhuis et al., 2011). For this, we first used leave-one-out procedures on the whole brain level to make sure that we performed this analysis in an unbiased way. With respect to the pgACC ROI, we identified the peak voxel for *S-performance* effects based on the whole group of subjects except one left out subject. We repeated this for all subjects. Thus, we were able to conduct ROI analyses without selection biases, as each subject's brain signals did not contribute to the ROI that was selected for that subject. In the same way, we identified the area 9 ROI using *O-performance*. We used a 2 [brain region] x 4 [performance signal] repeated measures ANOVA to compare the performance-related brain signals in both areas. The ANOVA included all four agent-specific signals that we investigated at the time of the decision (*S-performance, O-performance, S-performance x Context, O-performance x Context*) and compared their neural effect sizes for area 9 and pgACC. As expected, we found a highly significant interaction effect ($F_{3,69}$=6.7; p=0.0005). Main effects were absent (both p>0.08). **(C-iv)** Analysis of *S-performance* activity in pgACC in relation to EV_{chosen}. As *S-performance* is conceptually related EV_{chosen} (better *S-performance* leads to higher payoff in cooperation and competition) compared to *O-performance* (better *O-performance* leads to higher payoff in cooperation but to lower payoff in competition), we went on to analyze more formally if *S-performance* signals in pgACC could be reduced to EV_{chosen}.

First, we used again a leave-one-out procedure to determine individual pgACC ROIs using *S-performance* (see C-ii,iii).Within these ROIs, we conducted a time course analysis (Supplemental Experimental Procedures, section 3.3) using EV_{chosen} as the only regressor. We reapplied our original GLM (fMRI GLM for decision phase) to the residuals of this time course. In other words, we conducted our GLM of interest on a timecourse where the effects of EV_{chosen} had been partialled out. The effect of *S-performance* in this GLM is shown in panel (C-iv) as a time course analysis (left) and as a bar chart analysis (t_{23}=2.51; p=0.0196; right, Supplemental Experimental Procedures section 3.3). In sum, *S-performance* effects in pgACC persist after partialling out EV_{chosen} and therefore cannot be explained by the expected reward of the choice alone.

Note that the relationships between brain signals in pgACC and area 9 and behavior shown in Figure 3B of the main manuscript were specific to the respective brain area. This meant that any *S-performance* signal in area 9, regardless of its significance (in fact such signals were also non-significant), did not correlate with the effect of S-*performance* on *S-ability* (r=-0.27; p=0.20). Similarly, the neural signals that correlated with behavior in area 9, when measured in pgACC (again regardless of their significance), did not correlate with the behavioral variables shown in Figure 3B-ii,iii. In detail, any *O-performance* signal in pgACC did not predict the influence of *O-performance* on *S-ability* (r=-0.24; p=0.26) and *S-performance x Context* did not predict SOM_{int}(S-->O) (r=-0.02; p=0.91). Note that panels (ii) and (iii) show that none of these three signals was significant in the other area over all trials. Moreover, the correlation shown for pgACC was specific for the "rational" influence of *S-performance* on *S-ability* and did not occur with the "irrational" effect of *S-performance* on *O-ability* (r=0.03; p=0.88). Analogously, the behavioral correlation associated with area 9's *O-performance* signal was specific to the case of self-other-mergence (correlation of brain signal with *O-*

performance effect on *O-ability*: r=0.07; p=0.74) and so was the area 9 *S-performance x Context* effect specific to the case of self-other-mergence (correlation of brain signal with *S-performance x Context* effect on *S-ability:* r=-0.04; p=0.85).

(D) Correlation matrix of regressors of interest at the time of feedback (Supplemental Experimental Procedures, section 3.3, Figure 4C) over all trials. **(E)** Supplemental control analysis for self and other related signals in pgACC and area 9. We repeated our ROI analysis (similar to panel C-i,ii in this figure) for two independent ROIs. A recent meta-analysis of fMRI studies investigated trait judgments about self and others (Denny et al., 2012) and identified brain regions in medial frontal cortex where self-related judgments show increased BOLD activity compared to other-related judgments (self>other) and vice versa (self<other). The two regions correspond to our regions pgACC (i) and area 9 (ii), respectively. We replicated our effects of interest shown in Figure 3A for ROIs centered on the peak coordinates of these clusters. In detail, we again found a significant effect of *S-performance* in pgACC (t_{23}=3.61, p=0.002), and effects of *O-performance* (t_{23}=2.56; p=0.018) and *S-performance x Context* (t_{23}=-2.67; p=0.014) in area 9. Therefore, our results are not dependent on using a specific ROI location and they link up with the results of previous studies investigating similar concepts. Note that, as in panel C, the *S-performance x Context* effect is sign flipped compared to Figure 3 for ease of visualization in the main text. (error bars are mean +/-SEM; *, $p < 0.05$)

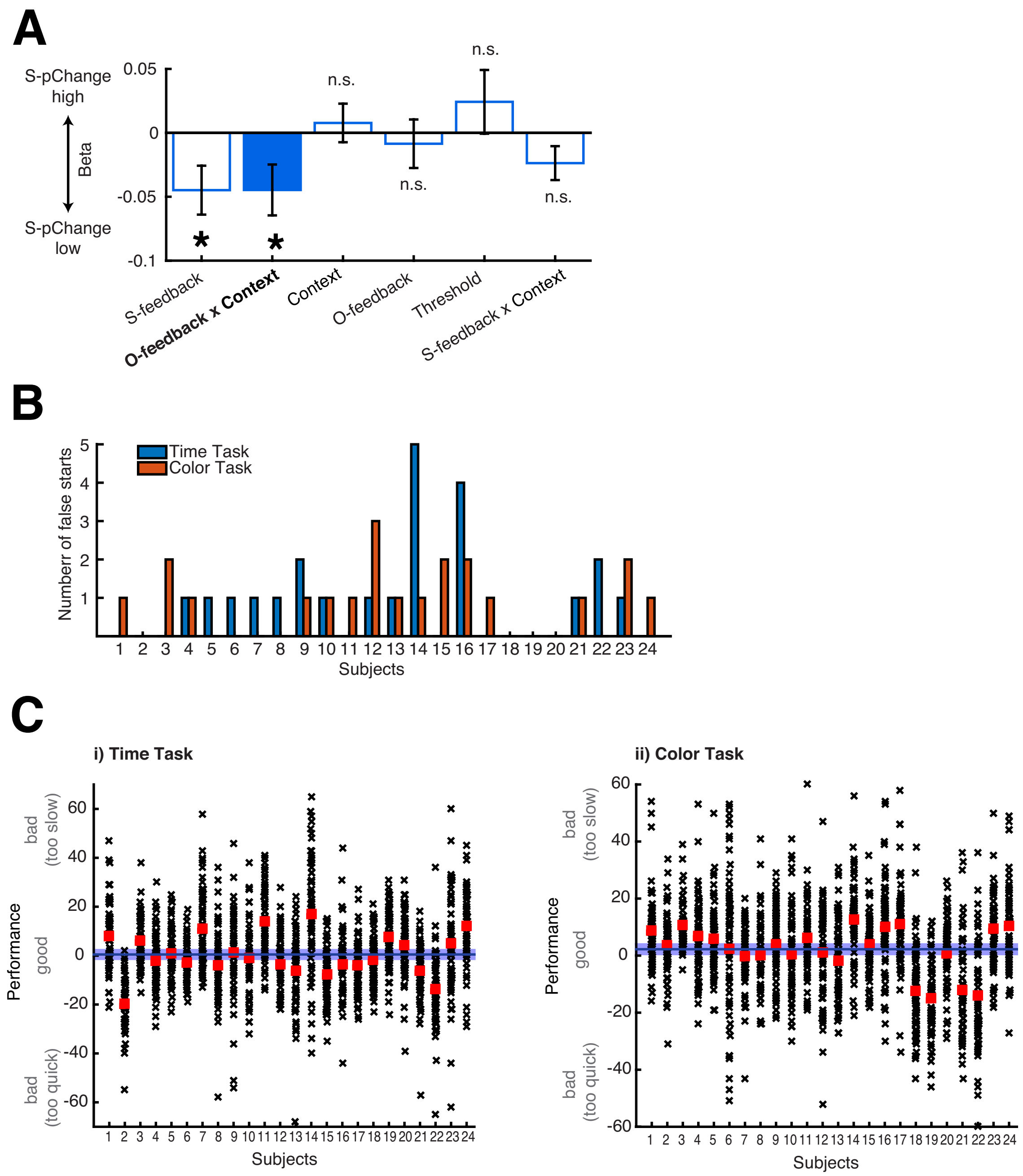

Figure S6 (relating to Figure 4)

Supplemental analysis of S-pChange and minigame performance. (A) Linear regression on S-pChange (S-pChange GLM1b, Supplemental Experimental Procedures 2). S-pChange was calculated within each minigame. This means that S-pChange reflects the absolute change in performance from a trial to the next trial of the same minigame; not across minigames. The filled blue bar indicates the SOM related effect. S-pChange from the current to the next trial was higher when subjects received performance feedback suggesting that they performed badly (S-feedback effect, t_{23}=-2.344 ; p=0.0281). The performance feedback for the relevant other (O) also had an influence on S-pChange, depending on the social context in which it was observed. Subjects changed their performance more when the O received negative performance feedback in cooperation (just as they did for themselves) and positive performance feedback in competition (O-feedback x Context, t_{23}=-2.246 ; p=0.0346). The beta weights of this latter regressor were tested in Figure 4B (*O-influence on S-pChange*) and correlated with behavior in Figure 4D. Note that, for visualization purposes, the effect is sign-reversed in Figure 4D. **(B)** Number of false start trials per participant per minigame. **(C)** Signed minigame performances plotted for all subjects for all trials (black crosses), mean signed performance per subject (red dots) and mean signed performance for the whole sample (horizontal dark blue line; shaded dark blue indicates

standard error of the mean). False start performances are not shown. Note that the figure shows signed performance values, because although the absolute value is enough to indicate performance levels, the signed performances are necessary to calculate the change in performance from one trial of a minigame to the next trial of the same minigame (S-pChange, Supplemental Experimental Procedures 2). For both minigames, negative performance values indicate responses that were too quick and positive values indicate responses that were too slow. Zero indicates the optimal response time. Performances in time and color task were comparable with no differences in mean performance (t_{23}=-0.899; p=0.3778). This and the fact that the mapping of performance feedback schedules to minigames was balanced across subjects (11 received better performance feedback in the time task and 13 subjects received better performance feedback in the color task, see Figure S1A) makes it unlikely that S-pChange effects were driven by performance differences in the minigames. (error bars are mean +/-SEM; *, $p < 0.05$)

RL model fitted on ratings and engage/avoid decisions

Parameter	α	β
Median (SEM)	0.3906 (0.0359)	0.4684 (0.0489)

Table S1 (relating to Figure 2)

Supplemental reinforcement learning model results. Summary of parameter estimates (SEM is standard error of the mean).

Supplemental Experimental Procedures.

1. Reinforcement learning model

For every subject, we fitted a standard reinforcement learning model to model the performance estimates assigned to the three players trial by trial (Self, S; Other1, O1; Other2, O2). We used two minigame specific (Color Task, T1; Time Task, T2) performance estimates per player. The performance estimates summarize the previous performance history and are hence referred to as *performance*. They reflect the expected performance based on a recency-weighted average of past performance feedback. This resulted in six player and minigame specific *performance* estimates: $performance_{S\text{-}T1}$, $performance_{S\text{-}T2}$, $performance_{O1\text{-}T1}$, $performance_{O1\text{-}T2}$, $performance_{O2\text{-}T1}$, $performance_{O2\text{-}T2}$. On every trial t, the three *performance* estimates associated with the current minigame were updated using a prediction error (PE) based learning rule with a learning rate α as a free parameter:

1) $$Performance_{t+1} = performance_t + \alpha \times PE_t$$

(formula was applied separately for S, O1, O2, given T1 or T2)

The PE itself was calculated based on the specific *performance* estimate and performance feedback of the player in the current minigame as:

2) $$PE_t = feedback_t - performance_t$$

(formula was applied separately for S, O1, O2, given T1 or T2)

In false start trials, the *performance* estimate for S was not updated and remained unchanged until the next trial of the same minigame. No PEs for S were calculated for false start trials (the other players never displayed false start trials). For the first trial of the session for each player for each minigame, no *performance* estimates and prediction errors were calculated, but the initial performance feedbacks were used as the starting *performance* estimates for the respective player in the respective minigame.

In each trial after the four starter trials, subjects made a decision about cooperating or competing (depending on the current context) with the relevant other (O) and in addition provided ratings of both S and O. Both engage/avoid decisions and ratings were modeled based on *performance* estimates for S and the O, called *S-performance* and *O-performance*. *S-performance* is the *performance* estimate for Self associated with the minigame of the current trial ($performance_{S\text{-}T1}$ or $performance_{S\text{-}T2}$). Similarly, *O-performance* refers to $performance_{O\text{-}T1}$, $performance_{O1\text{-}T2}$, $performance_{O2\text{-}T1}$ and $performance_{O2\text{-}T2}$, depending on which other player was currently selected as the O and which minigame took place. Therefore, *S-performance* and *O-performance* represented minigame and player specific performance expectations of the players involved in the current trial's engage/avoid decision. The same was the case for the PEs associated with S and O.

Subjects' ratings of a player reflected expectations of whether they would perform either better or worse than a level indicated by a *rating marker* the position of which was adjusted from trial to trial using a staircase procedure explained in Experimental Procedures ("Ability ratings"). Expectations expressed in the ratings that exceeded or fell below the rating marker were referred to as positive and negative ratings, respectively. To calculate the probability of a positive rating (p(positiveRating)), we used a softmax function with an inverse temperature β. This was done separately for S and O using *S-performance* and *O-performance*, respectively as well as the player specific rating marker:

3) $$P(positiveRating) = \frac{\exp[\beta \times (performance - ratingmarker)]}{\exp[\beta \times (performance - ratingmarker)] + 1}$$

(this formula was applied separately for *S-performance* and *O-performance* given their respective rating markers)

Having calculated the probability of a positive rating on a given trial, the probability of the rating actually observed was derived, again, separately for S and O:

4) $$P(rating) = \begin{cases} p(positiveRating) & \text{if rated positively} \\ 1 - p(positiveRating) & \text{if rated negatively} \end{cases}$$

(formula was applied separately for S and O)

Subjects also received a small gain or loss at the end of a trial if they had made a positive rating and the expectation indicated by that rating had been accurate (ratingbonus of 0.25 points). As explained in the Experimental Procedures, to ensure that there was no temptation to perform poorly in the task no ratingbonus was awarded when a negative rating had been given. The expected value of a rating (EV_{rating}) was calculated as

5) $$EV_{rating} = \begin{cases} [p(positiveRating) - 0.5] \times 2 \times ratingbonus & \text{if rated positively} \\ 0 & \text{if rated negatively} \end{cases}$$

(formula was applied separately for S and O)

Note the bounds of EV_{rating} for positive ratings are 0.25 and -0.25, which are the points that can be lost or won for positive ratings.

In addition to completing a rating for S and O on each trial, subjects made a decision to engage in or avoid cooperating/competing. Given the objective social context specific payoff scheme of the task (equations 1a and 1b in Experimental Procedures section), the subjective expected value of engaging in cooperation/competition (EV_{engage}) was calculated in an analogous way:

6a) $$Competition: \quad EV_{engage} = S\text{-}performance - O\text{-}performance - threshold$$

6b) $$Cooperation: \quad EV_{engage} = (S\text{-}performance + O\text{-}performance) / 2 - threshold$$

A decision to avoid cooperating/competing led to a gain of 1.5 points and a loss of 1.5 points with equal probability (see " Experimental design and schedule" in Experimental Procedures) and subjects had been instructed that the expected value of the decisions to avoid cooperating/competing was zero:

7) $$EV_{EAD} = \begin{cases} EV_{engage} & \text{if engage} \\ 0 & \text{if avoid} \end{cases}$$

Therefore, EV_{engage} was used as decision variable for the engage/avoid decisions to calculate the probability of engaging in cooperation or competition:

8) $$P(engage) = \frac{\exp(\beta \times EV_{engage})}{\exp(\beta \times EV_{engage}) + \exp(\beta \times EV_{avoid})}$$

Note that EV_{avoid} is zero in equation 8, as explained above. The probabilities of the actual choices made were derived from p(engage):

9) $$P(choice) = \begin{cases} P(engage) & \text{if engage} \\ 1 - P(engage) & \text{if avoid} \end{cases}$$

The full reward expectation on each trial (EV_{chosen}) was defined as the sum of the expected values from both ratings and the expected value of the engage/avoid decision (equations 5 and 7):

10) $$EV_{chosen} = EV_{S-Rating} + EV_{O-Rating} + EV_{EAD}$$

The reward prediction error (RPE) was calculated based on all reward outcomes of a trial including both rating reward outcomes and the engage/avoid decision reward outcome (see above equation 2 for the calculation of player specific prediction errors):

11) $RPE = \text{Reward} - EV_{chosen}$

Overall, the free parameter set θ comprised two free parameters: the learning rate α and the inverse temperature β. We fitted these parameters for every subject separately by minimizing the negative log likelihood (nLL) over all trials N, given a set of parameter values. For the calculation of nLL, we treated ratings and engage/avoid decisions equally. So the decisions used to fit the model included equal proportions of ratings of S, ratings of the O and engage/avoid choices to improve the model fit.

12) $nLL = -\sum_{n=1}^{N} \log(p(decision_t | \theta))$

2. Behavioral analyses

We used general linear models (GLM) to examine the impact of different factors on behavior. GLMs were applied to the ability ratings for (S and O ("Rating GLM for S/O"), to the engage/avoid choices ("Choice GLM") and to an index of true performance change over trials ("pChange GLM"). For all GLMs, all regressors were normalized (mean of 0 and standard deviation of 1).

Our first analyses aimed to show that subjects relied on performance feedback for their ratings and engage/avoid choices. For this, we predicted S and O ratings on the basis of S's and O's recent performance feedback history (last four trials; referred to as "feedback" below) at trial t. In addition, the GLMs contained the value indicated by the rating marker (the rating marker indicated a performance level with reference to which the rating should be made; see "Ability ratings" in Experimental Procedures):

Rating GLM 1 for S:
feedback-S_{t-1}, feedback-S_{t-2}, feedback-S_{t-3}, feedback-S_{t-4}, ratingmarker-S (Figure 2B-i)

Rating GLM 1 for O:
feedback-O_{t-1}, feedback-O_{t-2}, feedback-O_{t-3}, feedback-O_{t-4}, ratingmarker-O (Figure 2C-i)

As an aggregate index of performance feedback, we fitted a reinforcement learning (RL) model on the rating data (see Supplemental Experimental Procedures 1 for details on the RL model) and used *S-performance* (recency-weighted performance estimate for self) and *O-performance* (recency-weighted performance estimate for relevant other) to explain engage/avoid decisions. For this analysis, the RL model was fitted on the ratings only and not on the engage/avoid decisions (all other analyses including neural analyses were fitted on both ratings and choices to improve model fit). We applied the same GLM separately to cooperative and competitive trials:

Choice GLM1 - binned by social context (cooperate/compete):
S-performance, O-performance, threshold (Figure 2D)

The second set of analyses aimed to investigate more subtle effects of self-other-mergence (SOM) in the rating data. Again, we used *S-performance* and *O-performance* from the RL model. Note that the SOM GLM effects are orthogonal to the fitting done in the RL model and all SOM related effects we present in this manuscript can be shown without the use of a reinforcement learning model i.e. by using past performance feedback directly to explain choices (Figure 4B and S6A, Figure S4B). The following analysis was restricted to engage trials only (a supplementary analysis with the same results using all trials including those on which subjects refrained from cooperation and competition can be found in Figure S4C). We focused first on engage trials because the social context is critical on these trials (rather than on "avoid" trials when subjects simply took the default option of a random payment). The GLM was applied separately to cooperate and compete trials:

Rating GLM2a for S - binned by social context (cooperate/compete):
S-performance, O-performance, ratingmarker-S (Figure 2B-ii)

Rating GLM2a for O - binned by social context (cooperate/compete):
O-performance, S-performance, ratingmarker-O (Figure 2C-ii)

Note we used *S-performance* in the S rating and *O-performance* in the O rating as control parameters and the resulting beta weights for those regressors were only used as indices of individual variability, for instance to establish correlations with brain signals; we did not test such effects for significance as they had been fitted on the same ratings. The main aim of this GLM was to assess whether ratings indicated, in addition to agent-consistent effects, an inappropriate influence of the performance history of the different player (underlined above; for example an influence of *O-performance* on the S rating, or an influence of *S-performance* on the O rating). The binned rating GLM2a was used for visualization. However, as we were interested in the differences of agent misattributed effects in cooperate and compete conditions (i.e. the interaction by social context), we devised an analogous rating GLM2b that was not binned by social context, but instead contained interaction terms of regressors of interest with social context. These interaction effects were tested for significance of SOM_{int} ("int" denotes "interaction"). To calculate the interaction terms, *S-performance x Context* for

example, *S-performance* was normalized and multiplied with 1 for cooperate trials and -1 for compete trials (the number of cooperate and compete trials was identical over the whole session); the same was done for *O-performance*. Hence, a positive interaction effect indicated that the effect of S-performance was stronger in cooperation than in competition. For analyses restricted to engage trials the 1/-1 term indicating cooperation or competition was normalized as well to account for possible differences in trial number. GLM2b, like GLM2a, was restricted to engage trials.

Rating GLM2b for S - with interaction by social context:
S-performance, O-performance, S-performance x Context, O-performance x Context, Context, ratingmarker-S
(SOM_{int}(O-->S) is underlined; Figure S4A)

Rating GLM2b for O - with interaction by social context:
S-performance, O-performance, S-performance x Context, O-performance x Context, Context, ratingmarker-O
(SOM_{int}(S-->O) is underlined; Figure S4A)

Additional versions of rating GLM2b that did not use an RL model (Figure S4B) or are based on all trials (instead of engage trials only; Figure S4C) are presented in Figure S4.

We also analyzed performance in the minigames (see Figure S3 for details on the minigames and their performance definitions). For these analyses, we used a measure of subjects' true performance change (S-pChange) from any trial, t, to the following trial of the same minigame, t+1. S-pChange for a given trial t was calculated as the logarithm of the absolute performance difference:

$$S\text{-}pChange_t = \log(1 + |performance_{t+1} - performance_t|)$$

The performance measure was signed with zero indicating optimal performance while positive and negative values indicated responses that were too slow or too quick, respectively (Figure S6). The use of predetermined performance feedback schedules meant that performance feedback could not be improved by more optimal performance in the minigames (except false start trials as a case of veridical performance feedback, see Figure S2C and Figure S6B showing the number of false start trials per subject). This made it possible to use S-pChange as a measure of behavioral adjustments based on performance feedback. Performances from false start trials were treated as outliers and therefore no S-pChange for a false start trial and the trial directly preceding it were calculated. Note that the performance measures for both minigames were comparable and the performance feedback schedules were balanced over both minigames (Figure S6). The main aim of this set of analyses was to investigate whether behavioral adjustments were also influenced by performance feedback for the O depending on the social context (cooperation or competition). For this, similar to rating GLM2, we used two analogous versions of a GLM; one applied to cooperate and compete trials separately for visualization, and one version applied to all trials to test interaction by social context effects for significance. We investigated the relationship of S-pChange (for trial t indicating the transition from trial t to t t+1, see definition of S-pChange above) to the three variables that determine reward outcomes in engage/avoid decisions: S performance feedback, O performance feedback and threshold (all for trial t)

S-pChange GLM1a - binned by social context on trial t (cooperate/compete):
S-feedback, O-feedback, threshold (Figure 4B)

As in previous analyses, significance of effects was calculated over all trials, using the interaction effect of performance feedback and social context on trial t.

pChange GLM1b - all trials:
S-feedback, O-feedback, S-feedback x Context, O-feedback x Context, Context, threshold
(SOM-related effect is underlined; Figure S6A)

Note that when using the threshold regressor over all trials, we combined the threshold regressors from cooperate and compete conditions and normalized them separately for each condition, as in the following neural analyses. The resulting regressor was also again normalized to have a mean of zero

and standard deviation of 1. S-pChange was normalized separately for individual subjects in these GLMs. Positive interactions by context again indicated stronger influence of the performance feedback on cooperate compared to compete trials.

3. MRI data acquisition and analysis

3.1 MRI data acquisition and preprocessing. Imaging data were collected on a 3 Tesla Siemens MRI scanner using a 32 channel head coil. T1-weighted structural images were acquired with the settings TR=3sec, TE=4.75mssec, TI = 1100msec, 1x1x1mm voxel size, 256x176x224 grid. Functional images were acquired using a Deichmann echo-planar imaging (EPI) sequence with TR=3s, TE=30 ms, 3x3x3mm voxel size, 87° flip angle, 30° slice angle and z-shimming to reduce signal distortions as well as dropout in medial orbitofrontal areas (Deichmann et al., 2003).

We used FMRIB's Software Library (FSL) to analyze imaging data (Smith et al., 2004). FMRI data preprocessing comprised spatial (Gaussian using full-width half maximum of 5 mm) and temporal filtering (3 dB cut-off at 100sec), motion correction with FSL's MCFLIRT and filtering of noise components after visual inspection using FSL's MELODIC. In a two-step procedure via subjects' individual structural MRI images, preprocessed functional data were nonlinearly registered to Montreal Neurological Institute (MNI) space.

3.2 MRI whole-brain analyses. We used FSL FEAT (Smith et al., 2004) for first level analyses. fMRI data were pre-whitened with FSL FILM to account for temporal autocorrelations. Motion regressors from MCFLIRT were included as nuisance regressors of no interest. Temporal derivatives of relevant regressors were included and the model was temporally filtered before it was applied to the data. Group results on the second level were calculated using FSL FLAME 1 with outlier de-weighting and a cluster-forming threshold of z>2.5 and p<0.05.

We used a single fMRI GLM for whole brain analysis. The same design was used for subsequent region of interest (ROI) analyses. The GLM includes RL-based regressors, which were fitted individually on all ratings and engage/avoid decisions (see Supplemental Experimental Procedures 1 for details on the RL model). All parametric regressors were normalized (mean of zero, standard deviation of one). The two main phases of interest were the decision phase and the feedback phase.

The decision phase was defined as the time period from engage/avoid decision onset to the engage/avoid choice subjects made (phase 1,2 and 3 in Figure S2A-ii). We modeled the decision phase as a constant regressor and accompanying parametric regressors. The parametric regressors of interest comprised:

- *S-performance*
- *O-performance*
- *S-performance x Context,*
- *O-performance x Context,*
- Context (binary regressor; cooperation 1, competition -1)
- Threshold
- logRT

The two interaction terms and threshold were calculated as explained in the Supplemental Experimental Procedures section 2 "Behavioral analyses". logRT is the logarithm of the engage/avoid decision reaction time (see phase 3 in Figure S2A-i). The timing parameters for the parametric regressors were identical with the constant decision phase regressor, except for threshold and logRT. These regressors' onsets were delayed by one second, as the threshold was only revealed one second after engage/avoid decision onset and knowledge of the threshold was necessary to make an engage/avoid decision (see phase 2 in Figure S2A-i).

Decision phases from starter trials were excluded from the decision regressor and accounted for by a regressor of no interest. Correlations between parametric regressors are shown in Figure S5A (however, note that this correlation matrix also contains an additional regressor that was used in the ROI version of this design, $EV_{S\text{-}Rating+O\text{-}Rating}$, but not logRT, as the latter was not a regressor of interest).

We used two constant regressors with a duration of zero time-locked to the response of S and O rating to account for the rating events (Figure S5B-ii shows this effect for O). In addition we used parametric regressors accompanying these constant regressors accounting for the reward expectations associated with the ratings (EV_{rating} for S and O from equation 5 in Supplemental Experimental Procedures 1 for S rating and O rating, respectively).

The feedback phase was similarly modeled as a constant regressor and parametric modulators. Note that trial feedback was chunked in three components and presented in randomized order ("Feedback" in Experimental Procedures and Figure S2A-ii):

I) S and O performance feedback and rating reward outcomes
II) Engage/avoid decision reward outcome
III) Irrelevant other performance feedback

Duration of the constant feedback regressor was 2.5 seconds, the time window in which the three feedback components initially appeared (phase 2 onset to phase 4 onset in Figure S2A-ii). Parametric regressors were modeled as stick functions (i.e. duration of zero) time-locked to the appearance of the relevant feedback component. They comprised:

- *S-performance* (I)
- *O-performance* (I)
- S-PE (Prediction error for Self) (I)
- O-PE (Prediction error for relevant other) (I)
- O-PE x Context (I)
- Prediction error - irrelevant other (III)
- Context (I)
- S-pChange (I)
- EV_{chosen} (see equation 10; II)
- Reward prediction error (RPE; see equation 11; II)

Roman numerals in brackets after the regressors indicate to which feedback component a regressor was time-locked. Feedback phases from starter trials, from false start trials and from trials on which no S-pChange could be calculated (see "Behavioral analyses" in Supplemental Experimental Procedures 2) were excluded from the feedback regressor and modeled as events of no interest. Correlations between parametric regressors are shown in Figure S5.

In addition, the GLM contained three regressors of no interest. First, a regressor time-locked to all button presses, modeled as stick functions, to account for movement-related effects. Second, two regressors captured brain signals associated with each minigame, spanning the time period from minigame onset to response button press.

3.3 ROI analyses. ROIs had a radius of three voxels and were centered on peak voxels of significant clusters from the whole brain GLM (Table 1). For ROI analyses, we transformed MNI to subject space and extracted the pre-processed BOLD time courses, averaged per volume. The time courses were normalized, oversampled by a factor of 20 and time-locked to decision phase onset and feedback phase onset (same onset timings as the constant regressors in the whole-brain GLM). We applied a GLM to each time point and computed one beta weight per regressor and time point, resulting in a time course of beta weights for each regressor. We extracted individual variation in signal size at the time of the group peak signal in an analysis window of 4 to 13 seconds from decision and feedback phase onset to relate brain activity to behavior. All regressors were normalized (mean of zero, standard deviation of one) for all ROI GLMs.

The ROI GLM for the decision phase contained the parametric regressors as the whole brain GLM, and, in addition contained a parametric regressor that was the sum of the EV_{rating} (equation 5 in Supplemental Experimental Procedures 1, RL section) for S and O to account for the reward expectation associated with those events:

- *S-performance*
- *O-performance*
- *S-performance x Context,*
- *O-performance x Context,*
- Context (binary regressor; cooperation 1, competition -1)
- Threshold
- logRT
- $EV_{rating\text{-}S+rating\text{-}O}$

Note that the ROI GLM for the decision phase was restricted to trials in which subjects made and engage choice rather than to avoid cooperating/competing. We did this to relate peak signal sizes from effects of interest in this analysis to behavioral beta weights from rating GLM2b (Figure S4A), which was also calculated over engage trials only.

The feedback related ROI GLM contained the same feedback related parametric regressors as listed for the whole brain GLM:

- *S-performance*
- *O-performance*
- S-PE (Prediction error for Self)
- O-PE (Prediction error for relevant other)
- O-PE x Context
- Prediction error - irrelevant other
- Context
- S-pChange
- EV_{chosen} (see equation 10)
- Reward prediction error (RPE; see equation 11)

The GLM for the feedback phase was calculated over all trials. We investigated signal sizes at the time of the group peak of relevant regressors in relation to behavioral beta weights from S-pChange GLM1b (O-feedback x Context), which was also calculated based on all trials.

We used a leave-one-out procedure on the group peak signal of the beta time course to do significance testing and avoid temporal selection biases. For every subject, we took the average beta time course of the relevant regressor based on the remaining 23 subjects. We identified the (positive or negative) group peak in the analysis window of 4 to 13 seconds from phase onset and then took the beta weight of the remaining subject at the time of that peak. We repeated this for all subjects. Therefore, the resulting 24 "peak" beta weights were selected independently of the time course of the subject analyzed. We assessed significance using t-tests on these resulting beta weights. For correlations with behavioral beta weights, the individual neural beta weights at the time of the group peak were used.

To illustrate some of the correlations between neural beta weights and behavioral beta weights (Figure 3B-ii,iii, Figure 4D), we used a median split procedure, in which our group of subjects was subdivided in two groups of 12 subjects based on their neural beta weight being low (i.e. below the median) or high (i.e. above the median) at the time of the group peak. Mean and standard error of the behavioral beta weight of interest are shown in the bar plots of these figures (right hand side of Figure 3B-ii,iii, and right hand side of Figure 4D).

Supplemental References

Brewer, M.B. (1979). In-group bias in the minimal intergroup situation: A cognitive-motivational analysis. Psychol Bull 86, 307-324.

Deichmann, R., Gottfried, J.A., Hutton, C., and Turner, R. (2003). Optimized EPI for fMRI studies of the orbitofrontal cortex. NeuroImage 19, 430-441.

Denny, B.T., Kober, H., Wager, T.D., and Ochsner, K.N. (2012). A meta-analysis of functional neuroimaging studies of self- and other judgments reveals a spatial gradient for mentalizing in medial prefrontal cortex. J Cogn Neurosci 24, 1742-1752.

Faber, N.S., Häusser, J.A., and Kerr, N.L. (2015). Sleep Deprivation Impairs and Caffeine Enhances My Performance, but Not Always Our Performance: How Acting in a Group Can Change the Effects of Impairments and Enhancements. Pers Soc Psychol Rev.

Michael, E., de Gardelle, V., and Summerfield, C. (2014). Priming by the variability of visual information. Proc Natl Acad Sci U S A 111, 7873-7878.

Nieuwenhuis, S., Forstmann, B.U., and Wagenmakers, E.J. (2011). Erroneous analyses of interactions in neuroscience: a problem of significance. Nat Neurosci 14, 1105-1107.

Saxe, R. (2006). Uniquely human social cognition. Curr Opin Neurobiol 16, 235-239.

Smith, S.M., Jenkinson, M., Woolrich, M.W., Beckmann, C.F., Behrens, T.E.J., Johansen-Berg, H., Bannister, P.R., De Luca, M., Drobnjak, I., Flitney, D.E.*, et al.* (2004). Advances in functional and structural MR image analysis and implementation as FSL. NeuroImage 23 Suppl 1, S208-219.

Stasser, G., and Titus, W. (1985). Pooling of Unshared Information in Group Decision-Making - Biased Information Sampling during Discussion. J Pers Soc Psychol 48, 1467-1478.
