# Supplementary material for: Self-Other Mergence in the Frontal Cortex during Cooperation and Competition
Source: Neuron. 2016 Jul 20;91(2):482–93. doi: 10.1016/j.neuron.2016.06.022 (PMC4961240; doi:10.1016/j.neuron.2016.06.022)
Supplement: Document S2. Article plus Supplemental Information [file mmc2.pdf]

Article

Neuron

Self-Other Mergence in the Frontal Cortex during Cooperation and Competition

Highlights

- People confuse their own performance with the performance of others
- Cooperation leads to positive and competition to negative self-other confusion
- Confusion is reflected in area 9 indicating interdependent self-other processing
- Learning from own performance history is represented in perigenual ACC

Authors

Marco K. Wittmann, Nils Kolling, Nadira S. Faber, Jacqueline Scholl, Natalie Nelissen, Matthew F.S. Rushworth

Correspondence

marco.wittmann@psy.ox.ac.uk

In Brief

Wittmann et al. find that we confuse own performance with the performance of others, depending on whether we cooperate or compete with them. This confusion is reflected in area 9, indicating it interdependently processes information about ourselves and others.

Wittmann et al., 2016, Neuron *91*, 482–493
July 20, 2016 © 2016 The Authors. Published by Elsevier Inc.
http://dx.doi.org/10.1016/j.neuron.2016.06.022

CellPress

Neuron
Article

Self-Other Mergence in the Frontal Cortex during Cooperation and Competition

Marco K. Wittmann,[1,*] Nils Kolling,[1] Nadira S. Faber,[1] Jacqueline Scholl,[1,2] Natalie Nelissen,[1,2] and Matthew F.S. Rushworth[1,3]
[1]Department of Experimental Psychology, University of Oxford, South Parks Road, OX1 3UD Oxford, UK
[2]Department of Psychiatry, University of Oxford, Warneford Hospital, OX3 7JX Oxford, UK
[3]Centre for Functional MRI of the Brain, University of Oxford, John Radcliffe Hospital, OX3 9DU Oxford, UK
*Correspondence: marco.wittmann@psy.ox.ac.uk
http://dx.doi.org/10.1016/j.neuron.2016.06.022

SUMMARY

To survive, humans must estimate their own ability and the abilities of others. We found that, although people estimated their abilities on the basis of their own performance in a rational manner, their estimates of themselves were partly merged with the performance of others. Reciprocally, their ability estimates for others also reflected their own, as well as the others', performance. Self-other mergence operated in a context-dependent manner: interacting with high or low performers, respectively, enhanced and diminished own ability estimates in cooperative contexts, but the opposite occurred in competitive contexts. Self-other mergence not only influenced subjective evaluations, it also affected how people subsequently objectively adjusted their performance. Perigenual anterior cingulate cortex tracked one's own performance. Dorsomedial frontal area 9 tracked others' performances, but also integrated contextual and self-related information. Self-other mergence increased with the strength of self and other representations in area 9, suggesting it carries interdependent representations of self and other.

INTRODUCTION

Social environments require humans and other primates to monitor others (Chang et al., 2013a, 2013b; Ruff and Fehr, 2014) and to know not only their own abilities, but also the abilities of others. This knowledge guides establishment of social dominance hierarchies (Zink et al., 2008) and can be linked to features of brain structure and function (Noonan et al., 2014; Sallet et al., 2011). It guides animals' choices in a powerful way. For example, the decision to engage in fundamental modes of social interaction such as cooperation or competition with a conspecific is guided by knowledge about their abilities and social status relative to one's own (Wang et al., 2011). Estimating abilities of both self and others on the basis of past performance may be particularly important for humans, as they are able to coordinate and execute multi-step tasks such as building a shelter together with others (Misyak et al., 2014; Tomasello et al., 2005) or making complex decisions in groups (Kerr and Tindale, 2004). Moreover, they have to use this knowledge flexibly because social alliances can change very quickly: a competitor within one's own company might quickly become a cooperator when competing with a different company.

It has been a long-standing idea in psychology that humans derive expectations about whether they will succeed in a given task from their past task performance (Bandura, 1977). At the same time, people do not learn about themselves in isolation but relative to their social environment. Comparisons with other people can be used as an effective means for self-evaluation (Festinger, 1954; Mussweiler, 2003), and, conversely, people base judgments of other people on knowledge of their own traits (Allport, 1924; Krueger, 2010). However, the social influence on judgments of self and others can vary. We might be influenced more strongly by others simply because we like them (Heider, 1958); we might, by default, evaluate members of our own group more positively than members of a different group (Brewer, 1979), and we might perceive others as more similar to us when we cooperate with them than when we compete with them (Toma et al., 2010). In sum, learning about self and others is often based on all three aspects: objective experience, self/other comparisons, and the social context.

In the field of neuroscience, we are only beginning to explore the computational and neural mechanisms that underlie how people learn about the abilities of other people (Boorman et al., 2013), but even less is known about how we learn about our own abilities. Reward-related brain signals scale with the payoff for oneself relative to the payoff of other people (Fliessbach et al., 2007). Recently, it has been shown that one's own choice preferences can be biased toward the observed choice preferences of other people (Garvert et al., 2015; Nicolle et al., 2012) and that this depends on the identity of the other person consistent with psychological theories (Izuma and Adolphs, 2013). However, unlike choice preferences, where there is no clear right and wrong, there is often objective information available about our own abilities. For instance, the time one needs to run 100 m ought to be well predicted by the previous occasions on which one ran 100 m. In this sense, ability judgments can be based on objective performance feedback attributable unambiguously to ourselves.

Here, we test, first, whether we estimate our abilities from monitoring our performance over time just as we estimate the values of our actions from monitoring their outcomes (Daw

This is an open access article under the CC BY license (http://creativecommons.org/licenses/by/4.0/).

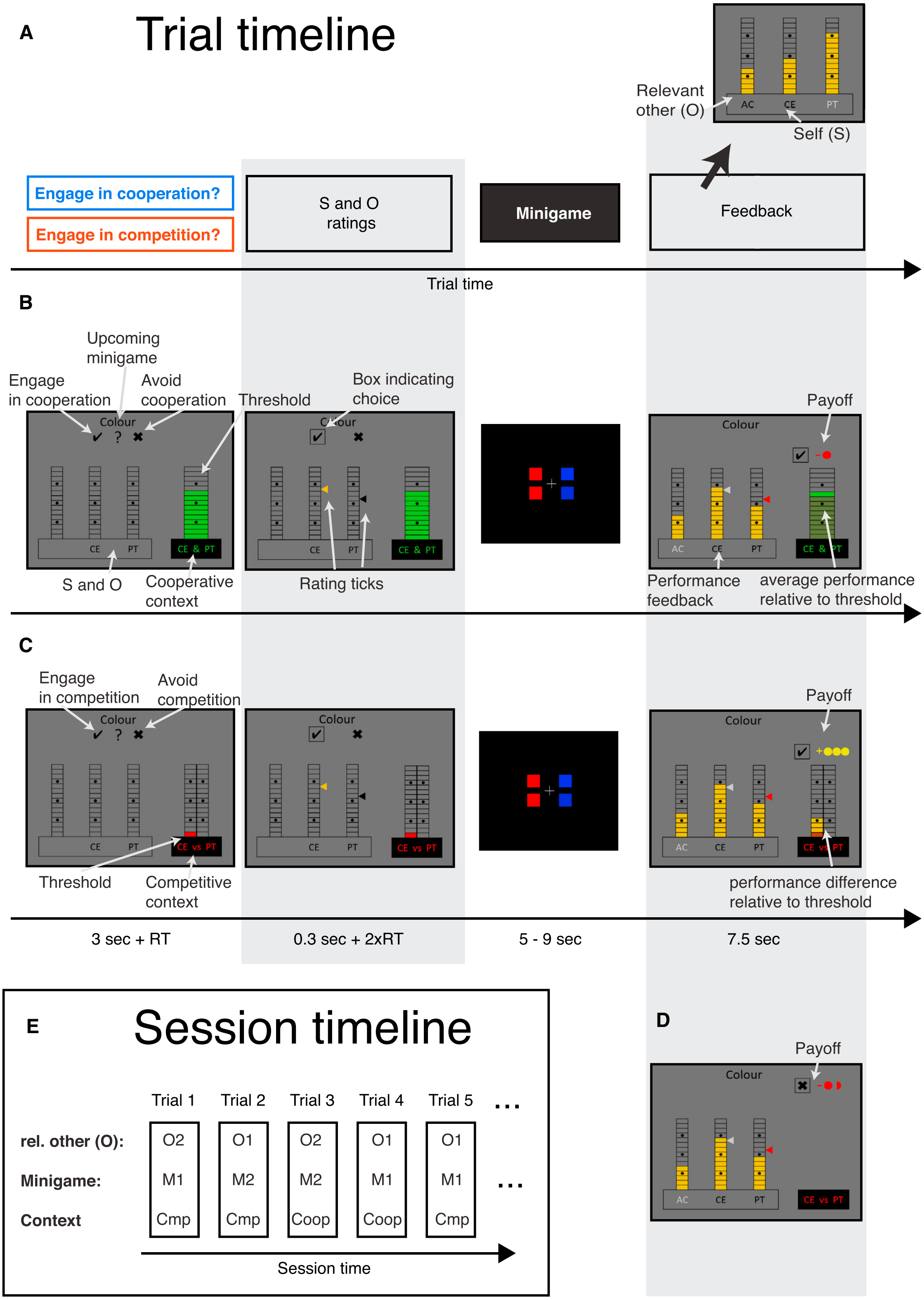

Figure 1. Task Design

(A) Schematic of trial events. On every trial, all players played a minigame, after which parametric performance feedback was provided (upper right; higher bars indicate better performance) that enabled performance to be learned. Letters indicate subject initials for Self (S; middle position) and two other players. A relevant

(legend continued on next page)

et al., 2011). We show that the history of an individual's performance is indeed used to estimate their ability. Second, we test whether ability estimated for oneself is also dependent on the performance of other people. We find that, surprisingly, the performances of other people also influence individuals' assessments of their own abilities. When we are cooperating with someone who performs well, our estimates of our own ability are inflated, and when we are cooperating with someone who performs poorly our ability estimates are depressed. Reciprocally, the ability of another individual is estimated not only from the other's performance, but also from one's own performance. We demonstrate that such self-other mergence not only impacts on people's subjective evaluation of themselves and others, but even affects how they subsequently adjust their performance. We refer to ability estimation for self and others based on their respective performance history as "appropriate," while we refer to the misattribution of past self-related performance when estimating others' abilities (or other-related performance to estimate one's own ability) as self-other mergence (SOM; see Figure 2A for an illustration).

Human subjects performed an experiment in an MRI scanner. Distinct regions in medial frontal cortex (Neubert et al., 2015) tracked the estimated abilities of self (perigenual anterior cingulate cortex; pgACC) and other (area 9) (Kelley et al., 2002; Mitchell et al., 2006). SOM increased with the strength of self and other representations in area 9; its activity predicted both how much self-judgments were related to the other player, and how much other judgments were related to one's own performance. This suggests that area 9 does not simply represent other people's perspectives independently of our own (Amodio and Frith, 2006), but instead it represents self and others in an interdependent fashion.

RESULTS

Experiment Structure

On each trial, subjects performed a reaction-time minigame. They were told that two other players independently performed the minigame at the same time (Figures S1–S3). We explain the nature of the minigames in detail in Figure S3. The minigames' precise nature is less critical than the fact that they provided a vehicle to investigate how subjects developed an estimate of their ability that was based on their performance and how this changed depending on interactions with two other players whose performances they also saw. On each trial in the experiment, subjects performed a short trial of a minigame and parametric feedback about their own performance and the performance of the other players was provided at the end of each trial (Figure 1A). Subjects could use the performance feedback to form ability estimates for self and the two others over the course of the experiment.

We used pre-determined performance feedback schedules to carefully match observed performance for self and others and to keep them stable across subjects. This ensured that performance learning for self and others were comparable and that individual differences in task behavior were interpretable. Subjects were told that the performance feedback reflected their objective performance mapped on a 15-point performance scale and that the previously established mapping was the same for all players. Therefore, subjects received explicit and independent performance feedback for all players. Using not only one but two minigames ("time task" and "color task") in pseudorandom trial order made it possible to have, on the one hand, slowly drifting performance shifts within a minigame (as abilities are thought to be relatively stable features [Boorman et al., 2013]) but, on the other hand, reduced sequential correlations across trials (by switching between minigames that were performed at different levels; Figure 1E) and ensured a full parametric range of performance feedback, thereby making it possible to perform event-related fMRI analysis. Finally, having two minigames allowed us to establish the generality of our findings.

Each trial was performed either in a cooperative or a competitive context. For example, on some trials subjects were given the opportunity to cooperate with one of two other players in the next run of the minigame (Figure 1B). This meant that their own performance score and the other player's performance score would be summed together, and if it exceeded a threshold indicated on the screen (varying trialwise), then points were awarded (which were translated into monetary reward at the end of the experiment). Subjects could first decide whether to engage in cooperation or whether to avoid it (engage/avoid decision). If they avoided cooperation, then the points awarded

other (O) was pre-specified on each trial but changed between trials. Ratings and minigames were embedded in engage/avoid decisions that established either a cooperative or competitive context.

(B) Trial timeline for an example cooperative trial. Trials start with the presentation of S, O, the upcoming minigame, social context, and the threshold. A decision is made about whether to cooperate or not (in this case the subject decided to cooperate). After the decision, subjects provided, in randomized order, *S-ability* and *O-ability* ratings. Then all players play a minigame (independent of choice or ratings). In the feedback phase, the payoff (−1 in this instance) is determined by the disparity between the performance average of S and O (9) and the threshold they were asked to exceed (10). Payoffs were parametric and could be positive, zero, or negative.

(C) Trial timeline for an example competitive trial. Annotations in the figure panel indicate elements differing from the cooperative example in (B). Again, an engage choice is made but now the decision is whether or not to compete. In this case, the subject chose to compete. Payoff (+3) is determined by the difference between the performance difference of S and O (4) and the threshold (1).

(D) If subjects decided not to cooperate or to compete (avoid choices), they nonetheless went on to make ratings and perform minigames. In the feedback phase, the context was still displayed (competitive in this example), but no threshold was shown, and the payoff was independent of performance scores. On such trials, subjects either won or lost 1.5 points with equal probability (i.e., the expected value was zero). Hence, choices should only depend on the expected value of cooperating or competing (depending on which context was indicated).

(E) Schematic of session timeline. Each trial is characterized by one of two other players having the role of relevant other (O1/O2) and one of two possible minigames (M1/M2) and has either a cooperative (Coop) or compete (Cmp) social context. All three trial features were experimenter determined and pseudorandomly interleaved.

were randomly distributed around zero. The other player was pre-specified on each trial and is referred to as "relevant other" (O) as opposed to the "self" (S). Specifying only one of the other players as O on any given trial, and changing this from trial to trial, also helped reduce sequential dependencies between performance estimates on successive trials. Whether or not a subject collaborates on any trial should depend on the subject's estimate of their own ability, their estimate of the other player's ability, and the threshold that has to be reached.

On other trials, subjects were given the opportunity to compete the relevant other, O, in the next run of the minigame (Figure 1C). This meant that the difference between their own performance score and the other player's performance score would be taken, and if it exceeded a threshold indicated on the screen, then points were awarded. Subjects could first decide whether to engage in competition or whether to avoid it (engage/avoid decision). If they avoided competition then the points awarded were randomly distributed around zero. Again, which of the two other players was O varied from trial to trial. And again, whether or not a subject competes should depend on the subject's estimate of their own ability, their estimate of the other player's ability, and the threshold score that has to be reached.

In summary, the engage/avoid decision created either a cooperative or a competitive relationship between S and O; O became either an ally or an opponent for S on that trial (Figures 1B and 1C). The context varied pseudorandomly across trials making sure that slowly drifting ability estimates were comparable between competitive and cooperative trials. Importantly, the inclusion of the engage/avoid decision and the threshold enabled us to distinguish S and O related brain activity from brain activity related to reward processing.

So that we could measure subjects' absolute ability estimates, subjects were asked on each trial to rate the performance they expected for the current trial for themselves (*S-ability*) and the relevant other (*O-ability*; Figures 1A–1C). *S-ability* and *O-ability* were assessed via two independent ratings (in random order) prior to performing the minigame. Having the ability ratings embedded in either a cooperative or a competitive context allowed us to test whether past performance of S and O had differential effects on the *O-ability* and *S-ability*, respectively. We used ratings in which subjects rated the players relative to a rating marker, which was updated over trials using a staircasing procedure (see "Ability ratings" in Experimental Procedures) to maximize both informativeness and speed of the ratings (and therefore trial number and statistical power). The inclusion of two different other players who took turns to be the relevant other in a trial ensured that S's and O's performance feedback and associated ability estimates were statistically decorrelated allowing identification of neural correlates in an unconfounded manner (Figure S5A).

Appropriate Ability Estimation and Self-Other Mergence

We analyzed rating and decision data using logistic general linear models (GLMs) applied first to each subject separately and then averaged resulting regression weights (beta weights) across subjects (Figure 2). After testing whether both ability judgments in the rating data and engage/avoid choices were based on the previous performances of the appropriate players, we went on to investigate self-other mergence (Figure 2A).

First, we tested which information subjects used to estimate self and others' abilities. Analysis of the rating data showed that *S-ability* and *O-ability* were based on the performance history of S and O, respectively. This indicates that subjects indeed used performance feedback to generate predictions about their subsequent minigame performance (Figures 2B-i and 2C-i). Recent performance feedback was predictive of subjects S and O ratings. However, performance that had occurred more remotely in time had much less of an impact on the subjects' ability estimates. This finding that recent and remote events have greater and lesser impacts, respectively, is similar to the finding that action value estimates are based more on recent reward in reinforcement-type learning (Sutton and Barto, 1998). Therefore, to summarize the performance histories that subjects had observed, we fitted a standard reinforcement learning (RL) model individually for each subject (Supplemental Experimental Procedures 1). *S-performance* and *O-performance* from the RL models were estimates of expected performance and represented recency-weighted averages of the performance feedback (not the true performance) of S and O.

Next, we examined whether *S-performance* and *O-performance* from the RL models predicted decisions to engage in cooperation/competition. We found that a better *S-performance* increased the likelihood of cooperating and competing, while a better *O-performance* led to increased cooperation and decreased competition (Figure 2D). The third factor, the threshold that subjects had to exceed on each trial, not surprisingly also influenced behavior; a higher threshold led to less engagement in both cooperative and competitive contexts. As incentivized by the experimental design, subjects' engage rates were around 70% (Figure 2D-ii), and we found a slight preference for cooperation compared to competition ($t_{23} = 3.82$; $p < 0.01$). This preference might be related to the experiment's payoff structure. However, it is also consistent with suggestions that a normative cooperative bias exists in social interactions promoting cooperation over selfish behavior (Boyd and Richerson, 2009). This shows that choices when to cooperate and when to compete, like the subjects' ability ratings, were strongly based on the previous performances of S and O. It also indicates that our competition/cooperate manipulation had the expected impact on subjects' behavior: subjects preferred to cooperate with high performers and to compete with low performers, as they should indeed have done in order to maximize their reward in the experiment.

Finally, we investigated whether subjects' own ability estimates were also related to O's past performance. We focused our analysis of SOM on trials where subjects chose to engage in cooperation or competition. Controlling for *S-performance*, we assessed the influence of *O-performance* on *S-ability*. To do this, we tested whether, in a GLM which contained factors indexing past performance of S, past performance of O were predictive of subjects *S-ability* ratings. Specifically, we tested whether *S-ability* judgments were indeed biased toward the performance levels of O in cooperation, but away from them in competition. We found that, indeed, during cooperation *S-ability* increased and decreased in tandem with

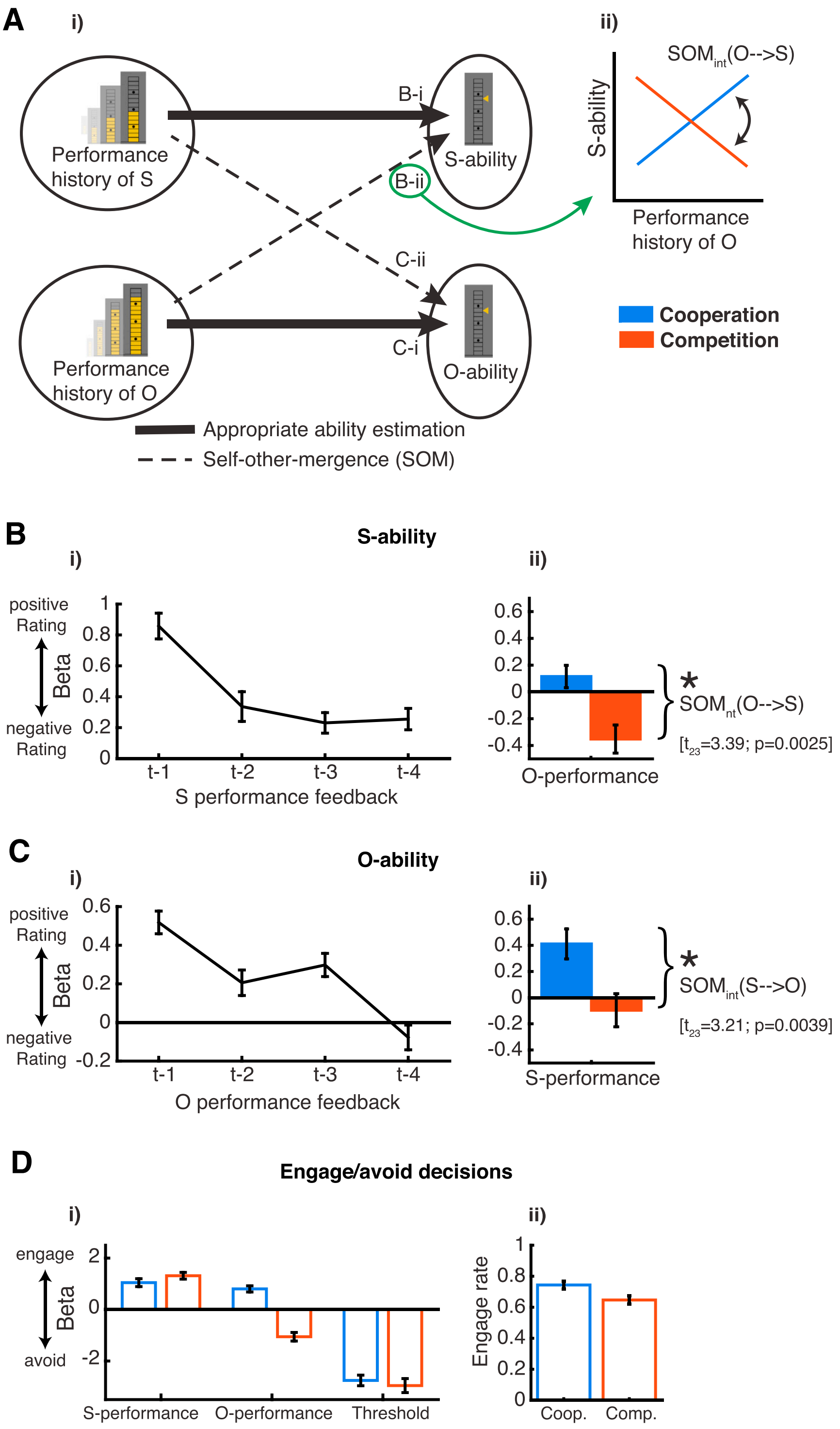

Figure 2. Behavioral Results

Blue and red indicate cooperation and competition trials in all panels.

(A) Schematic of appropriate ability estimation and self-other mergence (SOM) (i). Letters on arrows refer to subsequent panels showing analysis of appropriate ability estimates (B-i and C-i) and SOM effects (B-ii and C-ii). *S-ability* and *O-ability* refers to trialwise ratings of oneself (S) and relevant other (O). (ii) Illustration of a context-dependent SOM effect where O's past performance influences *S-ability* estimates. *S-ability* estimates are inflated or depressed when O's performance history has been, respectively, good or poor in cooperative contexts. However, the inverse is the case in competition: good performers decrease *S-ability* estimates. Positive and negative beta weights in B-ii (and analogously for *O-ability* in C-ii) reflect the increasing and decreasing slopes in this illustration.

(B and C) Agent appropriate estimation of ability in (B-i) and (C-i): *S-ability* and *O-ability* on trial t were based on recent S and O performance feedback, respectively. Panels show beta weights of a logistic GLM, applied to each subject and averaged over subjects (same for subsequent panels). Ratings were more strongly based on the recent performance feedback received by the appropriate player (i.e., *S-ability* ratings reflect S's past performance and *O-ability* ratings reflect O's past performance). SOM of performance in (B-ii) and (C-ii): we found an influence of *O-performance* on *S-ability*, controlling for the effect of *S-performance*. This effect reversed with social context such that the estimation of one's own ability was either inflated (in cooperation) or depressed (in competition) when paired with a high performer. Similarly, *S-performance* influenced estimates of *O-ability*, controlling for the influence of *O-performance* (significant SOM_{int}; p values for both analyses calculated in an interaction analysis from Figure S4A; same y axes as in (i). Note that different GLMs were used for (i) and (ii) (see main text and Experimental Procedures).

(D) Engage/avoid decisions. (i) *S-performance*, *O-performance*, and threshold influenced decisions to cooperate or compete in a rational manner. In particular, O is weighted in reverse fashion depending on whether O is an ally in a cooperative context or an opponent in a competitive context. (ii) Rate of engage choices for cooperative ("Coop.") and competitive ("Comp.") trials (error bars are mean ± SEM).

O-performance: independent of subjects' own performance, subjects evaluated themselves more positively when the other player performed well and more negatively when the other performed badly. This was reversed in competition: subjects evaluated themselves more negatively when competing with a high compared to a low performer, as demonstrated by an interaction effect ($SOM_{int}(O \rightarrow S)$, $t_{23} = 3.39$; $p = 0.0025$; see Figure S4A for full details and summary in Figure 2B-ii). We found *O-ability* was influenced by *S-performance* in a complementary manner ($SOM_{int}(S \rightarrow O)$, $t_{23} = 3.21$; $p = 0.0039$; see Figure S4A for full details and summary in Figure 2C-ii). The two SOM effects persisted both when using estimates of S's and O's performance history

Table 1. Peak Coordinates of Significant Clusters in Whole-Brain fMRI Contrasts

Contrast	Region	Peak Coordinates x/y/z (in mm MNI Space)	Z Value
S-performance	Perigenual anterior cingulate cortex (pgACC)	0 40 6	3.98
	Precuneus	−6 −64 18	3.38
O-performance	Brodmann area 9	2 44 36	3.43

Family-wise error cluster corrected, $z > 2.5$, $p < 0.05$.

that were not based on RL models and also in analyses that included the trials where subjects decided to avoid cooperation or competition (even though on such trials the relation to O might be less important, Figures S4B and S4C). Therefore, ability estimates for self and other were interdependent; SOM occurred both when judging oneself and the other individual.

Neural Correlates of Appropriate Ability Estimation and Self-Other Mergence

In close correspondence to the behavioral analysis, we tested, first, whether there were brain regions encoding ability estimates for self and relevant other. Having identified such regions, we went on to test whether self and other signals were related to the appropriate attribution of past performance to the relevant player (*S-performance* to S and *O-performance* to O) or whether they indicated a misattribution between self and other. For all MRI analyses, we used a single GLM (see Supplemental Experimental Procedures 3.3 for details).

In order to identify regions tracking performance history, we focused on the decision phase and used a GLM very similar to the one used in the behavioral choice analysis (Figure S5; Table 1). This allowed us to distinguish between brain signals related to the self, the relevant other, and the variable threshold subjects had to exceed to win points on each trial (Figure S5A). Activity in perigenual anterior cingulate cortex (pgACC) reflected subjects' own recent performance history (Figure 3A-i); it increased and decreased depending on how well subjects had themselves performed recently (and therefore how likely they are to perform well on the next trial). This *S-performance* signal in pgACC did not simply reflect individual choice values; it was present even after partialing out reward expected on a given trial (Figures S5B and S5C). Such an analysis was possible because the experimental design meant that on any trial reward expectation was only partly a function of *S-performance* (in addition it also depended on the threshold and *O-performance*, in different ways on cooperative and competitive trials).

In contrast, area 9 tracked *O-performance* history (Figure 3A-ii; Figures S5B and S5C), reflecting how well the relevant other is expected to perform in the minigames. Area 9 is part of the "theory of mind" network (Saxe, 2006) and is active during other-directed behavior (Sul et al., 2015). However, the effect of *O-performance* that we found in area 9 was a basic other performance tracking signal and as such is unlikely to reflect only emotional or motivational responses to the other player that vary as a function of context. As such, the *O-performance* signal could be distinguished from activity more generally related to O. For instance, area 9 and other nodes of the "theory of mind" network were also more active at the times when subjects rated the relevant other (Figure S5B-ii). Overall, the pattern of performance related signals was different in area 9 compared to pgACC (Figures S5C-ii and S5C-iii).

In all subsequent analyses, we focused on the same two regions of interest (ROIs) using the same GLM as used on the whole-brain level (although based on the same trials as in the behavioral analysis, Figures 2B-ii and 2C-ii) using time course analysis. To relate brain signals to behavior, we employed a two-step inference process considering only variables that were related specifically to past performance. First, we assessed whether an ROI carried a significant signal for a given regressor. Second, only if this was the case, we tested whether individual variation in the brain signal at the group peak was related to individual variation in behavior by correlating it with the respective behavioral beta weight from the self or other rating. We began by examining our main variables of interest: *S-performance* and *O-performance*.

We found that *S-performance* signal in pgACC predicted how much *S-performance* determined *S-ability* across subjects (Figure 3B-i; $r = 0.55$; $p = 0.0053$). Hence, pgACC signals reflected the use of information for estimating one's own abilities, independent of others. We found no other performance related representations of self and other in pgACC.

In contrast to pgACC, the *O-performance* signal in area 9 did not predict how much ability estimates of O were guided by *O-performance* ($r = 0.07$; $p = 0.74$). Instead, it predicted the degree to which subjects' *S-ability* decreased as a function of engaging with a strong other player (Figure 3B-ii; $r = -0.48$; $p = 0.0177$). This means that subjects with a stronger neural representation of the relevant other's past performance in area 9 rated their own ability more negatively the better the relevant other performs. At a behavioral level, this corresponded to the tendency for *O-performance* to have a negative main effect on *S-ability* (the average effect of the two bars in Figures 2B-ii and S4A). The generally negative effect of *O-performance* on *S-ability* becomes apparent when considering only subjects with the strongest *O-performance* signals in area 9 ($t_{11} = -2.5$; $p = 0.0293$; Figure 3B-ii, right; median split of subjects). Therefore, area 9 activity reflected SOM in the estimation of the abilities of oneself.

To investigate whether area 9 was generally related to SOM, we examined whether such an effect was also present in the other direction: that past *S-performance* was used to estimate *O-ability*. In addition to *O-performance* signals, area 9 also carried an *S-performance* signal that was stronger in competition than in cooperation (interaction effect; $t_{23} = 2.93$; $p = 0.008$; Figure 3A-iii). This was consistent with our finding that area 9 was generally more active when there was a competitive relationship between S and O than when there was a cooperative relationship (main effect of context; Figure S5B-iii). We went on to test whether these self-related performance representations were also linked to SOM. Indeed, we found that *S-performance* influenced *O-ability* via area 9: the neural *S-performance* effect in area 9 (Figure 3A-iii) was predictive of the influence exerted by *S-performance* on *O-ability* shown in Figure 2C-ii (Figure 3B-iii; $r = 0.43$; $p = 0.0341$). In other words, the neural signature of

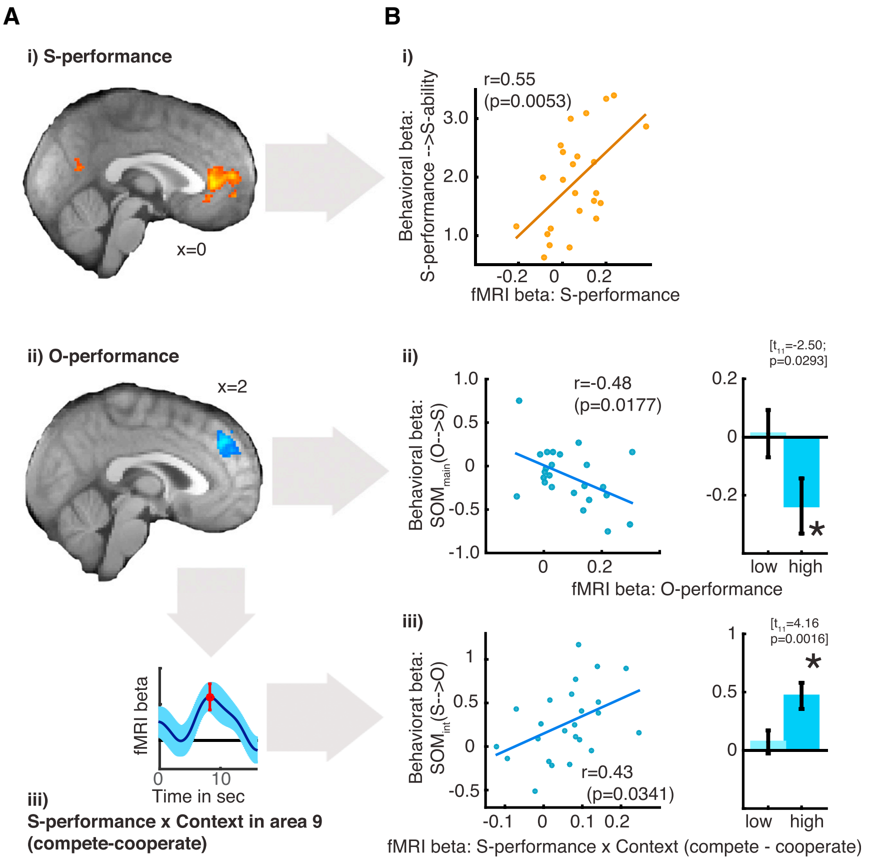

Figure 3. fMRI Results

Whole-brain effects family-wise error cluster corrected, $z > 2.5$, $p < 0.05$.

(A) Activation foci *for S-performance* in pgACC (i, yellow) and *O-performance* in area 9 (ii, blue). Area 9 showed more diverse social signals; a contextual *S-performance* signal was observed, which was stronger in competition than in cooperation (iii). The beta time course shows the context-dependent difference in *S-performance* (red line indicates signal group peak where beta weights were taken for correlations with behavior).

(B) Relationship between neural and behavioral effects. (i) In pgACC, across-subjects variability in *S-performance* signal reflected the influence of *S-performance* on *S-ability*. (ii, left) By contrast, variation in area 9 *O-performance* signal was not related to the degree subjects based *O-ability* on *O-performance*; instead, it predicted the average influence of *O-performance* on *S-ability* ($SOM_{main}(O \rightarrow S)$). Note the behavioral variable plotted on the ordinate corresponds to the average effect (across cooperation and competition) of *O-performance* on *S-ability* (see Figure 2B-ii). (ii, right) In other words, only subjects with high *O-performance* signal showed a negative effect of *O-performance* on *S-ability* (median split; same y axis as on the left). (iii, left) The strength of the contextual *S-performance* effect in area 9 (A-iii) predicted the degree to which *O-ability* was influenced by *S-performance* in a context-dependent manner ($SOM_{int}(S \rightarrow O)$ from Figure 2C-ii on y axis). (iii, right) Again, only subjects with higher *S-performance* interaction signals showed the corresponding behavioral effect of *S-performance* on *O-ability* (median split; same y axis as on the left) (error bars are mean ± SEM).

S-performance in area 9 was different depending on context, and this difference predicted the context-dependent effect of *S-performance* on *O-ability* ($SOM_{int}(S \rightarrow O)$).

In sum, we found that, at a first approximation, self and other signals were found in distinct brain regions (pgACC and area 9), but further investigation revealed area 9 contained more diverse social signals relating also to oneself but within the social context. We found two SOM effects in area 9 that indicate that it is those subjects with stronger self and other related activity that are prone to SOM (Figures 3B-ii and 3B-iii, right panels, statistics embedded in figure). The findings were specific to area 9 (Figure S5C). Therefore, the stronger the representation of the other in area 9, the more the judgment of one's own ability depended also on the other's performance; analogously, the strength of area 9 self-related representations predicted how much they influenced the estimation of other's abilities.

Behavioral Adjustments Driven by Self-Other Mergence in Area 9

Finally, we investigated whether self-other mergence had an impact beyond the subjective estimates of abilities reported by subjects and tested whether its influence even propagated to behavior, and, if so, whether such an effect could also be related to area 9.

We used the trial wise change in subjects' performance, regardless of direction (*S-pChange*; Figure 4A) as an index of feedback driven behavior strategy adjustment from one trial involving a given minigame to the next trial with the same minigame. Note that *S-pChange* refers to the change in true performance, whereas *S-performance* and *O-performance* summarize the performance feedback observed. Subjects changed their performance in the minigames (for better or worse) more after negative performance feedback for S ($t_{23} = 2.34$, $p = 0.0281$). However, they also changed their performance more after negative feedback for O in cooperative trials, while in competition they changed their performance more after more positive feedback for O (*O-influence* on *S-pChange*; $t_{23} = 2.25$, $p = 0.0346$; see Figure S6 for full details and summary in Figure 4B). This means that the performance feedback, displayed for the relevant other after a minigame, had an impact on how subjects performed the minigame themselves when they encountered it the next time. Therefore, subjects used the performance feedback pertaining to the other to adjust their own behavior.

We tested whether this behavioral SOM effect was again mediated by area 9 using an ROI analysis (coordinates taken from previous analysis). This final part of the analyses was specifically intended as a replication of previous SOM effects. We used the same GLM as before, focusing on the feedback phase

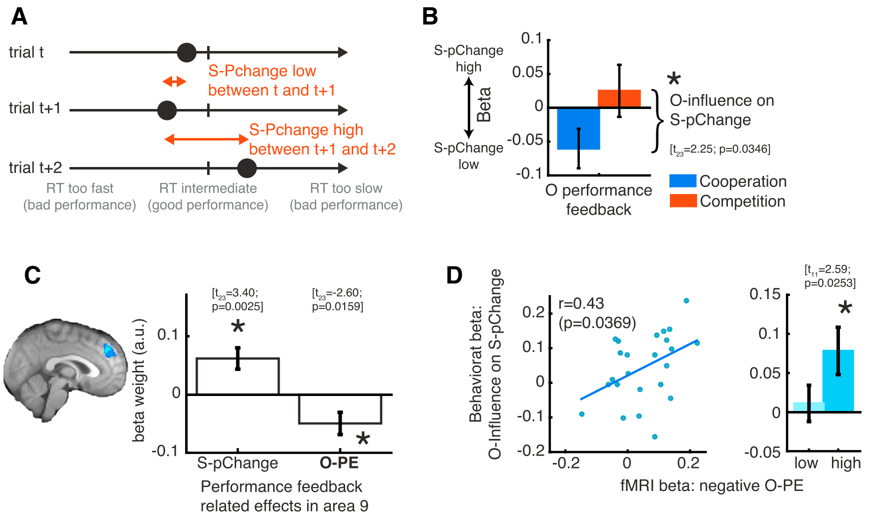

Figure 4. Self-Other Mergence in Minigame Performance Adjustments

(A) Illustration of *S-pChange* reflecting the absolute (i.e., unsigned) difference in true minigame performance from one trial to the next of the same minigame. *S-pChange* constitutes an index of how much subjects changed their true minigame performance as a function of performance feedback.

(B) Subjects' performance adjustments were guided by their own performance feedback. *S-pChange* increased with more negative performance feedback (see main text). However, O performance feedback exerted an additional influence on *S-pChange*. It influenced *S-pChange* in a manner that paralleled its influence on *S-ability* (Figure 2; p value from Figure S6A).

(C) BOLD activity in area 9 in the feedback phase of trials was related to the magnitude of *S-pChange* that would ensue from the current to the next trial (main effect). It also scaled negatively with a prediction error for O (O-PE). ROI taken from Figure 3A-ii.

(D) Subjects with stronger (more negative) O-PE in area 9 were more influenced by O performance feedback in their subsequent minigame performance (left). As in the earlier results, SOM occurred only in subjects with stronger area 9 signal (median split; same y axis as on the left) (error bars are mean ± SEM).

this time (Figure S5D). Again, we first aimed to identify activity related to the relevant other's performance in area 9 and only subsequently to relate such activity to how much subjects were biased by the other player. First, we tested whether area 9 could play a role in preparing behavioral adjustments from trial to trial. We found that, indeed, in the feedback phase of the trial, activity in area 9 was correlated with the extent that subjects changed their performance from the current trial to the next trial (main effect *S-pChange*; Figure 4C). Second, at the same time, we found a negative signal scaling with the size of the (signed) prediction error for the relevant other (O-PE; Figure 4C). O-PE indexed how much the observed performance feedback for O deviated from subjects' expectation of O's performance level (Suzuki et al., 2012). Third, variation in O-PE signal predicted variation in *O-influence* on *S-pChange* (Figure 4D; $r = 0.43$, $p = 0.0369$). This replicates our previous findings linking area 9 to self-other mergence: the strength of other-related performance signals in area 9 predicted how much subjects' adjusted their own performance as a function of the other player's performance feedback.

DISCUSSION

People learn about themselves from objective experience (Bandura, 1977), but their judgments are also deeply influenced by the social environment (Allport, 1924; Festinger, 1954). In our experiment, we show that both mechanisms occur when learning about the abilities of self and others. Subjects form estimates of how well they themselves and others do based on explicit performance feedback. However, these estimates are not separate but influence each other reciprocally. Subjects systematically overestimated their abilities when cooperating with a good partner compared to a bad one, and the reverse was true in competition. Also, others were estimated as more similar in ability to oneself in cooperation but dissimilar to oneself in competition. Such interdependence could be described as "anchoring" (Tversky and Kahneman, 1974) self and other judgments to each other in cooperation but as the reverse in competition. Two adjacent areas in medial prefrontal cortex, pgACC and area 9, track self and other performance, respectively. Area 9 moreover integrated multiple signals relating also to the self and the context, and it predicted the degree to which self and other abilities were estimated in an interdependent fashion.

PgACC signals tracked subjects' own performance. This area was previously found when subjects judged whether a given trait related to themselves (Denny et al., 2012; Kelley et al., 2002) and when mentalizing about oneself and similar other people (Mitchell et al., 2006) even when controlling for the effects of likeability (Mobbs et al., 2009). In our study, pgACC activity increased and decreased according to how well subjects thought they would perform in the experiment (*S-performance* signal; Figure 3). For subjects with a stronger *S-performance* signal in pgACC, performance history more strongly governed estimates of their own ability. Activity in other brain areas has been associated with action values, but our findings indicate that activity in pgACC reflects the assignment of ability, or a general value, to the self rather than the value of a particular choice (Figures S5B and S5C). Such representations in pgACC might be used to predict whether one is capable of succeeding in a given endeavor (Bandura, 1977) and as such might be subjectively perceived as rewarding in themselves. This idea is consistent with recent findings that regions in anterior cingulate cortex are related to the physical costs monkeys are willing to endure in order to obtain reward (Amemori and Graybiel, 2012). Moreover, such value assignments to the self (rather than value assignment to specific choices) may be altered in psychological syndromes such as depression (Murray et al., 2011). Note also that using predetermined and well-controlled performance feedback was critical for our investigation of feedback-guided ability learning as it decoupled feedback-guided learning about oneself from

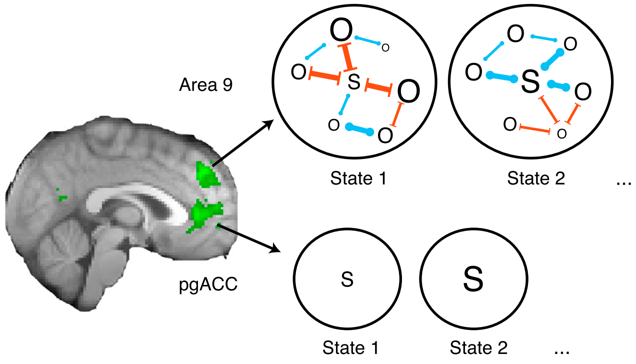

Figure 5. Self-Other Mergence and Interdependent Self and Other Representations in Area 9

In our study, pgACC holds a representation of oneself that is related to observing one's own performance (Figure 3). It may reflect the value of one's own actions for earning future reward or overcoming challenges. This ability estimate may be lower or higher depending on the state the individual is in (indicated by smaller and bigger "S"). In contrast, signals in dorsomedial area 9 suggest a different function. Area 9 holds a representation of the self, too; however, it is embedded in the cooperative or competitive context (Figure 3A-iii). In addition, it integrates information about the ability of others (Figure 3A-ii) and the social relationship one has with them (Figure S5B-iii). A neural circuit that integrates such information might be useful to facilitate coordination between oneself and others and allow, for instance, the pursuit of shared goals. It might also be used to flexibly compute one's status within one's social network as circumstances change (blue and red connections indicate changing alliances and rivalries, varying in strength). This would enable humans and animals to assign values to self and others based on the dynamics of their social relationship—something that very vividly guides choice in the complex dominance hierarchies of monkeys. If this is the case, then self-other mergence might accompany these neural processes in area 9 (Figure 3) occurring as a byproduct of relational self and other representations held by this brain region.

introspective (meta-cognitive) estimation of one's own abilities (Bahrami et al., 2010; De Martino et al., 2013).

In contrast to pgACC, area 9 signals were more complex. There were clear signals relating to representation of the other. For example area 9 was active at the time point that subjects made ratings of the relevant other and more specifically it tracked other-related performance estimates and prediction errors (Figures 3 and 4). Activity in area 9 has been found frequently in previous studies when trait judgments were made about other people (Denny et al., 2012) and mentalizing about dissimilar others (Mitchell et al., 2006). In our study there were, however, also signals relating to self and the social relationship with the other. Individual differences in area 9 activity indicated how much self/other information was used in a relational way, predicting SOM both toward the self and toward the other.

This sheds light on the function area 9 may have in social cognition. Self-other mergence might reflect difficulties that arise from tracking and assigning both self- and other-related information to the appropriate agent. In this sense, the effects we report are reminiscent of failures of credit assignment to the appropriate choice during reward-guided learning (Chau et al., 2015; Thorndike, 1911, 1933; Walton et al., 2010). However, self-other mergence may also be a side-product of relational computations in area 9 (Figure 5). In our experiment, the cooperative and competitive contexts create a social relationship between self and other that subjects need to be aware of. This meant that expectations of success in a trial were not a result of one's own anticipated performance alone, but they were also dependent on the predicted performance of another player as well. Taking into account the actions of oneself in concert with another person's actions is not a trivial matter (Tomasello et al., 2005), and it has been argued that coordinating actions between self and other often relies on implicit agreements (Misyak et al., 2014). It might be that self-other mergence indexes the relational representations that are used in many social situations where outcomes are the consequence of joint actions (Seo et al., 2014), possibly even if oneself is only a passive observer. From this perspective, when cooperating with a weak partner, the reduced chances of achieving a shared goal as a team might be carried over to impact negatively on one's judgment of oneself. Note, however, that the strength of the direction of influence of self-other mergence (self to other or other to self) may depend on the social situation and other constraints. Furthermore, area 9 may integrate self, other, and relational information to compute one's own position in a social network when an individual's status is a reflexion of its alliances (Schülke et al., 2010). In monkeys, area 9 is related to dominance (Neubert et al., 2015; Noonan et al., 2014; Sallet et al., 2011), and thus adoption of a place in a social hierarchy might reflect the operation of a relational mechanism that could be performed in area 9.

EXPERIMENTAL PROCEDURES

Subjects

Twenty-six subjects participated in the experiment. Two were excluded from data analysis due to excessive motion (final sample: 24 subjects; nine female; aged 19–31). All provided informed consent. The study was approved by the ethics committee of the University of Oxford (MSD-IDREC-C1-2013-133). Subjects received £35 as a show-up fee and a bonus based on task performance (range: £4.50–£11.80).

Experimental Design and Schedule

While lying in the MRI scanner, on each trial, subjects performed a minigame. They were led to believe that two other subjects played the same minigame simultaneously (Figure 1; Figures S1–S3). Each trial took place either in a cooperative or in a competitive social context. Subjects then made an engage/avoid decision. On some trials, the choice was between cooperating or refraining from cooperating, while in other trials the choice was between competing or refraining from competing. If subjects took the "avoid" choice, then that meant that they were simply awarded a small number of points (1.5 points) randomly distributed around zero (and they were informed that this was the case). However, if they took the "engage" decision in the cooperative context, then they opted to ally themselves with one of the other players (which one varied from trial to trial and was indicated by the experimenter on each trial and is subsequently referred to as the relevant other, O) to see whether together they could perform well enough for their average points to exceed a threshold level (which varied from trial to trial). If they did, they gained reward points on that trial, but if they fell short of the threshold they lost points. By contrast, if they took the "engage" decision in the competitive context, then the other player became an opponent. The difference between the subject's and opponent's performances then had to exceed a threshold (again the threshold was variable), and the payoff was proportional to this difference (i.e., a win, a loss or neither of the two). In summary, the social context was critical when decisions to engage were made. Reward outcomes for engage/avoid choices were determined by minigame performances of S, O, and a threshold that varied unpredictably from trial to trial.

$$Engage\ Payoff_{Competition} = (feedback_S - feedback_O) - threshold \quad \text{(Equation 1a)}$$

$$Engage\ Payoff_{Cooperation} = (feedback_S + feedback_O)/2 - threshold \quad \text{(Equation 1b)}$$

"Feedback" in Equations 1a and 1b refers to performance feedback. The timing of events within each trial is illustrated in Figure 1.

While the likely performance feedback for S and O could be estimated from performance feedback on previous trials, the threshold varied unpredictably from trial to trial and was used to dissociate reward expectation from performance expectation and to make sure that subjects did not make their choices before the beginning of the current trial. Subjects found the meaning of the thresholds intuitive when the task was being explained to them, and their task behavior confirmed that they had understood the task.

Subjects then also provided an estimate of their ability on each trial by rating the expected performance for themselves (S) and the relevant other (O) for the upcoming trial of the minigame (Figure S2B). The order of S and O ratings was randomized across trials. As explained above, although both of the two other players performed the minigame simultaneously, subjects were only paired (to compete or cooperate) with one of the other players (the relevant other, O). Therefore, only O, and not the third player, was relevant for a trial's engage/avoid decision. However, the identity of O switched between trials. On each trial, after the minigame, subjects received performance feedback about themselves as well as about the performances of the other two players (Figure S2A). As minigames, we designed two short reaction-time-based tasks. See Figure S3 for details on the minigames. The goal of the subjects in the experiment was to collect as many rewards (points) as possible, as these were translated into monetary reward at the end of the experiment.

For all three players, including the subjects themselves, performance feedback on every trial was predefined (Figure S1A). In other words, the feedback about performance was independent from subjects' actual performance in the minigames (see, however, "false-start trials" for a case of veridical performance feedback in the "Feedback" section of the Experimental Procedures and Figure S2C). This was necessary to control and match performance feedback between subjects as well as between subjects and the two other players. Subjects were told that the minigames had been tested on a larger sample of subjects and that performance feedback in the minigame reflected individual performance relative to that sample.

In the phases before and after the minigames, three scales ranging from 1 to 15 points were shown with the initials of the three players below. Performance feedback was displayed on these scales in the feedback phase. While the initials of the confederates were the same for all experimental sessions, the subjects' own initials were adjusted to be appropriate for each individual subject. The initials created a social frame for the experiment without using explicitly social cues such as faces.

The experimental schedule contained 136 minigame trials. The design was a 2(social context) × 2(partner) × 2(minigame) fully crossed design (17 trials per cell). This meant that a trial could be either cooperative or competitive (social context: cooperation or competition), the O could be either "player" 1 or 2 (O: Other1 or Other2), and, in each trial, subjects played one of two minigames (minigame: time task or color task). The trial type order was pseudorandom and the same for all subjects. Three subjects performed a marginally shortened version of the schedule. They did the first 116 trials of the schedule; however, the design was still fully crossed (14 trials per cell).

Several features of the experiment were counterbalanced independently across subjects to avoid confounds. The mapping between minigame (time task or color task), and associated performance feedback schedule was counterbalanced. Moreover, as the left/right sides of the buttons used to indicate the engage/avoid choice were fixed for each subject, they were counterbalanced across subjects. We also counterbalanced the screen location where initials of each confederate were displayed (left or right of subjects' initials, which were always in the middle), and association between confederates (whom the subjects had met before going into the scanner) and their performance feedback schedules.

Thus, in summary, all trials comprised an engage/avoid decision, two binary ratings (for S and O), a minigame phase (described in detail in Figure S3), and a feedback phase. Timing details for all phases (except minigame phases) are shown in Figure S2.

Experimental Procedures

The experimental procedure was precisely scripted and involved two experimenters, two confederates, and the radiographer to make subjects believe they would be playing an interactive game together with two other subjects. The same two confederates pretended to be the two other players for every subject. Details about the task instructions are presented in Figure S1B. The task in the MRI scanner took approximately 55 min. After functional and structural MRI sessions, subjects filled in two short questionnaires and were then fully debriefed about the experiment. No subject indicated any suspicion about false performance feedback or the identity of the confederates before debriefing. Supporting results from a debriefing questionnaire are shown in Figure S1C.

Note that we have several indications (and no counter-indications) that subjects' ability estimates were guided by the performance feedback and therefore that subjects found the feedback credible: the effects of past performance feedback on (1) *S-ability* and *O-ability* ratings, (2) on decisions to engage in cooperation/competition (both Figure 2), (3) on true minigame performance (Figure 4), and (4) subjects' self-reports of the feedback credibility in a debriefing interview, documented by a debriefing questionnaire (Figure S1).

Ability Ratings

As already mentioned subjects provided S and O ability ratings. For each rating, initially, a tick indicated a value on the performance scale (rating marker) and subjects indicated whether expected performance (for S or O as appropriate) would surpass or fall below the rating marker (Figure S2B). A positive rating (i.e., performance is expected to be above the rating marker) was made with one button, and a negative rating (i.e., performance is expected to be below the rating marker) was made with the other button. As performance feedback was always expressed in integers, the rating makers were always set between two integers (X.5 values). The rating marker was updated from trial to trial based on the rating choices for the respective player using a staircasing procedure to increase sensitivity of the ratings. A positive rating resulted in an increase of the rating marker's value by one point in the next trial of the same minigame for the given player; a negative rating resulted in a decrease by one point. The value of the rating marker on the first trial after the starter trials (see below) was based on the player's mean performance feedback in the starter trials.

Subjects received a small payoff for the accuracy of the ratings. To reduce incentives to perform badly on the minigames, negative ratings never yielded payoffs. For positive ratings, subjects won or lost 0.25 points depending on whether the subsequent performance feedback received surpassed or fell below the rating marker. Note that the magnitude of the rating payoff was insignificant compared to the payoff for the engage/avoid decision.

Feedback

Feedback was chunked together in three components that were presented in randomized order. The first component was the performance feedback for S and O, which was presented simultaneously with the information about the accuracy of the subjects' ratings (Figure S2B). The second component was the payoff of the engage/avoid decision. For this, a cue indicating the trial's choice appeared on the right side of the screen (Figure S2A) together with circles representing coins that were won (yellow circles with a plus sign) or lost (red circles with a minus sign). At the same time, only for engage choices, the performance feedback average (cooperation trials) or performance feedback difference (competition trials) was displayed on the scales on the right side of the screen. The third component was the performance feedback of the other player that was not the O (irrelevant other). The initials of this player were displayed in a different color, and the performance was irrelevant for any payoff. The three feedback components appeared in random order to control for sequence effects. The first component occurred after 1 s, the second component 1.25 s later, the third component another 1.25 s later, and the feedback phase ended after a further 4 s. Then, after 0.5- to 2.5-s inter-trial interval (ITI) with a blank screen, the next trial started.

Two types of trials deviated from the described structure. First, the first four trials of the experiment were "starter trials" (two with the time task, two with the

in color task). Those trials were for subjects to form initial ability estimates about the players. For this reason, in starter trials, there was no option to cooperate or compete, and no ability ratings were made. Second, for trials where subjects performed very badly in a minigame ("false starts"), the feedback phase was adjusted. The performance thresholds for false-start trials are discussed in Figure S3. In false-start trials, subjects received no performance feedback for themselves, but only for the other players (Figure S2C; Figure S6B shows the number of false-start trials per subject). The sole payoff for false-start trials was a loss of two points independent of subjects' ratings and engage/avoid choice. Subjects were instructed about this. It was explained that extremely bad performances would be detected by the computer running the experiment and discarded as false starts to sort out performance slips that were, for instance, due to inattentiveness and did not reflect a player's "true" performance. This procedure was used to make the pre-determined feedback in other trials more believable as the feedback in false-start trials was actually determined by true minigame performance. Note that subjects were also told during the instructions that there would be a special type of false-start trial if one of the other players performed very badly. However, this never happened in the experiment. Starter trials and the feedback phase of false-start trials were excluded from fMRI analysis.

Reinforcement Learning Model

See Supplemental Experimental Procedures 1 for details on the RL model.

Behavioral Analysis

See Supplemental Experimental Procedures 2 for details on behavioral data analysis.

Imaging Analyses

See Supplemental Experimental Procedures 3 and Figure S5 for details on MRI data acquisition and analysis.

SUPPLEMENTAL INFORMATION

Supplemental Information includes Supplemental Experimental Procedures, six figures, and one table and can be found with this article online at http://dx.doi.org/10.1016/j.neuron.2016.06.022.

AUTHOR CONTRIBUTIONS

M.K.W. and M.S.F.R. conceived the experiment. M.K.W., N.K., N.S.F., and M.S.F.R. designed the experiment. M.K.W. and N.N. collected the data. M.K.W. analyzed the data and wrote the manuscript. N.K., N.S.F., J.S., and M.S.F.R. provided expertise and feedback on data analysis and write-up; M.S.F.R. supervised the research.

ACKNOWLEDGMENTS

This work was funded by the Wellcome Trust (M.K.W.: 096589/Z/11/Z; M.F.S.R.: WT100973AIA; J.S.: 09275/Z/10/Z). N.K. is a Christ Church Junior Research Fellow. We thank Jim Everett, Felix Heise, and Magdalena Soukupova for their help in collecting the data, and Tim Behrens, Erie Boorman, Miriam Klein-Flügge, Robert Lorenz, David Meder, and Nadescha Trudel for helpful discussion.

Received: February 25, 2016
Revised: April 29, 2016
Accepted: June 13, 2016
Published: July 20, 2016

REFERENCES

Allport, F.H. (1924). The group fallacy in relation to social science. J. Abnorm. Soc. Psychol. *19*, 60–73.

Amemori, K., and Graybiel, A.M. (2012). Localized microstimulation of primate pregenual cingulate cortex induces negative decision-making. Nat. Neurosci. *15*, 776–785.

Amodio, D.M., and Frith, C.D. (2006). Meeting of minds: the medial frontal cortex and social cognition. Nat. Rev. Neurosci. *7*, 268–277.

Bahrami, B., Olsen, K., Latham, P.E., Roepstorff, A., Rees, G., and Frith, C.D. (2010). Optimally interacting minds. Science *329*, 1081–1085.

Bandura, A. (1977). Self-efficacy: toward a unifying theory of behavioral change. Psychol. Rev. *84*, 191–215.

Boorman, E.D., O'Doherty, J.P., Adolphs, R., and Rangel, A. (2013). The behavioral and neural mechanisms underlying the tracking of expertise. Neuron *80*, 1558–1571.

Boyd, R., and Richerson, P.J. (2009). Culture and the evolution of human cooperation. Philos. Trans. R. Soc. Lond. B Biol. Sci. *364*, 3281–3288.

Brewer, M.B. (1979). In-group bias in the minimal intergroup situation: a cognitive-motivational analysis. Psychol. Bull. *86*, 307–324.

Chang, S.W., Brent, L.J., Adams, G.K., Klein, J.T., Pearson, J.M., Watson, K.K., and Platt, M.L. (2013a). Neuroethology of primate social behavior. Proc. Natl. Acad. Sci. USA *110* (*Suppl 2*), 10387–10394.

Chang, S.W., Gariépy, J.F., and Platt, M.L. (2013b). Neuronal reference frames for social decisions in primate frontal cortex. Nat. Neurosci. *16*, 243–250.

Chau, B.K., Sallet, J., Papageorgiou, G.K., Noonan, M.P., Bell, A.H., Walton, M.E., and Rushworth, M.F. (2015). Contrasting Roles for Orbitofrontal Cortex and Amygdala in Credit Assignment and Learning in Macaques. Neuron *87*, 1106–1118.

Daw, N.D., Gershman, S.J., Seymour, B., Dayan, P., and Dolan, R.J. (2011). Model-based influences on humans' choices and striatal prediction errors. Neuron *69*, 1204–1215.

De Martino, B., Fleming, S.M., Garrett, N., and Dolan, R.J. (2013). Confidence in value-based choice. Nat. Neurosci. *16*, 105–110.

Denny, B.T., Kober, H., Wager, T.D., and Ochsner, K.N. (2012). A meta-analysis of functional neuroimaging studies of self- and other judgments reveals a spatial gradient for mentalizing in medial prefrontal cortex. J. Cogn. Neurosci. *24*, 1742–1752.

Festinger, L. (1954). A Theory of Social Comparison Processes. Hum. Relat. *7*, 117–140.

Fliessbach, K., Weber, B., Trautner, P., Dohmen, T., Sunde, U., Elger, C.E., and Falk, A. (2007). Social comparison affects reward-related brain activity in the human ventral striatum. Science *318*, 1305–1308.

Garvert, M.M., Moutoussis, M., Kurth-Nelson, Z., Behrens, T.E., and Dolan, R.J. (2015). Learning-induced plasticity in medial prefrontal cortex predicts preference malleability. Neuron *85*, 418–428.

Heider, F. (1958). The Psychology of Interpersonal Relations (John Wiley & Sons).

Izuma, K., and Adolphs, R. (2013). Social manipulation of preference in the human brain. Neuron *78*, 563–573.

Kelley, W.M., Macrae, C.N., Wyland, C.L., Caglar, S., Inati, S., and Heatherton, T.F. (2002). Finding the self? An event-related fMRI study. J. Cogn. Neurosci. *14*, 785–794.

Kerr, N.L., and Tindale, R.S. (2004). Group performance and decision making. Annu. Rev. Psychol. *55*, 623–655.

Krueger, J.I. (2010). From social projection to social behaviour. Eur. Rev. Soc. Psychol. *18*, 1–35.

Misyak, J.B., Melkonyan, T., Zeitoun, H., and Chater, N. (2014). Unwritten rules: virtual bargaining underpins social interaction, culture, and society. Trends Cogn. Sci. *18*, 512–519.

Mitchell, J.P., Macrae, C.N., and Banaji, M.R. (2006). Dissociable medial prefrontal contributions to judgments of similar and dissimilar others. Neuron *50*, 655–663.

Mobbs, D., Yu, R., Meyer, M., Passamonti, L., Seymour, B., Calder, A.J., Schweizer, S., Frith, C.D., and Dalgleish, T. (2009). A key role for similarity in vicarious reward. Science *324*, 900.

Murray, E.A., Wise, S.P., and Drevets, W.C. (2011). Localization of dysfunction in major depressive disorder: prefrontal cortex and amygdala. Biol. Psychiatry *69*, e43–e54.

Mussweiler, T. (2003). Comparison processes in social judgment: mechanisms and consequences. Psychol. Rev. *110*, 472–489.

Neubert, F.X., Mars, R.B., Sallet, J., and Rushworth, M.F. (2015). Connectivity reveals relationship of brain areas for reward-guided learning and decision making in human and monkey frontal cortex. Proc. Natl. Acad. Sci. USA *112*, E2695–E2704.

Nicolle, A., Klein-Flügge, M.C., Hunt, L.T., Vlaev, I., Dolan, R.J., and Behrens, T.E.J. (2012). An agent independent axis for executed and modeled choice in medial prefrontal cortex. Neuron *75*, 1114–1121.

Noonan, M.P., Sallet, J., Mars, R.B., Neubert, F.X., O'Reilly, J.X., Andersson, J.L., Mitchell, A.S., Bell, A.H., Miller, K.L., and Rushworth, M.F. (2014). A neural circuit covarying with social hierarchy in macaques. PLoS Biol. *12*, e1001940.

Ruff, C.C., and Fehr, E. (2014). The neurobiology of rewards and values in social decision making. Nat. Rev. Neurosci. *15*, 549–562.

Sallet, J., Mars, R.B., Noonan, M.P., Andersson, J.L., O'Reilly, J.X., Jbabdi, S., Croxson, P.L., Jenkinson, M., Miller, K.L., and Rushworth, M.F. (2011). Social network size affects neural circuits in macaques. Science *334*, 697–700.

Saxe, R. (2006). Uniquely human social cognition. Curr. Opin. Neurobiol. *16*, 235–239.

Schülke, O., Bhagavatula, J., Vigilant, L., and Ostner, J. (2010). Social bonds enhance reproductive success in male macaques. Curr. Biol. *20*, 2207–2210.

Seo, H., Cai, X., Donahue, C.H., and Lee, D. (2014). Neural correlates of strategic reasoning during competitive games. Science *346*, 340–343.

Sul, S., Tobler, P.N., Hein, G., Leiberg, S., Jung, D., Fehr, E., and Kim, H. (2015). Spatial gradient in value representation along the medial prefrontal cortex reflects individual differences in prosociality. Proc. Natl. Acad. Sci. USA *112*, 7851–7856.

Sutton, R.S., and Barto, A.G. (1998). Reinforcement learning: an introduction, *Volume 28* (MIT Press).

Suzuki, S., Harasawa, N., Ueno, K., Gardner, J.L., Ichinohe, N., Haruno, M., Cheng, K., and Nakahara, H. (2012). Learning to simulate others' decisions. Neuron *74*, 1125–1137.

Thorndike, E.L. (1911). Animal Intelligence; Experimental Studies (Macmillan).

Thorndike, E.L. (1933). A Proof of the Law of Effect. Science *77*, 173–175.

Toma, C., Yzerbyt, V., and Corneille, O. (2010). Anticipated cooperation vs. competition moderates interpersonal projection. J. Exp. Soc. Psychol. *46*, 375–381.

Tomasello, M., Carpenter, M., Call, J., Behne, T., and Moll, H. (2005). Understanding and sharing intentions: the origins of cultural cognition. Behav. Brain Sci. *28*, 675–691, discussion 691–735.

Tversky, A., and Kahneman, D. (1974). Judgment under Uncertainty: Heuristics and Biases. Science *185*, 1124–1131.

Walton, M.E.M., Behrens, T.E.J.T., Buckley, M.J., Rudebeck, P.H., and Rushworth, M.F. (2010). Separable learning systems in the macaque brain and the role of orbitofrontal cortex in contingent learning. Neuron *65*, 927–939.

Wang, F., Zhu, J., Zhu, H., Zhang, Q., Lin, Z., and Hu, H. (2011). Bidirectional control of social hierarchy by synaptic efficacy in medial prefrontal cortex. Science *334*, 693–697.

Zink, C.F., Tong, Y., Chen, Q., Bassett, D.S., Stein, J.L., and Meyer-Lindenberg, A. (2008). Know your place: neural processing of social hierarchy in humans. Neuron *58*, 273–283.

Neuron, Volume *91*

Supplemental Information

Self-Other Mergence in the Frontal Cortex

during Cooperation and Competition

Marco K. Wittmann, Nils Kolling, Nadira S. Faber, Jacqueline Scholl, Natalie Nelissen, and Matthew F.S. Rushworth

Supplemental data items:

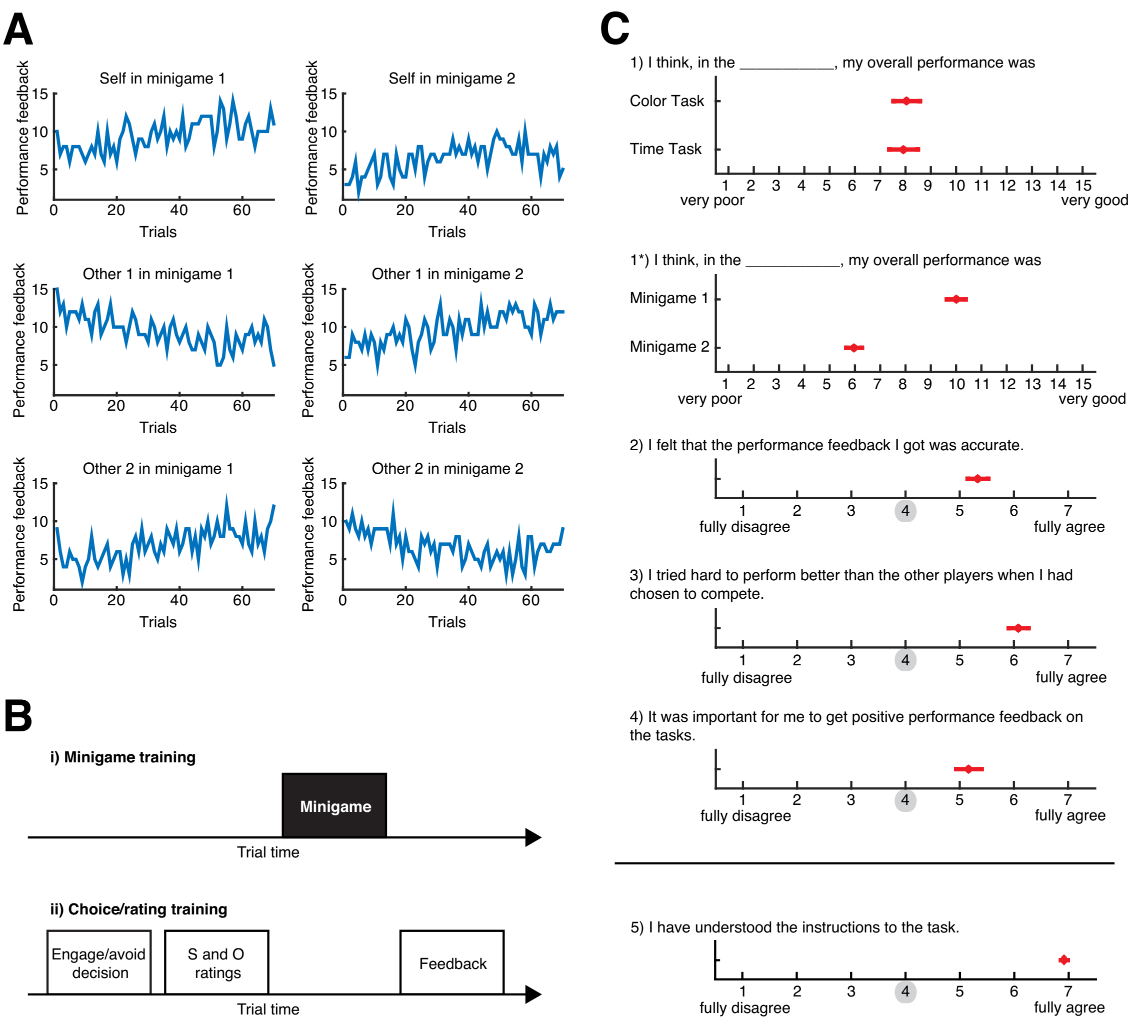

Figure S1 (relating to Figure 1)

Performance feedback schedules, instruction and debriefing questionnaire. (A) Performance feedback was predetermined to guarantee that self and relevant other (S and O, respectively) performance feedback allowed a meaningful data analysis. The performance feedback for S and O was similar in mean and variance. However, very bad minigame performance did lead to veridical performance feedback (see false start trials in Experimental Procedures). Subjects performed one of two minigames on each trial and S performance feedback was more positive in one minigame compared to the other minigame (counterbalanced across subjects), while variance in S performance feedback was similarly small in both minigames. This was done to sample a wide range of S performance feedback while still keeping S performance feedback relatively stable within each minigame. The same logic applied to the performance feedback schedules of the two other players. In short, mean, variance and slow drifts of performance feedback were balanced for self and others to ensure that differential behavioral and neural effects were not driven by schedule differences. The relevant other (O) in a trial was Other 1 in half of the trials and Other 2 in the other half. Trials with minigame 1 and minigame 2 were pseudorandomly interleaved. **(B)** The experimental procedure and instructions followed a precise schedule. Two confederates arrived in the laboratory at a similar time as the subjects. Subjects had been told in advance that the experiment investigates learning and decision making in social situations and that two other naïve subjects would participate in the same experiment. After a short introduction, subjects were separated from the confederates and instructed about the experimental task. They were told that they would be playing an interactive game together with the confederates, who would play the game from computers outside the scanner room. A faked lottery was

used to determine who of the three alleged subjects went in the scanner. For approximately 45 minutes, subjects were instructed on the minigames, the ratings and the engage/avoid decisions and performed example trials. The instructions were designed such that actual performance learning would only take place in the subsequent experiment to maximize learning effects in the scanner. To still guarantee familiarity with the minigames, a written explanation of the two minigames was complemented with a short practice session, in which subjects performed 5 trials of each minigame (B-i). During those trials, the experimenter was present and made sure that subjects understood the minigames. No explicit performance feedback was given on those trials to avoid performance learning. To guarantee that subjects understood the logic of the engage/avoid decision and the ratings, subjects performed 16 example trials that did not include minigames, but instead a placeholder screen (B-ii). This allowed subjects to adjust to the trial events and experience the reward outcomes of ratings and decisions. Importantly, the performance feedback on those trials followed no across-trial contingencies and consisted mostly of the highest or lowest performance feedback for the players. This was done to make these example trials very different from the trials experienced in the main experiment. Although subjects could not learn anything during those trials and therefore could make no well-grounded decisions, they were asked to invent and verbalize reasons for their ratings and decisions so that the experimenter could make sure that they understood their logic (e.g. "This is a cooperate trial. I press the "engage" button, because although the threshold is high, I think we will perform very well. I rate myself and the other one positively, because I think we will both perform well..."). In sum, subjects practiced all aspects of the experimental task, but in such a manner that they could not yet learn about their performance. Subjects were told that their goal in the experiment was to collect as many points as possible and that points would be translated into monetary reward at the end of the experiment. It was emphasized that points could be earned by making good decisions and by providing accurate ratings of performance. Despite the substantial time needed for a thorough instruction, most subjects found the task intuitive and both the behavioral data acquired in the experiment (see main text) and a post-experiment questionnaire confirmed that they understood the task. See question 5 in panel C: 22 subjects gave a 7 out of 7 rating for the question "I have understood the instructions to the task" (the remaining 2 subjects gave 6 out of 7). **(C)** After the experiment, subjects filled in a debriefing questionnaire with several questions that they rated on scales which were anchored for highest and lowest performance scores (see figure). These data showed that across the whole sample, subjects did not rate their overall performance in the two minigames differently (item 1; paired t-test: t_{23}=0.13; p=0.9;). This was expected as we counterbalanced across subjects whether time task or color task was used as minigame 1 which had higher performance feedback on average compared to minigame 2 (see upper left panel in (A); for 11 of 24 subjects the time task was used as minigame 1). Note also that there was no confound in the ordering of minigames because trials of minigame 1 and minigame 2 were interleaved across the whole experiment. When we retrospectively recoded item 1 with respect to minigame 1 and minigame 2, we found that subjects rated themselves better in the minigame where they received better performance feedback (item 1*; t_{23}=7.72; $p=7.7*10^{-8}$). Note that the performance scores for item 1 and 1* are very similar to the actual average performance feedback (see panel (A)) indicating further that subjects learned from the performance feedback. To confirm the believability of the performance feedback, we asked subject whether they felt that the performance feedback was accurate. If subjects experienced a mismatch between their subjective experience and the performance feedback they received, this would have been indicated by “disagree” ratings. We found slight agreement, however, indicating that subjects were unable to tell the inaccuracy of the performance feedback from their subjective experience. Additional suspicion checks asked whether they tried to perform better than O in competition (indicating that they believed that they could gain positive performance feedback by trying hard; item 3) and if positive performance feedback was valuable for them (item 4). In all cases (items 2,3,4) subjects tended to agree rather than disagree (average scores higher than scale point 4). This suggests that subjects indeed experienced the performance feedback as a consequence of their actions in the minigames. Note, importantly, that in addition to the debriefing questionnaire, a debriefing interview took place for every subject with additional suspicion checks. As we handed out this questionnaire before debriefing, to avoid biased responses, we could only indirectly ask if subjects had suspicions about the deception used. Therefore, we conducted debriefing interviews to fully disclose the purpose and method of the experiment to subjects, but also to confirm that all subjects, without exception, indicated that they believed the false performance feedback and had no doubts about the identity of the confederates. (error bars are mean +/-SEM across subjects).

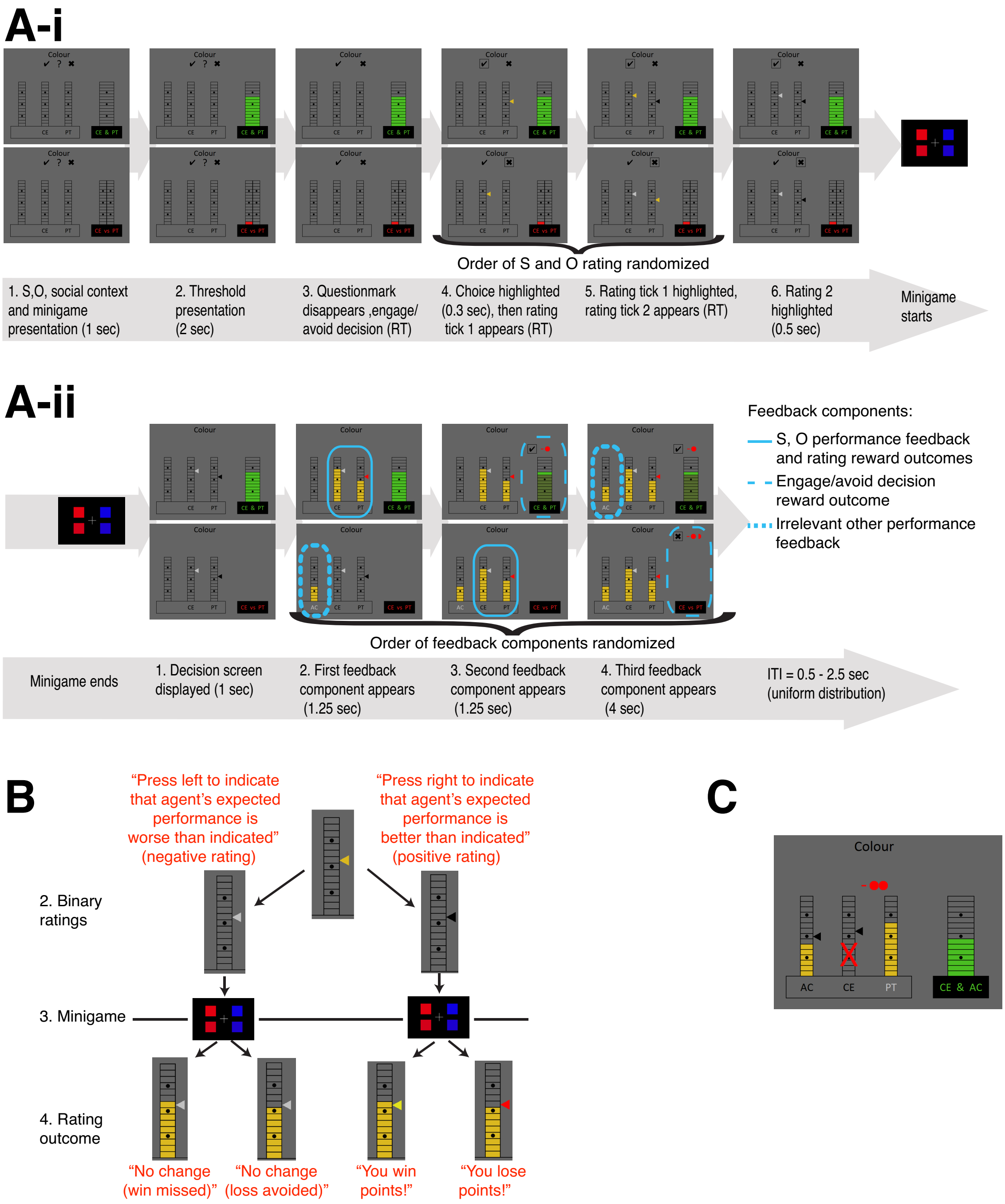

Figure S2 (relating to Figure 1)

Trial structure. (A) For an example trial, we show the first part of the trial in which the social context (competition or cooperation) was revealed, participants made the engage/avoid decision, and provided self (S) and relevant other (O) ratings (i). The feedback phase is shown of the same example trial (in ii). For illustration purposes, the same performance feedback is shown in a cooperative context (upper rows) and a competitive context (lower rows). Note that timing of the events are shown in brackets and RT (reaction time) means that the respective step only ends when the subject makes a response. **(A-i)** At the beginning of a trial S (initials of the subject), O (initials of one of the two other players relevant for the current trial), social context (cooperation or competition) and type of upcoming minigame ("Colour" or "Time") is presented. The initials of the irrelevant other player are not shown and the social context is color-coded indicating also the threshold of the current trial. In these examples, the choices made (see highlighted tick and cross in step 4) are to engage in cooperation (indicating the expectation that S and O will, on average, perform better than the threshold of 10) and to avoid competition (indicating that the subject does not expect to perform at least one point better than the O).

After the engage/avoid choices, binary ratings of S and O take place (step 4 to 6) in randomized order (in these examples, O rating first in cooperation and S rating first in competition). The ratings shown here indicate that the subject expects himself or herself to perform worse than 10.5, while the O is expected to perform better than 8.5 (see panel (B) for color coding of rating ticks). Note that these ratings are consistent with the engage/avoid choices shown in the two examples. **(A-ii)** In the feedback phase, the previous screen from before the minigame reappears. However, the right side of the screen showing the cooperation/competition threshold is occluded if a subject had chosen to avoid cooperation/competition in the previous decision phase of the trial. This means that in this example trial the threshold is only shown again in the cooperate trial (because the subject actually decided to cooperate) and not in the compete trial (because in this example trial the subject refrained from competing). In other words, the repeated presentation of the threshold is not a feature of the social context, but entirely a consequence of the engage/avoid choice made. Subsequently, three feedback components appear in randomized order to control for sequence effects (see legend on the right hand side). In cooperation, the choice payoff is -1, because the average performance is 9 while the threshold is 10. In competition, the choice payoff is -1.5 which is due to chance (payoff from avoid choices is +1.5 or -1.5 with a 50/50 probability) and independent of performance feedback. Note that the subject would have earned a payoff of 3 had the engage choice been taken (performance feedback difference of +4 minus threshold of +1). See panel (B) for an explanation of the rating payoff. Note that, overall, the magnitude of the rating payoff is marginal compared to the engage/avoid choice payoff. **(B)** Rationale of binary ratings. Before the minigame, subjects indicated for S and O either a positive or a negative rating reflecting the expectation that the player would surpass or fall below a given rating marker. The color change of the rating marker was indicative of the choice made. The rating marker turned black for a positive rating and grey for a negative rating. A positive rating led to a win or loss of 0.25 points depending on subsequent performance feedback. A negative rating led to no change in the points count independent of performance feedback. Therefore, making a correct negative rating was associated with a benefit of avoiding losing points while making a correct positive rating was associated with a benefit of winning 0.25 points. Note that in panel (A-ii), the performance and rating feedback indicate an incorrect negative rating (missed win) for S and an incorrect positive rating (loss) for O. Red text in quotes is taken from the subjects' instructions where a similar illustration was used. (**C)** The screenshot shows the feedback screen of a false start trial. In false start trials, the true performance of the subject in the minigame was below a predetermined threshold for acceptable performances. False start trials were a case of veridical performance feedback to ensure that performance feedback in general was believable. The feedback phase in false start trials was not analyzed and no prediction error for S was calculated on those trials (reward prediction error and prediction error for others were calculated as normal). Subjects were instructed about false start trials.

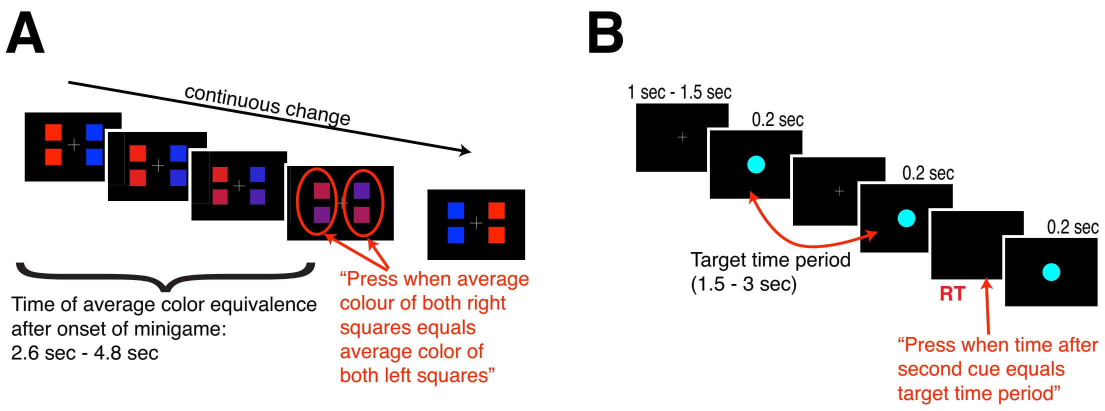

Figure S3 (relating to Figure 1)

Minigame description. We used two minigames in the experiment labeled "time task" and "colour task". The minigames were necessary prerequisites to administer performance feedback on every trial. They were short reaction-time based tasks and we designed them to be relatively non-transparent in the sense that the subjective experience of performing the minigames was not very informative for estimating one's ability compared to the explicit performance feedback that was given to the subjects. Also, we varied the timing parameters of the minigames to make it harder to compare true performance across different trials of a minigame. Lastly, we designed both minigames so that they appeared to be measuring different skills to make it plausible for subjects that performance levels in one task were not predictive of performance level in the other task. Each minigame was performed with one response button press with the right index finger. **(A)** The color task is loosely based on perceptual decision making tasks (Michael et al., 2014). Two pairs of squares, one pair red on the left, one pair blue on the right of a fixation cross, initially appear on screen. Then, the squares gradually change color over several seconds until the pairs have reversed their color. The color change occurs at an uneven rate. Subjects were asked to press a button when both pairs of squares had the same average color. The average colors were defined in RGB space as:

$$Color_{left} = [c_{red} \quad 0 \quad 1 - c_{red}]$$

$$Color_{right} = [c_{blue} \quad 0 \quad 1 - c_{blue}]$$

The colors were controlled by two parameters c_{red} and c_{blue}. For the left, initially red ([1 0 0] in RGB space) side, the parameter c_{red} decreased linearly from 1 to 0 over a run of the minigame. Vice versa, for the right, initially blue side ([0 0 1] in RGB space), the parameter c_{blue} increased linearly from 0 to 1 over a run of the minigame. The optimal time to press was when c_{red} and c_{blue} had the same value (color equivalence point), which happened only once as both parameters changed in opposing directions. The time of color equivalence was set to occur at a randomly picked time point drawn from a uniform distribution between 2.5 seconds and 4.7 seconds after onset of the color change. The c-values at that time were set to have a random value ranging from 0.25 to 0.75, also drawn from a uniform distribution. This meant the color equivalence point could lie rather towards the pure red or the pure blue side (a value of 0.5 indicates an even mix). Note that c_{red} and c_{blue} characterize the average color of the two left and two right squares, respectively. Hence, c_{red} (and c_{blue} analogously) was subdivided in c_{red1} and c_{red2} by multiplying c_{red} with a scaling parameter "colscale", which was randomly picked for each run of the minigame and ranged between 0.5 and 1.5 (c_{red1} = c_{red} x colscale; c_{red2} = c_{red} x (2-colscale)). In other words, c_{red} subdivided so that it was always the average color of c_{red1} and c_{red2}. Performance on this task was calculated as:

$$Performance_{ColorTask} = (c_{blue} - c_{red}) \times 100$$

Optimal performance was represented by a performance value of 0 indicating a color match and the performance values represented deviation from color equivalence in percent. Negative performance values indicate a response that was too quick (the point of equal c values had not yet been reached

when the subject responded) and positive performance values indicated a response that was too slow (c values are past the point of equality). If absolute performance values exceeded 60, trials were classified as false starts. This threshold was based on pilot experiments. Note again, that except for false start trials, the performance feedback was unrelated to the performance measure explained here. **(B)** In the time task subjects had to replicate a given time gap between two short occurrences of a blue dot on the screen (target time). The target time was randomly picked for each trial from a uniform distribution and ranged between 1.5 and 3 seconds. Subjects were instructed to press when the time elapsed after the second dot appearance was equal to the target time. The response time was the time from the second dot appearance to the button press. Therefore, performance on the task was calculated as:

$$Performance_{TimeTask} = (response\ time / target\ time) \times 100 - 100$$

As in the color task, optimal performance on the time task was represented by a performance value of 0 and the performance values represented deviation from the optimal response time in percent. Positive performance values indicated a response that was too slow and negative performance values indicated a response that was too quick. If absolute performance values exceeded 70, trials were classified as false starts. This threshold was based on pilot experiments.
Red text in (A) and (B) is taken from the subjects' instructions where a similar illustration was used. (RT, reaction time).

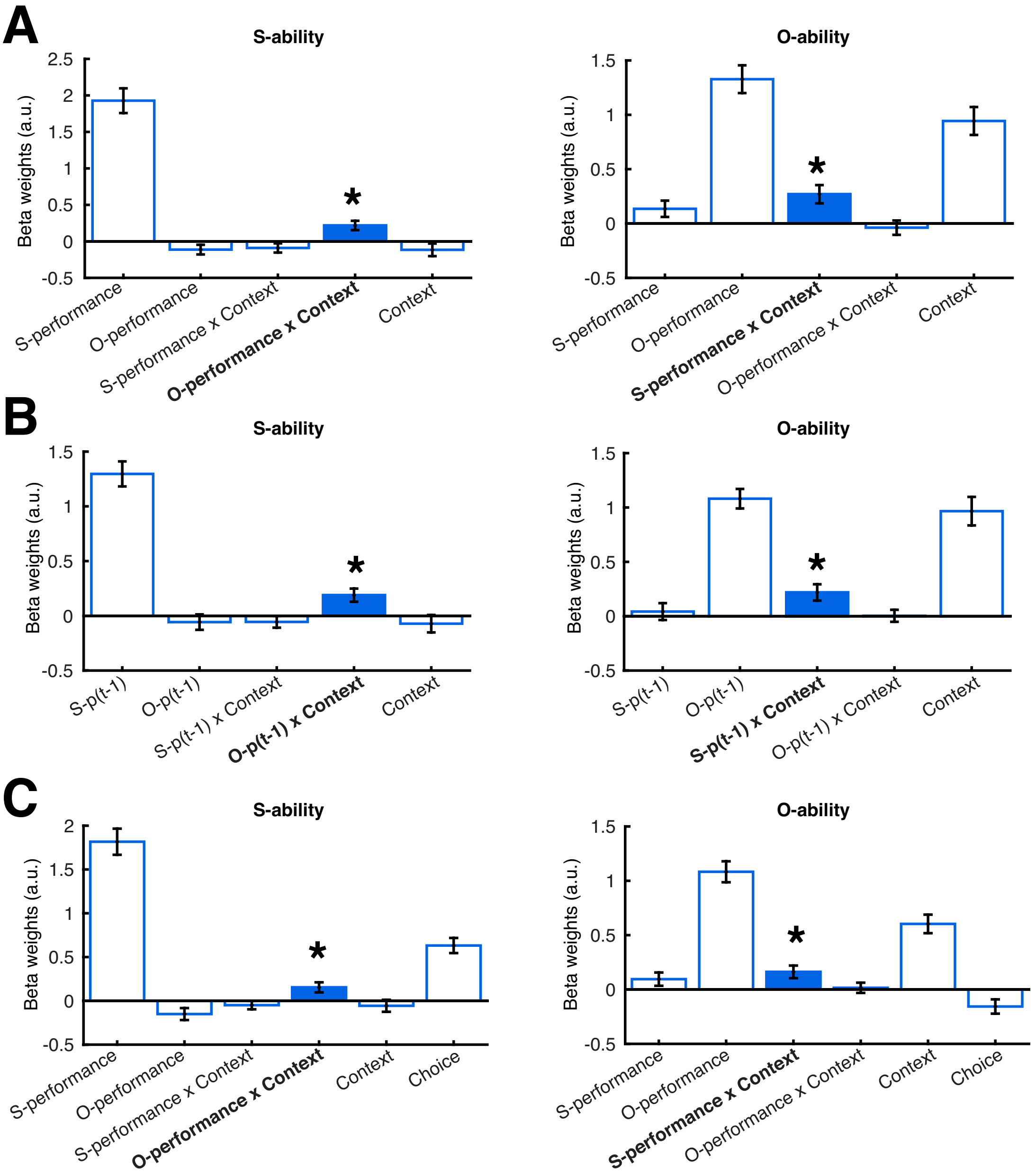

Figure S4 (relating to Figure 2)

Behavioral GLMs on the self ratings (*S-ability*, left panels) and relevant other ratings (*O-ability*, right panels) for engage trials (panels A and B) and all trials (C). For clarity, filled blue bars highlight self-other-mergence interaction (SOM_{int}) effects. We calculated the GLMs shown in Figure 2B-ii,C-ii (rating GLM2a, Supplemental Experimental Procedures 2) separately over compete and cooperate trials. For these GLMs here (rating GLM2b, Supplemental Experimental Procedures 2), however, the difference in effects of the observed performance history between cooperate and compete trials was coded as an interaction effect (*S-performance x Context* and *O-performance x Context*, respectively) such that a positive interaction reflects stronger influence of *S-performance/O-performance* for cooperate compared to compete trials. **(A)** We found significant SOM_{int} for *S-ability* (t-test against zero; *O-performance x Context* effect: t_{23}=3.39; p=0.0025) and O-ability (t-test against zero; *S-performance x Context*: t_{23}=3.21; p=0.0039). The results of these two significance tests are shown in the main text in Figure 2B-ii and Figure 2C-ii, respectively. In other words, we used the GLMs shown in panel (A) for significance testing of the *S-performance* and *O-performance* effects binned by social context that are shown in Figure 2B-ii and Figure 2C-ii. Similarly, the beta weights of the relevant effects in panel (A) were correlated with brain signals across subjects in Figure 3B-i,ii,iii. In detail, this was the behavioral effect of *S-performance* on *S-ability* for Figure 3B-i, the effect of *O-performance* on *S-ability* for Figure 3B-ii (also referred to as SOM_{main}(O-->S), because it is the main

effect of the relevant other's performance independent of social context) and the effect of *S-performance x Context* on *O-ability* for Figure 3B-iii. There is also a main effect of Context on *O-ability*, suggesting that the other is evaluated better in cooperation than in competition, mirroring research demonstrating a general evaluation bias in favour of cooperating in-group members compared to competing out-group members (Brewer, 1979). **(B)** Control GLMs for results presented in panel (A) (which used rating GLM2b) that do not use parameters derived from a reinforcement learning (RL; Supplemental Experimental Procedures 1) model. These GLMs used the same regressors as in panel (A), except that the summary terms for performance history, *S-performance* and *O-performance*, were replaced with the most recent piece of performance feedback that was received for that player (S-p(t-1) and O-p(t-1), respectively). As in panel (A), this control GLM is calculated using engage trials only. Interaction effects with social context were calculated as in panel (A) resulting in significantly positive SOM_{int} effects for *S-ability* (*O-p(t-1) x Context*; t_{23}=3.13; p=0.0047) and *O-ability* (*S-p(t-1) x Context*; t_{23}=2.92; p=0.0078). This demonstrates that self-other-mergence effects for both *S-ability* and *O-ability* did not rely on specific parameters used by our RL model for summarizing performance history. **(C)** Control GLMs for results in panel (A) (which used rating GLM2b) showing significant SOM_{int} when the analysis is not restricted to engage trials (as in panels (A) and (B)) but also includes trials on which subjects avoided cooperation/competition. We repeated rating GLM2b but extended the analysis over both engage and avoid choice trials. Including avoid choices is problematic because O performance is irrelevant to the participants' outcomes on these trials and so any SOM effects are expected to be weaker. To account for choice, we added a binary choice regressor (normalized to a mean of zero and standard deviation of 1, positive values for engage, negative values for avoid trials). The SOM on *S-ability* (t_{23}=2.67; p=0.0138) and *O-ability* (t_{23}=2.79; p=0.0103) still remained significant. Note that, among the points raised in the main text, this inability to fully distinguish own competence from the competence of others might also help understanding why people who cooperate in a group often are not able to make good use of the individual knowledge they could contribute (Stasser and Titus, 1985) and, in general, why performance in groups is so hard to predict from individual performance (Faber et al., 2015). (error bars are mean +/-SEM; *, $p < 0.05$)

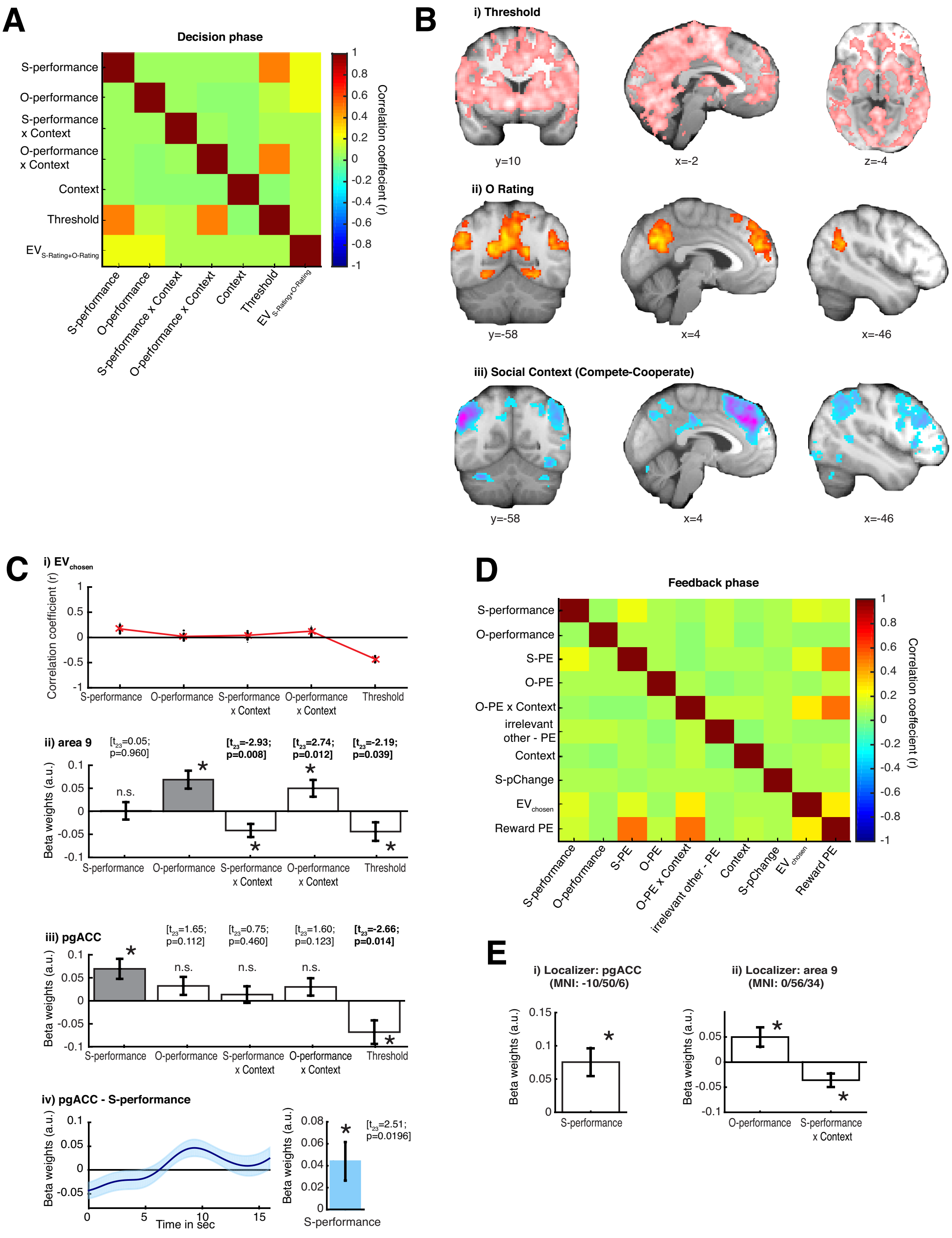

Figure S5 (relating to Figure 3)

Supplemental fMRI results. (A) Correlation matrix of regressors of interest at the time of the decision over all trials. **(B-i)** Cluster-corrected negative effects of threshold at the time of the decision. The threshold had widespread negative effects, which in medial prefrontal cortex centered on the ventromedial prefrontal cortex (vmPFC) and not pgACC or area 9; the threshold-related activity can be considered as activity related to the decrement in reward expectation on the current trial caused by the presence of the threshold (in other words the cost of the current specific choice) rather than to *S-performance* or *O-performance*. **(B-ii,iii)** At the time when the rating of the relevant other was made, we found cluster-corrected positive effect (main effect of O rating, as opposed to a parametric contrast) in several brain regions typically associated with "theory of mind"-type aspects of social cognition (Saxe, 2006). These regions include dorsomedial prefrontal cortex, bilateral temporoparietal junction

and precuneus (ii). The effect of the social context (compete-cooperate) at the time of the decision had widespread cluster-corrected positive effects (iii). The regions include again dorsomedial prefrontal cortex and bilateral temporoparietal junction. Although the activation was widespread, the peak of activation in dorsomedial prefrontal cortex was, just as in the case of the "theory of mind" activations presented in ii, in area 9 as identified by *O-performance*. (**C-i),** Single subjects (small black dots) and median correlations (red line) between regressors of interest (from panel (A)) and EV_{chosen} (as defined in equation 10, Supplemental Experimental Procedures 1). We designed the experiment so that the correlations between EV_{chosen} and the expected performance of the players (as well as the corresponding interactions by social context) were as small as possible. However expected reward is still linked conceptually to *S-performance*, *O-performance x Context* and in particular the threshold, as the reward outcome of the engage/avoid decision was a linear combination of performance feedback S, performance feedback O (positive relation in cooperation, negative relation in competition) and the threshold. **(C-ii,iii)** Effects of regressors of interest for area 9 and pgACC. Grey bars indicate contrasts used to identify the ROI. The significance stars on these effects denote the statistical significance from analyses conducted at the whole-brain level; these effects were not again tested for significance in the ROI (to avoid "double dipping"). Note that the interaction term with social context is coded in a way that positive effects mean stronger signals for cooperate compared to compete trials (interaction uses "1" for cooperation, "-1" for competition). In Figure 3 of the main manuscript, the interaction term *S-performance x Context* is coded the other way around in the fMRI analysis shown, so that positive signals indicate stronger responses in compete compared to cooperate trials. This was done for visualization purposes only. We used a repeated measures analysis of variance to formally examine whether performance related brain signals were indeed different in pgACC compared to area 9 (Nieuwenhuis et al., 2011). For this, we first used leave-one-out procedures on the whole brain level to make sure that we performed this analysis in an unbiased way. With respect to the pgACC ROI, we identified the peak voxel for *S-performance* effects based on the whole group of subjects except one left out subject. We repeated this for all subjects. Thus, we were able to conduct ROI analyses without selection biases, as each subject's brain signals did not contribute to the ROI that was selected for that subject. In the same way, we identified the area 9 ROI using *O-performance*. We used a 2 [brain region] x 4 [performance signal] repeated measures ANOVA to compare the performance-related brain signals in both areas. The ANOVA included all four agent-specific signals that we investigated at the time of the decision (*S-performance, O-performance, S-performance x Context, O-performance x Context*) and compared their neural effect sizes for area 9 and pgACC. As expected, we found a highly significant interaction effect ($F_{3,69}$=6.7; p=0.0005). Main effects were absent (both p>0.08). **(C-iv)** Analysis of *S-performance* activity in pgACC in relation to EV_{chosen}. As *S-performance* is conceptually related EV_{chosen} (better *S-performance* leads to higher payoff in cooperation and competition) compared to *O-performance* (better *O-performance* leads to higher payoff in cooperation but to lower payoff in competition), we went on to analyze more formally if *S-performance* signals in pgACC could be reduced to EV_{chosen}.
First, we used again a leave-one-out procedure to determine individual pgACC ROIs using *S-performance* (see C-ii,iii).Within these ROIs, we conducted a time course analysis (Supplemental Experimental Procedures, section 3.3) using EV_{chosen} as the only regressor. We reapplied our original GLM (fMRI GLM for decision phase) to the residuals of this time course. In other words, we conducted our GLM of interest on a timecourse where the effects of EV_{chosen} had been partialled out. The effect of *S-performance* in this GLM is shown in panel (C-iv) as a time course analysis (left) and as a bar chart analysis (t_{23}=2.51; p=0.0196; right, Supplemental Experimental Procedures section 3.3). In sum, *S-performance* effects in pgACC persist after partialling out EV_{chosen} and therefore cannot be explained by the expected reward of the choice alone.

Note that the relationships between brain signals in pgACC and area 9 and behavior shown in Figure 3B of the main manuscript were specific to the respective brain area. This meant that any *S-performance* signal in area 9, regardless of its significance (in fact such signals were also non-significant), did not correlate with the effect of S-*performance* on *S-ability* (r=-0.27; p=0.20). Similarly, the neural signals that correlated with behavior in area 9, when measured in pgACC (again regardless of their significance), did not correlate with the behavioral variables shown in Figure 3B-ii,iii. In detail, any *O-performance* signal in pgACC did not predict the influence of *O-performance* on *S-ability* (r=-0.24; p=0.26) and *S-performance x Context* did not predict SOM_{int}(S-->O) (r=-0.02; p=0.91). Note that panels (ii) and (iii) show that none of these three signals was significant in the other area over all trials. Moreover, the correlation shown for pgACC was specific for the "rational" influence of *S-performance* on *S-ability* and did not occur with the "irrational" effect of *S-performance* on *O-ability* (r=0.03; p=0.88). Analogously, the behavioral correlation associated with area 9's *O-performance* signal was specific to the case of self-other-mergence (correlation of brain signal with *O-*

performance effect on *O-ability*: r=0.07; p=0.74) and so was the area 9 *S-performance x Context* effect specific to the case of self-other-mergence (correlation of brain signal with *S-performance x Context* effect on *S-ability:* r=-0.04; p=0.85).

(D) Correlation matrix of regressors of interest at the time of feedback (Supplemental Experimental Procedures, section 3.3, Figure 4C) over all trials. **(E)** Supplemental control analysis for self and other related signals in pgACC and area 9. We repeated our ROI analysis (similar to panel C-i,ii in this figure) for two independent ROIs. A recent meta-analysis of fMRI studies investigated trait judgments about self and others (Denny et al., 2012) and identified brain regions in medial frontal cortex where self-related judgments show increased BOLD activity compared to other-related judgments (self>other) and vice versa (self<other). The two regions correspond to our regions pgACC (i) and area 9 (ii), respectively. We replicated our effects of interest shown in Figure 3A for ROIs centered on the peak coordinates of these clusters. In detail, we again found a significant effect of *S-performance* in pgACC (t_{23}=3.61, p=0.002), and effects of *O-performance* (t_{23}=2.56; p=0.018) and *S-performance x Context* (t_{23}=-2.67; p=0.014) in area 9. Therefore, our results are not dependent on using a specific ROI location and they link up with the results of previous studies investigating similar concepts. Note that, as in panel C, the *S-performance x Context* effect is sign flipped compared to Figure 3 for ease of visualization in the main text. (error bars are mean +/-SEM; *, $p < 0.05$)

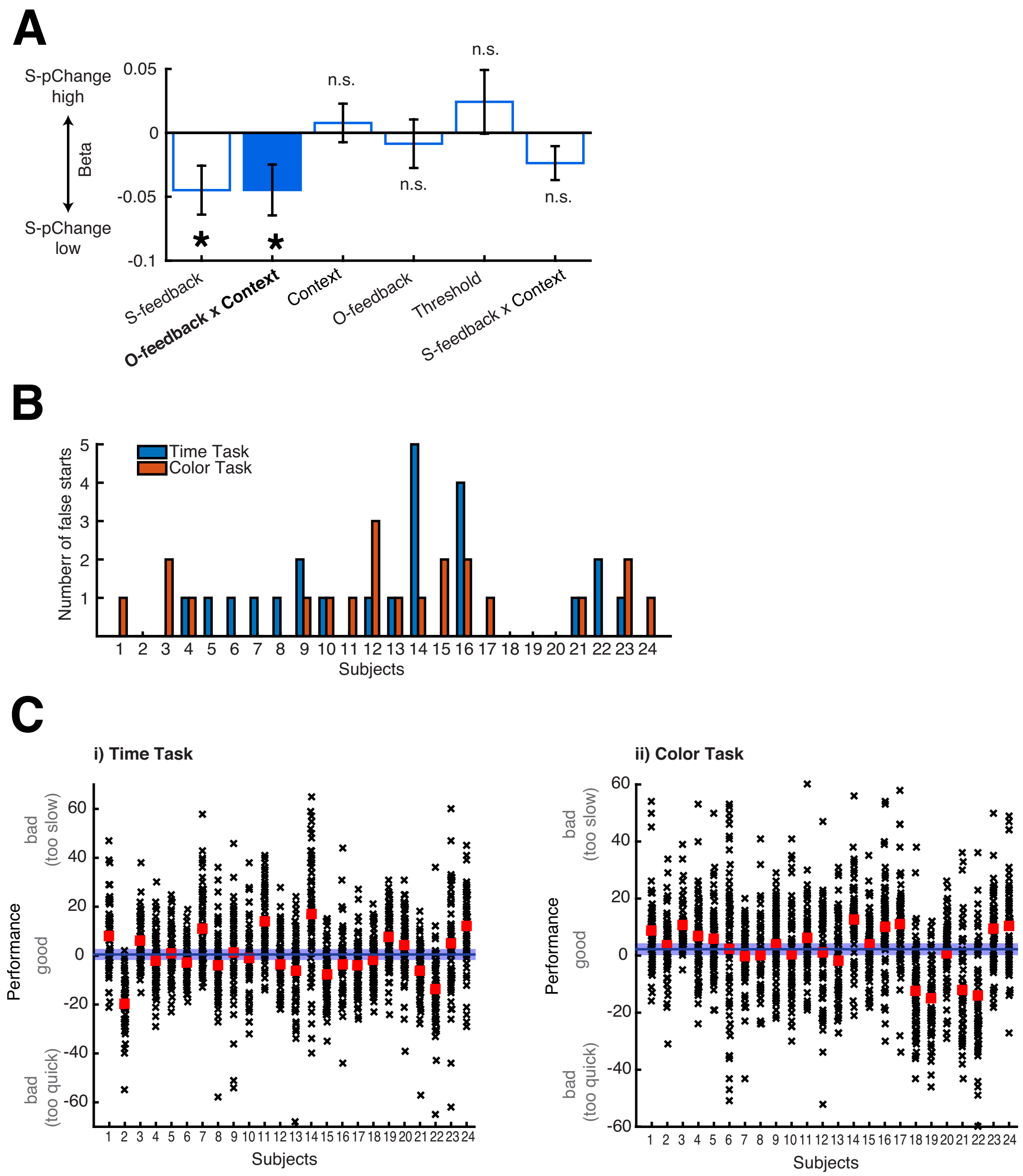

Figure S6 (relating to Figure 4)

Supplemental analysis of S-pChange and minigame performance. (A) Linear regression on S-pChange (S-pChange GLM1b, Supplemental Experimental Procedures 2). S-pChange was calculated within each minigame. This means that S-pChange reflects the absolute change in performance from a trial to the next trial of the same minigame; not across minigames. The filled blue bar indicates the SOM related effect. S-pChange from the current to the next trial was higher when subjects received performance feedback suggesting that they performed badly (S-feedback effect, t_{23}=-2.344 ; p=0.0281). The performance feedback for the relevant other (O) also had an influence on S-pChange, depending on the social context in which it was observed. Subjects changed their performance more when the O received negative performance feedback in cooperation (just as they did for themselves) and positive performance feedback in competition (O-feedback x Context, t_{23}=-2.246 ; p=0.0346). The beta weights of this latter regressor were tested in Figure 4B (*O-influence on S-pChange*) and correlated with behavior in Figure 4D. Note that, for visualization purposes, the effect is sign-reversed in Figure 4D. **(B)** Number of false start trials per participant per minigame. **(C)** Signed minigame performances plotted for all subjects for all trials (black crosses), mean signed performance per subject (red dots) and mean signed performance for the whole sample (horizontal dark blue line; shaded dark blue indicates

standard error of the mean). False start performances are not shown. Note that the figure shows signed performance values, because although the absolute value is enough to indicate performance levels, the signed performances are necessary to calculate the change in performance from one trial of a minigame to the next trial of the same minigame (S-pChange, Supplemental Experimental Procedures 2). For both minigames, negative performance values indicate responses that were too quick and positive values indicate responses that were too slow. Zero indicates the optimal response time. Performances in time and color task were comparable with no differences in mean performance (t_{23}=-0.899; p=0.3778). This and the fact that the mapping of performance feedback schedules to minigames was balanced across subjects (11 received better performance feedback in the time task and 13 subjects received better performance feedback in the color task, see Figure S1A) makes it unlikely that S-pChange effects were driven by performance differences in the minigames. (error bars are mean +/-SEM; *, $p < 0.05$)

RL model fitted on ratings and engage/avoid decisions

Parameter	α	β
Median (SEM)	0.3906 (0.0359)	0.4684 (0.0489)

Table S1 (relating to Figure 2)

Supplemental reinforcement learning model results. Summary of parameter estimates (SEM is standard error of the mean).

Supplemental Experimental Procedures.

1. Reinforcement learning model

For every subject, we fitted a standard reinforcement learning model to model the performance estimates assigned to the three players trial by trial (Self, S; Other1, O1; Other2, O2). We used two minigame specific (Color Task, T1; Time Task, T2) performance estimates per player. The performance estimates summarize the previous performance history and are hence referred to as *performance*. They reflect the expected performance based on a recency-weighted average of past performance feedback. This resulted in six player and minigame specific *performance* estimates: $performance_{S\text{-}T1}$, $performance_{S\text{-}T2}$, $performance_{O1\text{-}T1}$, $performance_{O1\text{-}T2}$, $performance_{O2\text{-}T1}$, $performance_{O2\text{-}T2}$. On every trial t, the three *performance* estimates associated with the current minigame were updated using a prediction error (PE) based learning rule with a learning rate α as a free parameter:

1) $$Performance_{t+1} = performance_t + \alpha \times PE_t$$

(formula was applied separately for S, O1, O2, given T1 or T2)

The PE itself was calculated based on the specific *performance* estimate and performance feedback of the player in the current minigame as:

2) $$PE_t = feedback_t - performance_t$$

(formula was applied separately for S, O1, O2, given T1 or T2)

In false start trials, the *performance* estimate for S was not updated and remained unchanged until the next trial of the same minigame. No PEs for S were calculated for false start trials (the other players never displayed false start trials). For the first trial of the session for each player for each minigame, no *performance* estimates and prediction errors were calculated, but the initial performance feedbacks were used as the starting *performance* estimates for the respective player in the respective minigame.

In each trial after the four starter trials, subjects made a decision about cooperating or competing (depending on the current context) with the relevant other (O) and in addition provided ratings of both S and O. Both engage/avoid decisions and ratings were modeled based on *performance* estimates for S and the O, called *S-performance* and *O-performance*. *S-performance* is the *performance* estimate for Self associated with the minigame of the current trial ($performance_{S\text{-}T1}$ or $performance_{S\text{-}T2}$). Similarly, *O-performance* refers to $performance_{O\text{-}T1}$, $performance_{O1\text{-}T2}$, $performance_{O2\text{-}T1}$ and $performance_{O2\text{-}T2}$, depending on which other player was currently selected as the O and which minigame took place. Therefore, *S-performance* and *O-performance* represented minigame and player specific performance expectations of the players involved in the current trial's engage/avoid decision. The same was the case for the PEs associated with S and O.

Subjects' ratings of a player reflected expectations of whether they would perform either better or worse than a level indicated by a *rating marker* the position of which was adjusted from trial to trial using a staircase procedure explained in Experimental Procedures ("Ability ratings"). Expectations expressed in the ratings that exceeded or fell below the rating marker were referred to as positive and negative ratings, respectively. To calculate the probability of a positive rating (p(positiveRating)), we used a softmax function with an inverse temperature β. This was done separately for S and O using *S-performance* and *O-performance*, respectively as well as the player specific rating marker:

3) $$P(positiveRating) = \frac{\exp[\beta \times (performance - ratingmarker)]}{\exp[\beta \times (performance - ratingmarker)] + 1}$$

(this formula was applied separately for *S-performance* and *O-performance* given their respective rating markers)

Having calculated the probability of a positive rating on a given trial, the probability of the rating actually observed was derived, again, separately for S and O:

$$4)\ P(rating) = \begin{cases} p(positiveRating) & if\ rated\ positively \\ 1 - p(positiveRating) & if\ rated\ negatively \end{cases}$$

(formula was applied separately for S and O)

Subjects also received a small gain or loss at the end of a trial if they had made a positive rating and the expectation indicated by that rating had been accurate (ratingbonus of 0.25 points). As explained in the Experimental Procedures, to ensure that there was no temptation to perform poorly in the task no ratingbonus was awarded when a negative rating had been given. The expected value of a rating (EV_{rating}) was calculated as

$$5)\ EV_{rating} = \begin{cases} [p(positiveRating) - 0.5] \times 2 \times ratingbonus & if\ rated\ positively \\ 0 \quad if\ rated\ negatively \end{cases}$$

(formula was applied separately for S and O)

Note the bounds of EV_{rating} for positive ratings are 0.25 and -0.25, which are the points that can be lost or won for positive ratings.

In addition to completing a rating for S and O on each trial, subjects made a decision to engage in or avoid cooperating/competing. Given the objective social context specific payoff scheme of the task (equations 1a and 1b in Experimental Procedures section), the subjective expected value of engaging in cooperation/competition (EV_{engage}) was calculated in an analogous way:

$$6a)\ Competition: \quad EV_{engage} = S\text{-}performance - O\text{-}performance - threshold$$

$$6b)\ Cooperation: \quad EV_{engage} = (S\text{-}performance + O\text{-}performance) / 2 - threshold$$

A decision to avoid cooperating/competing led to a gain of 1.5 points and a loss of 1.5 points with equal probability (see " Experimental design and schedule" in Experimental Procedures) and subjects had been instructed that the expected value of the decisions to avoid cooperating/competing was zero:

$$7)\ EV_{EAD} = \begin{cases} EV_{engage} & if\ engage \\ 0 \quad if\ avoid \end{cases}$$

Therefore, EV_{engage} was used as decision variable for the engage/avoid decisions to calculate the probability of engaging in cooperation or competition:

$$8)\ P(engage) = \frac{\exp(\beta \times EV_{engage})}{\exp(\beta \times EV_{engage}) + \exp(\beta \times EV_{avoid})}$$

Note that EV_{avoid} is zero in equation 8, as explained above. The probabilities of the actual choices made were derived from p(engage):

$$9)\ P(choice) = \begin{cases} P(engage) & if\ engage \\ 1 - P(engage) & if\ avoid \end{cases}$$

The full reward expectation on each trial (EV_{chosen}) was defined as the sum of the expected values from both ratings and the expected value of the engage/avoid decision (equations 5 and 7):

$$10)\ EV_{chosen} = EV_{S-Rating} + EV_{O-Rating} + EV_{EAD}$$

The reward prediction error (RPE) was calculated based on all reward outcomes of a trial including both rating reward outcomes and the engage/avoid decision reward outcome (see above equation 2 for the calculation of player specific prediction errors):

11) $RPE = \text{Reward} - EV_{chosen}$

Overall, the free parameter set θ comprised two free parameters: the learning rate α and the inverse temperature β. We fitted these parameters for every subject separately by minimizing the negative log likelihood (nLL) over all trials N, given a set of parameter values. For the calculation of nLL, we treated ratings and engage/avoid decisions equally. So the decisions used to fit the model included equal proportions of ratings of S, ratings of the O and engage/avoid choices to improve the model fit.

12) $nLL = -\sum_{n=1}^{N} \log(p(decision_t | \theta))$

2. Behavioral analyses

We used general linear models (GLM) to examine the impact of different factors on behavior. GLMs were applied to the ability ratings for (S and O ("Rating GLM for S/O"), to the engage/avoid choices ("Choice GLM") and to an index of true performance change over trials ("pChange GLM"). For all GLMs, all regressors were normalized (mean of 0 and standard deviation of 1).

Our first analyses aimed to show that subjects relied on performance feedback for their ratings and engage/avoid choices. For this, we predicted S and O ratings on the basis of S's and O's recent performance feedback history (last four trials; referred to as "feedback" below) at trial t. In addition, the GLMs contained the value indicated by the rating marker (the rating marker indicated a performance level with reference to which the rating should be made; see "Ability ratings" in Experimental Procedures):

Rating GLM 1 for S:
feedback-S_{t-1}, feedback-S_{t-2}, feedback-S_{t-3}, feedback-S_{t-4}, ratingmarker-S (Figure 2B-i)

Rating GLM 1 for O:
feedback-O_{t-1}, feedback-O_{t-2}, feedback-O_{t-3}, feedback-O_{t-4}, ratingmarker-O (Figure 2C-i)

As an aggregate index of performance feedback, we fitted a reinforcement learning (RL) model on the rating data (see Supplemental Experimental Procedures 1 for details on the RL model) and used *S-performance* (recency-weighted performance estimate for self) and *O-performance* (recency-weighted performance estimate for relevant other) to explain engage/avoid decisions. For this analysis, the RL model was fitted on the ratings only and not on the engage/avoid decisions (all other analyses including neural analyses were fitted on both ratings and choices to improve model fit). We applied the same GLM separately to cooperative and competitive trials:

Choice GLM1 - binned by social context (cooperate/compete):
S-performance, O-performance, threshold (Figure 2D)

The second set of analyses aimed to investigate more subtle effects of self-other-mergence (SOM) in the rating data. Again, we used *S-performance* and *O-performance* from the RL model. Note that the SOM GLM effects are orthogonal to the fitting done in the RL model and all SOM related effects we present in this manuscript can be shown without the use of a reinforcement learning model i.e. by using past performance feedback directly to explain choices (Figure 4B and S6A, Figure S4B). The following analysis was restricted to engage trials only (a supplementary analysis with the same results using all trials including those on which subjects refrained from cooperation and competition can be found in Figure S4C). We focused first on engage trials because the social context is critical on these trials (rather than on "avoid" trials when subjects simply took the default option of a random payment). The GLM was applied separately to cooperate and compete trials:

Rating GLM2a for S - binned by social context (cooperate/compete):
S-performance, *O-performance,* ratingmarker-S (Figure 2B-ii)

Rating GLM2a for O - binned by social context (cooperate/compete):
O-performance, *S-performance,* ratingmarker-O (Figure 2C-ii)

Note we used *S-performance* in the S rating and *O-performance* in the O rating as control parameters and the resulting beta weights for those regressors were only used as indices of individual variability, for instance to establish correlations with brain signals; we did not test such effects for significance as they had been fitted on the same ratings. The main aim of this GLM was to assess whether ratings indicated, in addition to agent-consistent effects, an inappropriate influence of the performance history of the different player (underlined above; for example an influence of *O-performance* on the S rating, or an influence of *S-performance* on the O rating). The binned rating GLM2a was used for visualization. However, as we were interested in the differences of agent misattributed effects in cooperate and compete conditions (i.e. the interaction by social context), we devised an analogous rating GLM2b that was not binned by social context, but instead contained interaction terms of regressors of interest with social context. These interaction effects were tested for significance of SOM_{int} ("int" denotes "interaction"). To calculate the interaction terms, *S-performance x Context* for

example, *S-performance* was normalized and multiplied with 1 for cooperate trials and -1 for compete trials (the number of cooperate and compete trials was identical over the whole session); the same was done for *O-performance*. Hence, a positive interaction effect indicated that the effect of S-performance was stronger in cooperation than in competition. For analyses restricted to engage trials the 1/-1 term indicating cooperation or competition was normalized as well to account for possible differences in trial number. GLM2b, like GLM2a, was restricted to engage trials.

Rating GLM2b for S - with interaction by social context:
S-performance, O-performance, S-performance x Context, O-performance x Context, Context, ratingmarker-S
(SOM_{int}(O-->S) is underlined; Figure S4A)

Rating GLM2b for O - with interaction by social context:
S-performance, O-performance, S-performance x Context, O-performance x Context, Context, ratingmarker-O
(SOM_{int}(S-->O) is underlined; Figure S4A)

Additional versions of rating GLM2b that did not use an RL model (Figure S4B) or are based on all trials (instead of engage trials only; Figure S4C) are presented in Figure S4.

We also analyzed performance in the minigames (see Figure S3 for details on the minigames and their performance definitions). For these analyses, we used a measure of subjects' true performance change (S-pChange) from any trial, t, to the following trial of the same minigame, t+1. S-pChange for a given trial t was calculated as the logarithm of the absolute performance difference:

$$S\text{-}pChange_t = \log(1 + |performance_{t+1} - performance_t|)$$

The performance measure was signed with zero indicating optimal performance while positive and negative values indicated responses that were too slow or too quick, respectively (Figure S6). The use of predetermined performance feedback schedules meant that performance feedback could not be improved by more optimal performance in the minigames (except false start trials as a case of veridical performance feedback, see Figure S2C and Figure S6B showing the number of false start trials per subject). This made it possible to use S-pChange as a measure of behavioral adjustments based on performance feedback. Performances from false start trials were treated as outliers and therefore no S-pChange for a false start trial and the trial directly preceding it were calculated. Note that the performance measures for both minigames were comparable and the performance feedback schedules were balanced over both minigames (Figure S6). The main aim of this set of analyses was to investigate whether behavioral adjustments were also influenced by performance feedback for the O depending on the social context (cooperation or competition). For this, similar to rating GLM2, we used two analogous versions of a GLM; one applied to cooperate and compete trials separately for visualization, and one version applied to all trials to test interaction by social context effects for significance. We investigated the relationship of S-pChange (for trial t indicating the transition from trial t to t t+1, see definition of S-pChange above) to the three variables that determine reward outcomes in engage/avoid decisions: S performance feedback, O performance feedback and threshold (all for trial t)

S-pChange GLM1a - binned by social context on trial t (cooperate/compete):
S-feedback, O-feedback, threshold (Figure 4B)

As in previous analyses, significance of effects was calculated over all trials, using the interaction effect of performance feedback and social context on trial t.

pChange GLM1b - all trials:
S-feedback, O-feedback, S-feedback x Context, O-feedback x Context, Context, threshold
(SOM-related effect is underlined; Figure S6A)

Note that when using the threshold regressor over all trials, we combined the threshold regressors from cooperate and compete conditions and normalized them separately for each condition, as in the following neural analyses. The resulting regressor was also again normalized to have a mean of zero

and standard deviation of 1. S-pChange was normalized separately for individual subjects in these GLMs. Positive interactions by context again indicated stronger influence of the performance feedback on cooperate compared to compete trials.

3. MRI data acquisition and analysis

3.1 MRI data acquisition and preprocessing. Imaging data were collected on a 3 Tesla Siemens MRI scanner using a 32 channel head coil. T1-weighted structural images were acquired with the settings TR=3sec, TE=4.75mssec, TI = 1100msec, 1x1x1mm voxel size, 256x176x224 grid. Functional images were acquired using a Deichmann echo-planar imaging (EPI) sequence with TR=3s, TE=30 ms, 3x3x3mm voxel size, 87° flip angle, 30° slice angle and z-shimming to reduce signal distortions as well as dropout in medial orbitofrontal areas (Deichmann et al., 2003).

We used FMRIB's Software Library (FSL) to analyze imaging data (Smith et al., 2004). FMRI data preprocessing comprised spatial (Gaussian using full-width half maximum of 5 mm) and temporal filtering (3 dB cut-off at 100sec), motion correction with FSL's MCFLIRT and filtering of noise components after visual inspection using FSL's MELODIC. In a two-step procedure via subjects' individual structural MRI images, preprocessed functional data were nonlinearly registered to Montreal Neurological Institute (MNI) space.

3.2 MRI whole-brain analyses. We used FSL FEAT (Smith et al., 2004) for first level analyses. fMRI data were pre-whitened with FSL FILM to account for temporal autocorrelations. Motion regressors from MCFLIRT were included as nuisance regressors of no interest. Temporal derivatives of relevant regressors were included and the model was temporally filtered before it was applied to the data. Group results on the second level were calculated using FSL FLAME 1 with outlier de-weighting and a cluster-forming threshold of $z>2.5$ and $p<0.05$.

We used a single fMRI GLM for whole brain analysis. The same design was used for subsequent region of interest (ROI) analyses. The GLM includes RL-based regressors, which were fitted individually on all ratings and engage/avoid decisions (see Supplemental Experimental Procedures 1 for details on the RL model). All parametric regressors were normalized (mean of zero, standard deviation of one). The two main phases of interest were the decision phase and the feedback phase.

The decision phase was defined as the time period from engage/avoid decision onset to the engage/avoid choice subjects made (phase 1,2 and 3 in Figure S2A-ii). We modeled the decision phase as a constant regressor and accompanying parametric regressors. The parametric regressors of interest comprised:

- *S-performance*
- *O-performance*
- *S-performance x Context,*
- *O-performance x Context,*
- Context (binary regressor; cooperation 1, competition -1)
- Threshold
- logRT

The two interaction terms and threshold were calculated as explained in the Supplemental Experimental Procedures section 2 "Behavioral analyses". logRT is the logarithm of the engage/avoid decision reaction time (see phase 3 in Figure S2A-i). The timing parameters for the parametric regressors were identical with the constant decision phase regressor, except for threshold and logRT. These regressors' onsets were delayed by one second, as the threshold was only revealed one second after engage/avoid decision onset and knowledge of the threshold was necessary to make an engage/avoid decision (see phase 2 in Figure S2A-i).

Decision phases from starter trials were excluded from the decision regressor and accounted for by a regressor of no interest. Correlations between parametric regressors are shown in Figure S5A (however, note that this correlation matrix also contains an additional regressor that was used in the ROI version of this design, $EV_{S\text{-}Rating+O\text{-}Rating}$, but not logRT, as the latter was not a regressor of interest).

We used two constant regressors with a duration of zero time-locked to the response of S and O rating to account for the rating events (Figure S5B-ii shows this effect for O). In addition we used parametric regressors accompanying these constant regressors accounting for the reward expectations associated with the ratings (EV_{rating} for S and O from equation 5 in Supplemental Experimental Procedures 1 for S rating and O rating, respectively).

The feedback phase was similarly modeled as a constant regressor and parametric modulators. Note that trial feedback was chunked in three components and presented in randomized order ("Feedback" in Experimental Procedures and Figure S2A-ii):

I) S and O performance feedback and rating reward outcomes
II) Engage/avoid decision reward outcome
III) Irrelevant other performance feedback

Duration of the constant feedback regressor was 2.5 seconds, the time window in which the three feedback components initially appeared (phase 2 onset to phase 4 onset in Figure S2A-ii). Parametric regressors were modeled as stick functions (i.e. duration of zero) time-locked to the appearance of the relevant feedback component. They comprised:

- *S-performance* (I)
- *O-performance* (I)
- S-PE (Prediction error for Self) (I)
- O-PE (Prediction error for relevant other) (I)
- O-PE x Context (I)
- Prediction error - irrelevant other (III)
- Context (I)
- S-pChange (I)
- EV_{chosen} (see equation 10; II)
- Reward prediction error (RPE; see equation 11; II)

Roman numerals in brackets after the regressors indicate to which feedback component a regressor was time-locked. Feedback phases from starter trials, from false start trials and from trials on which no S-pChange could be calculated (see "Behavioral analyses" in Supplemental Experimental Procedures 2) were excluded from the feedback regressor and modeled as events of no interest. Correlations between parametric regressors are shown in Figure S5.

In addition, the GLM contained three regressors of no interest. First, a regressor time-locked to all button presses, modeled as stick functions, to account for movement-related effects. Second, two regressors captured brain signals associated with each minigame, spanning the time period from minigame onset to response button press.

3.3 ROI analyses. ROIs had a radius of three voxels and were centered on peak voxels of significant clusters from the whole brain GLM (Table 1). For ROI analyses, we transformed MNI to subject space and extracted the pre-processed BOLD time courses, averaged per volume. The time courses were normalized, oversampled by a factor of 20 and time-locked to decision phase onset and feedback phase onset (same onset timings as the constant regressors in the whole-brain GLM). We applied a GLM to each time point and computed one beta weight per regressor and time point, resulting in a time course of beta weights for each regressor. We extracted individual variation in signal size at the time of the group peak signal in an analysis window of 4 to 13 seconds from decision and feedback phase onset to relate brain activity to behavior. All regressors were normalized (mean of zero, standard deviation of one) for all ROI GLMs.

The ROI GLM for the decision phase contained the parametric regressors as the whole brain GLM, and, in addition contained a parametric regressor that was the sum of the EV_{rating} (equation 5 in Supplemental Experimental Procedures 1, RL section) for S and O to account for the reward expectation associated with those events:

- *S-performance*
- *O-performance*
- *S-performance x Context,*
- *O-performance x Context,*
- Context (binary regressor; cooperation 1, competition -1)
- Threshold
- logRT
- $EV_{rating\text{-}S+rating\text{-}O}$

Note that the ROI GLM for the decision phase was restricted to trials in which subjects made and engage choice rather than to avoid cooperating/competing. We did this to relate peak signal sizes from effects of interest in this analysis to behavioral beta weights from rating GLM2b (Figure S4A), which was also calculated over engage trials only.

The feedback related ROI GLM contained the same feedback related parametric regressors as listed for the whole brain GLM:

- *S-performance*
- *O-performance*
- S-PE (Prediction error for Self)
- O-PE (Prediction error for relevant other)
- O-PE x Context
- Prediction error - irrelevant other
- Context
- S-pChange
- EV_{chosen} (see equation 10)
- Reward prediction error (RPE; see equation 11)

The GLM for the feedback phase was calculated over all trials. We investigated signal sizes at the time of the group peak of relevant regressors in relation to behavioral beta weights from S-pChange GLM1b (O-feedback x Context), which was also calculated based on all trials.

We used a leave-one-out procedure on the group peak signal of the beta time course to do significance testing and avoid temporal selection biases. For every subject, we took the average beta time course of the relevant regressor based on the remaining 23 subjects. We identified the (positive or negative) group peak in the analysis window of 4 to 13 seconds from phase onset and then took the beta weight of the remaining subject at the time of that peak. We repeated this for all subjects. Therefore, the resulting 24 "peak" beta weights were selected independently of the time course of the subject analyzed. We assessed significance using t-tests on these resulting beta weights. For correlations with behavioral beta weights, the individual neural beta weights at the time of the group peak were used.

To illustrate some of the correlations between neural beta weights and behavioral beta weights (Figure 3B-ii,iii, Figure 4D), we used a median split procedure, in which our group of subjects was subdivided in two groups of 12 subjects based on their neural beta weight being low (i.e. below the median) or high (i.e. above the median) at the time of the group peak. Mean and standard error of the behavioral beta weight of interest are shown in the bar plots of these figures (right hand side of Figure 3B-ii,iii, and right hand side of Figure 4D).

Supplemental References

Brewer, M.B. (1979). In-group bias in the minimal intergroup situation: A cognitive-motivational analysis. Psychol Bull 86, 307-324.

Deichmann, R., Gottfried, J.A., Hutton, C., and Turner, R. (2003). Optimized EPI for fMRI studies of the orbitofrontal cortex. NeuroImage 19, 430-441.

Denny, B.T., Kober, H., Wager, T.D., and Ochsner, K.N. (2012). A meta-analysis of functional neuroimaging studies of self- and other judgments reveals a spatial gradient for mentalizing in medial prefrontal cortex. J Cogn Neurosci 24, 1742-1752.

Faber, N.S., Häusser, J.A., and Kerr, N.L. (2015). Sleep Deprivation Impairs and Caffeine Enhances My Performance, but Not Always Our Performance: How Acting in a Group Can Change the Effects of Impairments and Enhancements. Pers Soc Psychol Rev.

Michael, E., de Gardelle, V., and Summerfield, C. (2014). Priming by the variability of visual information. Proc Natl Acad Sci U S A 111, 7873-7878.

Nieuwenhuis, S., Forstmann, B.U., and Wagenmakers, E.J. (2011). Erroneous analyses of interactions in neuroscience: a problem of significance. Nat Neurosci 14, 1105-1107.

Saxe, R. (2006). Uniquely human social cognition. Curr Opin Neurobiol 16, 235-239.

Smith, S.M., Jenkinson, M., Woolrich, M.W., Beckmann, C.F., Behrens, T.E.J., Johansen-Berg, H., Bannister, P.R., De Luca, M., Drobnjak, I., Flitney, D.E*., et al.* (2004). Advances in functional and structural MR image analysis and implementation as FSL. NeuroImage 23 Suppl 1, S208-219.

Stasser, G., and Titus, W. (1985). Pooling of Unshared Information in Group Decision-Making - Biased Information Sampling during Discussion. J Pers Soc Psychol 48, 1467-1478.
